# The Genus *Alchornea* (Euphorbiaceae): A Comprehensive Review of Its Taxonomy, Traditional Uses, Phytochemistry, Pharmacological Potential, and Toxicology

**DOI:** 10.3390/molecules31101726

**Published:** 2026-05-19

**Authors:** Muhammad Aamer, Feibing Huang, Yi Long, Xudong Zhou, Yuqing Jian, Muhammad Iqbal Choudhary, Bin Li, Wei Wang

**Affiliations:** 1 TCM and Ethnomedicine Innovation and Development International Laboratory, Innovative Materia Medica Research Institute, School of Pharmacy, Hunan University of Chinese Medicine, Changsha 410208, China; m.aamer196267@gmail.com (M.A.); huangfeibing@stu.hnucm.edu.cn (F.H.); 20233742@stu.hnucm.edu.cn (Y.L.); xudongzhou999@hnucm.edu.cn (X.Z.); cpujyq@hnucm.edu.cn (Y.J.); 2 H. E. J. Research Institute of Chemistry, International Center for Chemical and Biological Sciences, University of Karachi, Karachi 75270, Pakistan; iqbal.choudhary@iccs.edu; 3 Dr. Panjwani Center for Molecular Medicine and Drug Research, International Center for Chemical and Biological Sciences, University of Karachi, Karachi 75270, Pakistan

**Keywords:** *Alchornea*, Euphorbiaceae, ethnomedicine, phytochemistry, pharmacological effects, toxicology

## Abstract

The genus *Alchornea* Sw. belongs to the Euphorbiaceae family. *Alchornea* species are commonly used in traditional medicine to treat inflammation; infectious, gastrointestinal, respiratory, musculoskeletal, and dermatological disorders; as well as other diseases. This comprehensive review provides an overview of recent scientific findings on the taxonomy, ethnomedicinal uses, phytochemistry, pharmacological potential, and toxicology of the *Alchornea* species. The literature was searched using SciFinder^n^, Google Scholar, and PubMed. The taxonomy of all reported plants was authenticated using “Plants of the World Online”. Studies were examined and categorized according to the genus’s taxonomic classification, traditional uses, phytochemistry, pharmacological potential, and toxicity. Phytochemical studies have identified **396** bioactive compounds, primarily triterpenoids, alkaloids, flavonoids, and phenolics. Pharmacological studies have reported significant antimicrobial, anti-inflammatory, antioxidant, anti-plasmodial, and cytotoxic effects. Nevertheless, toxicological statistics are limited and vary among species and extracts. The genus *Alchornea* exhibits significant pharmacological potential, as evidenced by its traditional uses. In comparison, the genus remains underexplored in terms of detailed mechanistic pharmacological evaluation. Studies of chemical constituents and biological activities have been conducted for only approximately 17 species. To translate the pharmacological potential of the genus *Alchornea* into clinical practice, a strategic focus on modern plant valorization is required. Future research should focus on the valorization of *Alchornea* species by developing standardized oral formulations and topical preparations that harness their validated anti-inflammatory and antimicrobial effects beyond traditional uses. However, these findings suggest that further research is needed to assess the efficacy and safety of the largely unexplored genus *Alchornea*.

## 1. Introduction

The family Euphorbiaceae (known as the spurge family) belongs to the major group of angiosperms (Magnoliophyta). It comprises approximately 299 genera and over 8000 species, with a worldwide distribution. Members of this family exhibit notable morphological diversity, comprising trees, shrubs, and herbs, with many species adapted to a wide range of ecosystems [1,2]. Euphorbiaceae is consistently ranked among the top three plant families with the highest percentage of medicinally used species worldwide [3].

**Figure 1:**
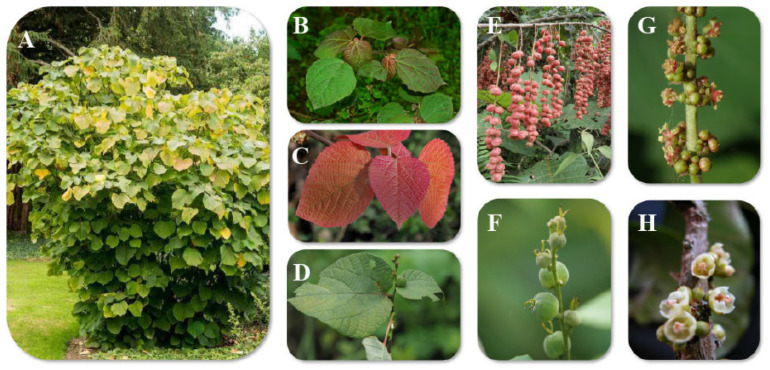
Some of the reported *Alchornea* species. Whole plant of *Alchornea Davidii* (**A**), leaves of *A. trewioides* (**B**), leaves of *Alchornea Davidii* (**C**), leaves of *A. tiliifolia* (**D**), fruits of *A. cordifolia* (**E**), flowers of *A. tiliifolia* (**F**), flowers of *A. trewioides* (**G**), and flowers of *A. triplinervia* (**H**).

The genus *Alchornea* Sw. belongs to the Euphorbiaceae family. It is widely distributed across tropical and subtropical regions, including Africa, South and Southeast Asia, Latin America, Australia, and various oceanic islands [4,5,6]. Species in this genus are typically distinguished by their simple, alternate leaves, unisexual flowers, and capsular fruits (Figure 1) [7,8]. Genera in the Euphorbiaceae family are often characterized by the presence of latex, a milky sap that can contain toxic diterpenes and phorbol esters known to cause skin irritation and inflammation. However, the genus *Alchornea* appears to be less toxic than other genera in the family, although an extensive safety assessment remains warranted.

Several species of *Alchornea* are traditionally used in ethnomedicine to treat a wide range of diseases, including inflammation, hormonal imbalances, infertility, urinary and respiratory tract infections, gastrointestinal problems, malaria-like fevers, hepatitis, and eczema [6,9,10,11,12,13,14]. Pharmacological studies have confirmed that several species of the genus *Alchornea* exhibit diverse biological activities, validating many of their ethnopharmacological uses worldwide, particularly in West and Central Africa. Bioactive compounds have been isolated and identified from various plant parts, including leaves, stem bark, and roots, supporting their medicinal potential [6].

Over the past few decades, numerous studies have examined the traditional uses, phytochemistry, and pharmacological potential of species in the genus *Alchornea*. However, to date, only one review, published in 2017, has summarized the ethnopharmacological uses and phytochemistry of *Alchornea* species [6]. The genus has not been extensively investigated, and substantial gaps remain in current knowledge, particularly regarding taxonomy, phytochemistry, pharmacological activities, and safety profile. No recent or comprehensive review has fully addressed the scope of research across its diverse *Alchornea* species. Hence, a comprehensive review is required to facilitate subsequent researchers’ understanding of existing research on the traditional uses, phytochemistry, pharmacology, and toxicology of the *Alchornea* species. This comprehensive review was undertaken to provide recommendations for future research to establish the scientific foundations necessary to support the growth and use of this genus. This search included all peer-reviewed publications through March 2026 that addressed *Alchornea* species in ethnobotany, phytochemistry, medicinal potential, and toxicology.

## 2. Taxonomy

The number of species in the genus *Alchornea* Sw. is estimated using various approaches. According to “Plants of the World Online” (https://powo.science.kew.org accessed on 15 April 2026), the genus *Alchornea* comprises 55 species with accepted names (15 April 2026). Meanwhile, the “Flora of China” reports 50 species (http://www.efloras.org). The estimated number of accepted *Alchornea* species varies significantly in the literature. Some researchers cite 55 accepted species [6,14], while others mention 40 [15], 42–62 [16], or 60 species [17]. Based on molecular phylogenetic studies and recent taxonomy, the genus *Alchornea*, along with other genera such as *Aparisthmium*, *Bocquillonia*, and *Concebeiva*, belongs to the tribe *Alchorneae* and the subfamily Acalyphoideae within the Euphorbiaceae family. The family Euphorbiaceae comprises 299 genera and over 8000 species, mainly distributed in subtropical and tropical regions. Key morphological traits include the production of milky latex, typically alternate leaves with stipules and often with basal or petiolar glands, and simple and unisexual flowers with a reduced perianth. The genus *Alchornea* consists mainly of shrubs or small trees, with most species being dioecious. The plant’s surface bears star-shaped, simple hairs, which are useful for distinguishing species. Leaves are arranged along the branches, with leaf stalks and small, often absent, stipules. Leaf blades show either pinnate or palmate veins, and many species exhibit three prominent veins arising from the base. Flower clusters arise from the leaf axils and form spike-like racemes; in some species, the male clusters may be branched. Each bract supports one to three female flowers or one to several male flowers, and the bracts lack glands. Female flowers are stalkless to short-stalked; the calyx usually has four overlapping lobes. Petals and a disc are absent. The female gynoecium consists of two carpels, each containing one ovule. Male flowers are nearly stalkless; at flowering, the calyx splits into two to five non-overlapping lobes. Petals are absent, but a central disc fuses with the base of the stamens. The styles are separate, long, and unbranched. The fruit is a dry capsule that splits into two one-seeded sections, leaving a central column after opening. Seeds are covered with small bumps and lack a fleshy appendage. A prominent ridge runs along the seed. Endosperm is present, and the embryonic leaves are thin, broad, and flat. The intact fruit typically appears three-lobed (Figure 1). *Alchornea* is closely related to *Aparisthmium* and *Conceveiba* [18,19]. Although these studies have not included all taxa of the genus, *Alchornea* is considered the most significant and widespread genus, and it is recognized as paraphyletic [20]. Muller was the first to make a subgeneric division, but included many taxa now classified as separate genera. Pax and Hoffmann distinguished three sections: Section *Alchornea* (‘*EuAlchornea*’ Mull. Arg.) has stellate hairs and occurs only in the Neotropics (except for the African *Alchornea cordifolia* (Schumach.) Mull. Arg.). The other two sections have simple hair in Africa and Southeast Asia. Section Cladodes (Lour.) Mull. Arg. lacks stipellae at the leaf base, has penninerved leaves, and generally has short petioles. Section *Stipellaria* (Benth.) Mull. Arg. Features stipellae, triplinerved leaves, and usually long petioles [21]. Reference [22] described the genus *Alchornea* with a single species, *Alchornea latifolia* Sw. The name commemorates Stanesby Alchorne (1727–1800), an English botanist and plant collector. Typically, the genus is characterized by the androecium rather than by the pistillate flowers. The stamens are inserted on a ring-like elevation of the receptacle, with the anthers dorsifixed and two-locular [21,22,23]. *Alchornea javanensis* (Blume) Mull. Arg, used in the literature, was later identified as a synonym of *Alchornea rugosa* Mull. Arg. (https://wfoplantlist.org) [23]. Table 1 lists all the accepted scientific names and synonyms of reported *Alchornea* species.

## 3. Traditional Uses

**Figure 2:**
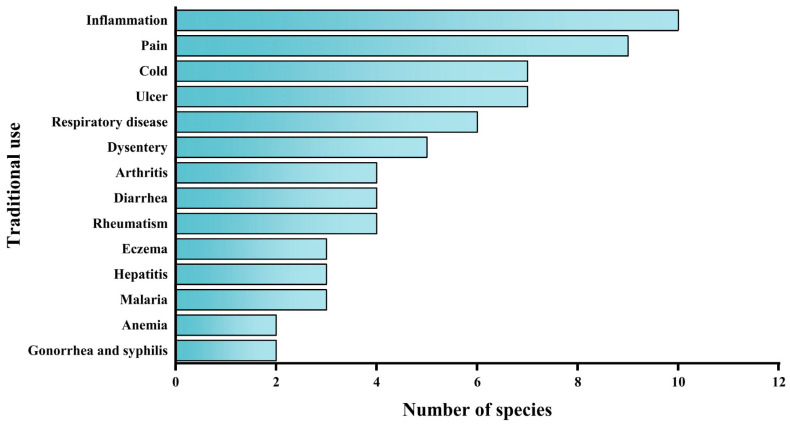
Traditional uses of *Alchornea* species.

A literature survey study on the genus *Alchornea* reveals that thirteen species have been documented for their traditional uses, particularly in regions of Africa (Nigeria, Senegal, Cameroon, Burkina Faso, and Tanzania), Asia (China, Japan, Vietnam, India, and Thailand), and parts of Central and South America (Brazil, Venezuela, Colombia, Mexico, and Peru). Across these regions, *Alchornea* species are commonly used in traditional medicine to treat diverse diseases, including inflammatory and infectious conditions, gastrointestinal and respiratory disorders, musculoskeletal diseases, dermatological conditions, hematological disorders, and sexually transmitted infections. The current review presents a comprehensive study of the ethnomedicinal uses of *Alchornea* species, highlighting their traditional significance across various geographic zones and cultures (Table 2 and Figure 2).

In Africa, several species of the genus *Alchornea* are specifically used as traditional therapeutic agents in various conditions. In particular, species such as *A. cordifolia*, *A. floribunda*, *A. laxiflora*, and *A. hirtella* are among the most widely used in ethnomedicinal practices. *A. cordifolia* is prominently highlighted in the traditional pharmacopeia of many African countries. It is used to treat diverse diseases, including inflammation, wounds, hemorrhoids, rheumatism, arthritis, toothache, sexually transmitted diseases, and malaria [9,11,24,25,26]. In addition to its medicinal value for humans, *A. cordifolia* is ecologically important as a preferred foraging plant for the okapi (*Okapia johnstoni*), an endemic mammal of the Congo. Observational evidence suggests that the Okapi’s choice of this plant is not trivial but is believed to be motivated by the need for self-care [27,28].

*A. floribunda* is also widely used in traditional Nigerian medicine, where the leaves, stems, and roots are used to treat inflammation, pain, eczema, hepatitis, and infectious diseases [29,30,31]. In Congo, aqueous extracts from leaves and root bark are used to treat human African trypanosomiasis (HAT). Similarly, in Cameroon, leaf decoctions are used to treat parasitic and bacterial infections [32,33]. Traditionally, all parts of *A. laxiflora* are used in various forms of folk medicine across Africa. Ethnomedicinal reports indicate its application in the treatment of inflammation, malaria, piles, dysentery, anemia, eczema, ringworm, typhoid fever, sexually transmitted infections, female infertility, tumors, and toothache [34,35,36,37,38]. In Asia, particularly in China, Thailand, and Vietnam, species such as *A. davidii*, *A. trewioides*, and *A. rugosa* are traditionally used to treat a wide range of diseases. These include inflammation, pain, prostate gland dysfunction, dysentery, shigellosis, chronic bronchitis, eczema, fever, skin wounds, fracture healing, malaria, and muscle regeneration [6,39,40,41,42,43,44,45,46].

Furthermore, in Brazilian traditional medicine, the following species, namely *A. triplinervia*, *A. latifolia*, *A. castaneifolia*, *A. glandulosa*, and *A. discolor*, are commonly used to treat diseases such as pain, inflammation, arthritis, gastric ulcers, colds, diarrhea, coughs, female infertility, rheumatism, tuberculosis, and gastritis, and are even cited for aphrodisiac and geriatric uses for males [10,47,48,49,50].

**Table 2:** Traditional uses of *Alchornea* species.

Sr. No.	Botanical Name	Vernacular Name	Country	Plant Part	Processing Method/Route of Administration	Traditional Uses	References
1	*A. castaneifolia*	“Iporuru”, “Sara”, “Gurupia”, “Jartio”	Brazil, Colombia, Peru, Bolivia, Argentina	Leaves and branches	Infusion, and smoked	Pain, ulcer, inflammation, arthritis, cold, cough, diarrhea, fertility in females, rheumatism, gastritis, aphrodisiac, and geriatric use for males	[10,47,51,52,53,54]
2	*A. cordifolia*	“Christmas Bush”, “Mbom”, “Ipa”, “Uwanwe”	Nigeria	Leaves, stem bark, and roots	Decoction, chewed, smoked, macerated, ablution, and snuffed	Skin disease, cough, fever, arthritis, rheumatism, diuretic, purgative, diabetes, gonorrhea, and syphilis	[55,56,57,58,59]
		“Feme”, “Dieca”, “Kotia”, “Yama”	Burkina Faso, Ivory Coast	Leaves		Gastroenteritis, anemia, malaria, bronchitis, cough, and pain	[60,61,62]
		“Ngimii”, “Gimii”, “Ira”	Senegal	Leaves and stem bark		Diarrhea, ulcer, pneumonia, cough, and cold	[7,63]
		“Njoekoi”	Sierra Leone	Leaves		Wounds, diarrhea, and dysentery	[64]
		“Christmas Bush”, “Mbom”, “Uwanwe”	Cameroon	Leaves and stem bark		Toothaches, sores, malaria, dysentery, dermatitis, anemia, and diarrhea	[65,66,67,68,69]
		“Mjururuzi”, “Omujururuzi”	Tanzania	Leaves, stem bark, and roots		Malaria, skin diseases, gonorrhea, and syphilis	[70]
		“Luzibazib”	Uganda (South)	Twigs and stems		Pre-hepatic jaundice, cold, pregnancy-related illness	[71]
		“Gyama”, “Asante-twi”	Ghana	Leaves and stem bark		Wounds, fractures, hemorrhoids, ulcers, Dermatitis, and ringworm	[72,73]
		“Koyiran”, “Towlen”, “Zikoye”, “Holenta”, “Pelena”	Republic of Guinea	Stem bark and roots		Snake bites, regulate menstruation, and act as a vermifuge	[13,74]
		“Bunzi-kibunzi”, “Popo”, “Kimbusil”	Democratic Republic of Congo	Leaves, twigs, stem bark, and roots		Dysentery, malaria, toothache, cough, piles, sickle cell disease, and backache	[75,76,77,78,79,80]
		“Tamada Kala”	Republic of Mali	Leaves		Malaria and sexually transmitted diseases	[81,82]
		“Mbogo”, “Galoa”, “Nkomi”, “Orungu”, “Nkabi”, “Bavarama”	Gabon	Leaves		Vomiting, toothaches, diarrhea, cicatrizing agent, urethral inflammation, conjunctivitis, and dysentery	[83,84,85,86]
3	*A. davidii*	“Shan Ma Gan”	China	Leaves	Decoction	Infectious or inflammatory diseases	[46,87]
4	*A. discolor*	“Palometa Huayo”, “Supiarana”, “Reventillo”	Peru, Brazil, Venezuela	Leaves	Decoction	Fish bait	[47,54,88]
5	*A. floribunda*	“Ononn”, “Ngombo”	Democratic Republic of Congo	Leaves and roots	Water extract, decoction, and powder	Aphrodisiacs, arthritis, inflammation, pain, cold, respiratory diseases, urinary disorders, antidote for poison, and wounds to the skin	[33,89,90,91,92]
		“Iporuru”	Nigeria	Leaves, stems, and roots		Eczema, hepatitis, pains, infectious diseases, and inflammatory disorders	[29,30,31,93]
			Central Africa	Leaves		Arthritis, rheumatism, piles, muscle pain, wounds, ringworm, eczema	[31,91,94]
			Cameroon	Leaf		Parasitic and bacterial infections	[32,33]
			Gabon	Roots and root bark		Aphrodisiac and strength	[12,95]
6	*A. glandulosa*	“Tapia”, “Tanheiro”, “Amor-seco”, “Canela-rosa”	Brazil	Leaves and aerial parts	Infusions	Inflammation, antiulcer agents, and gastritis	[96,97,98]
7	*A. hirtella*	“Tolo-geƞge”	Sierra Leone	Leaves, fruits, roots, and root bark	Decoction and chewed	Dysentery, pain, hepatitis, toothache, stomachache, diarrhea, and to treat tiredness	[99,100,101,102]
			Democratic Republic of Congo	Leaves		Fractures	[100,103]
8	*A. latifolia*	“Sweet Wood”, “Coton De Caribe”, “Escobo”, “Huampa”, “Loblob”	Brazil, Mexico, Colombia, Venezuela, Peru	Flowers, leaves, and twigs	Decoction	Cold and tuberculosis	[47,48,49]
9	*A. laxiflora*	“Lya”/”Pepe”	Nigeria	Whole plants, leaves, fruits, stems, roots, and barks	Decoction and infusions	Malaria, anemia, emmenagogue, ringworm, venereal disease, cold, Inflammatory and infectious diseases, sexually transmitted diseases, dysmenorrhea, oligomenorrhea, menorrhagia, amenorrhea, dysentery, eczema, cough, skin diseases, infertility in females, and diabetes	[14,34,38,104,105,106,107,108,109,110,111,112]
			South Africa, Democratic Republic of Congo	Leaves, stems, and branches		Infectious diseases, anti-tumor, inflammation, teething problems, and chewing sticks	[14,36,113]
			Ghana	Stems and branches		Treating toothaches	[37]
			Cameroon	Whole plants and leaves		Urinary tract infections, hepatitis, pains, epilepsy, anxiety, insomnia, dizziness, headaches, migraine, stomachache, dysentery, jaundice, and depression	[114,115,116,117,118]
10	*A. mollis*	“Badiki”, “Mao Guo Shan Ma Gan”	India, Nepal, China	Leaves, stem bark, and bark	Powered and juice	Skin diseases, rheumatism, arthritis, cold, pain, piles, and abortifacient	[119]
11	*A. rugosa*	“Alchorn Tree”	Vietnam, China, India, Australia,	Leaves, branches, roots, and seeds	Decoction	Malaria, cold, dysentery, wounds, fracture healing, enema, and muscle regeneration	[6,39,120]
12	*A. trewioides*	“Hong Bei Shan Ma Gan”	China	Leaves	Decoction and pills	Inflammation, prostate gland issues, Shigella, pain, alleviating illness, and discomfort	[43,44,45]
			Vietnam, Cambodia, Japan, Laos, Thailand	Branches and leaves		Dysentery, chronic bronchitis, urolithiasis, hemorrhage uterine, hemorrhage trauma, urticaria, and eczema	[40]
13	*A. triplinervia*	“Cascara de yuca”, “Canela-raposa”, “Ajumna”, “Cocopano”	Colombia, Brazil, Venezuela, Peru	Leaves and the aerial part	Tea form	Pain and diarrhea	[47,50]

## 4. Phytochemistry

**Figure 3:**
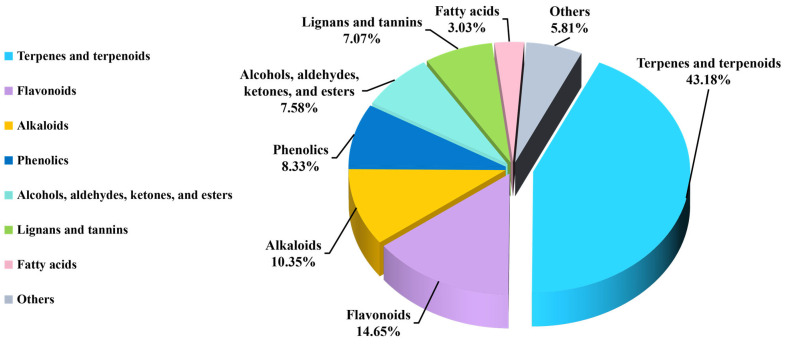
Proportions of compound classes in the genus *Alchornea*.

**Figure 4:**
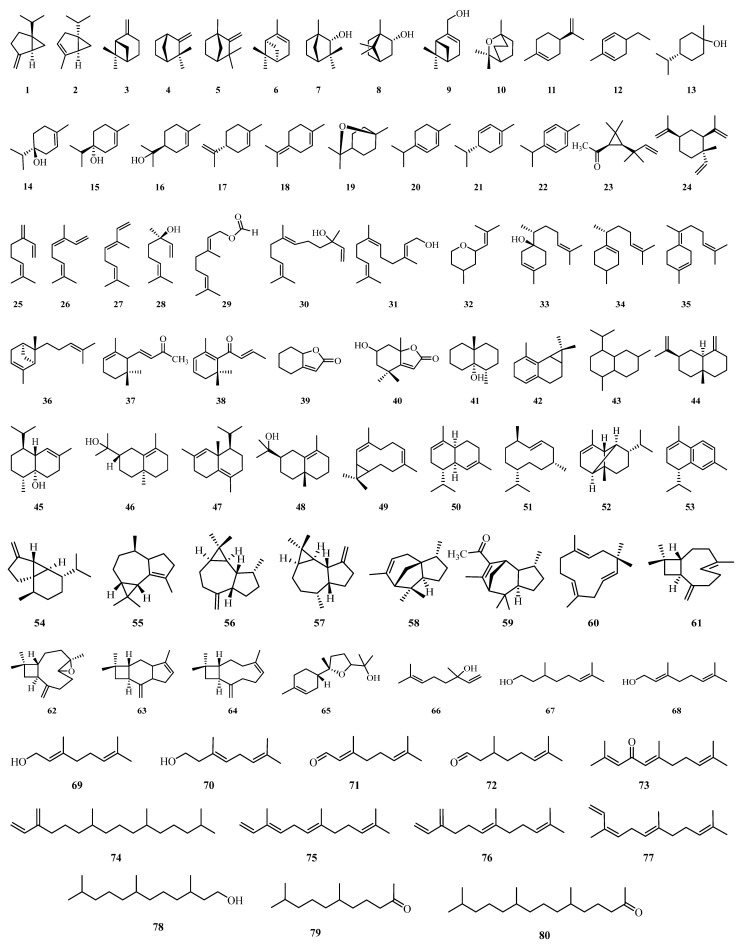

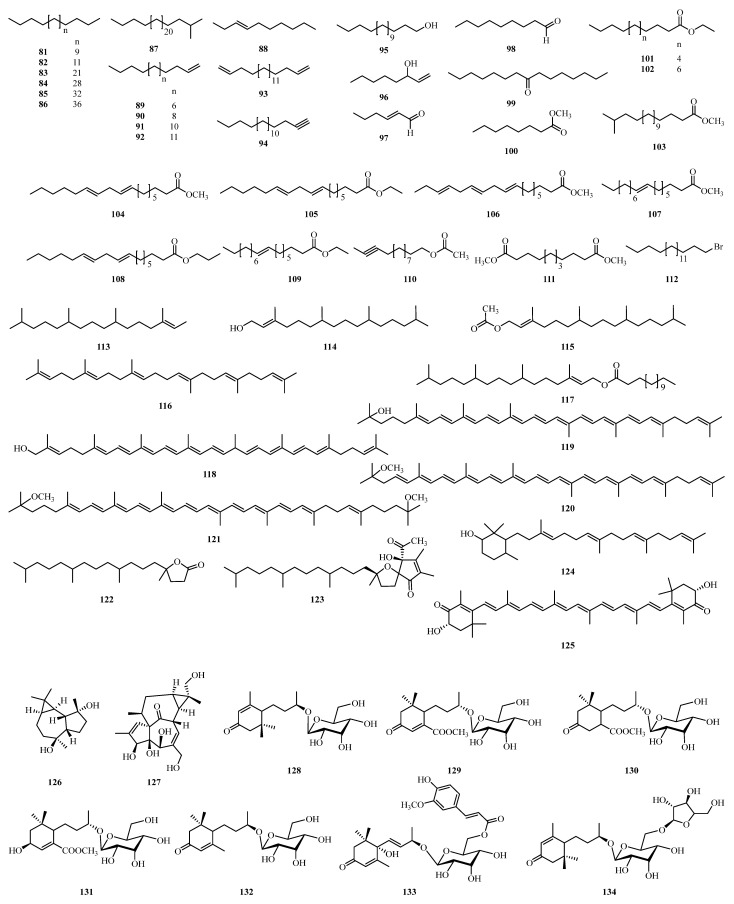

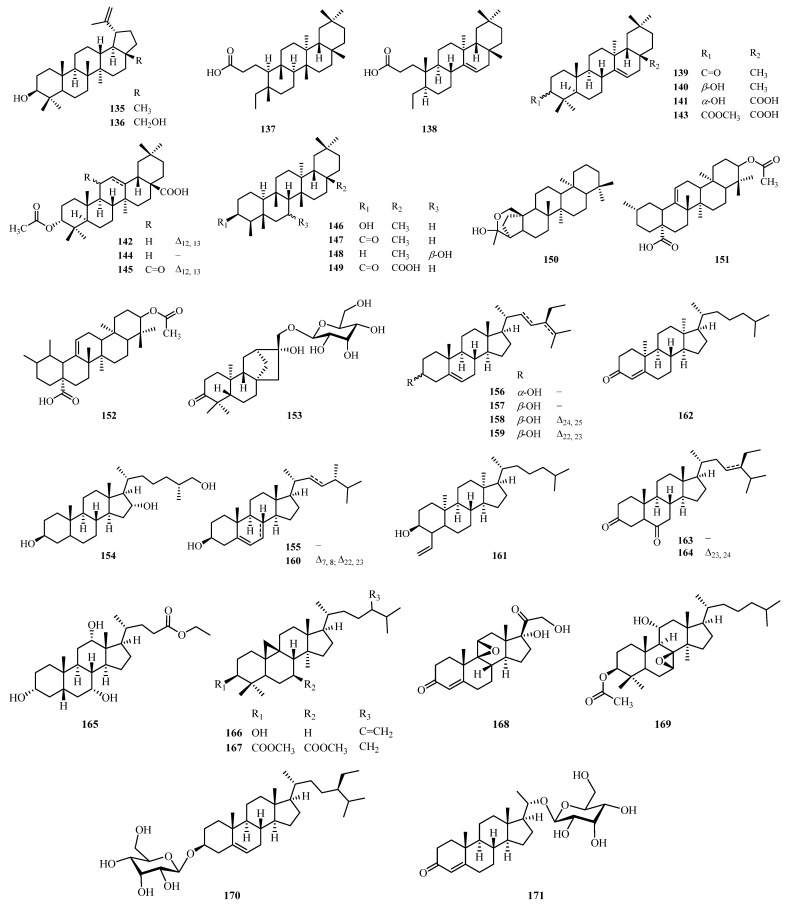
Terpenes and terpenoids (**1–171**) from *Alchornea* species.

**Figure 5:**
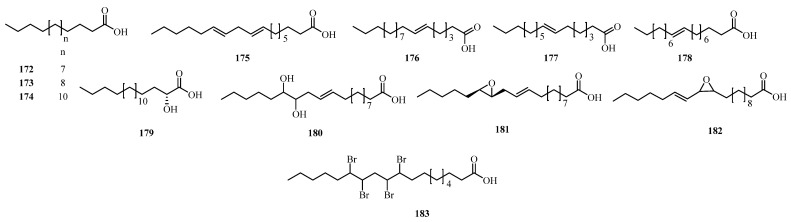
Fatty acids (**172–183**) from *Alchornea* species.

**Figure 6:**
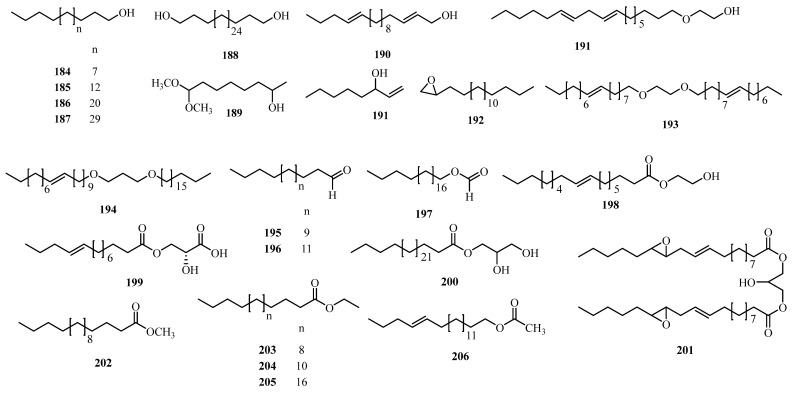

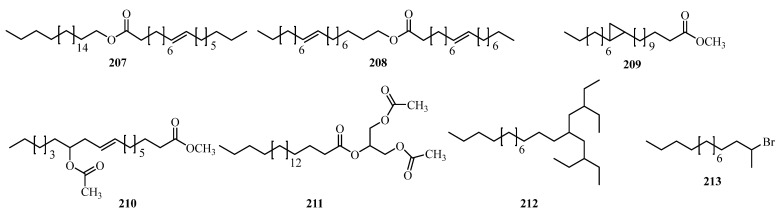
Alcohols, aldehydes, ketones, and esters (**184–213**) from *Alchornea* species.

**Figure 7:**
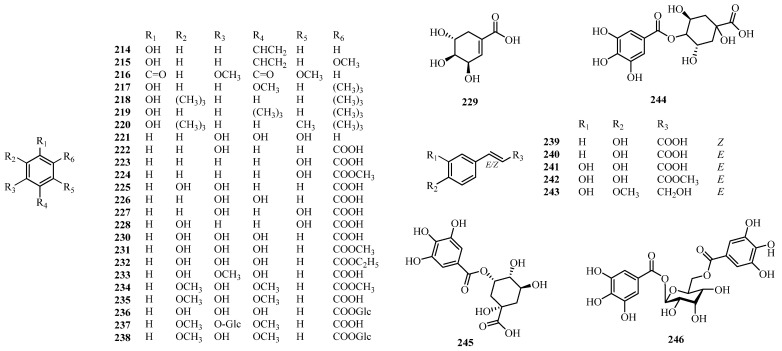
Phenolic compounds (**214–246**) from *Alchornea* species.

**Figure 8:**
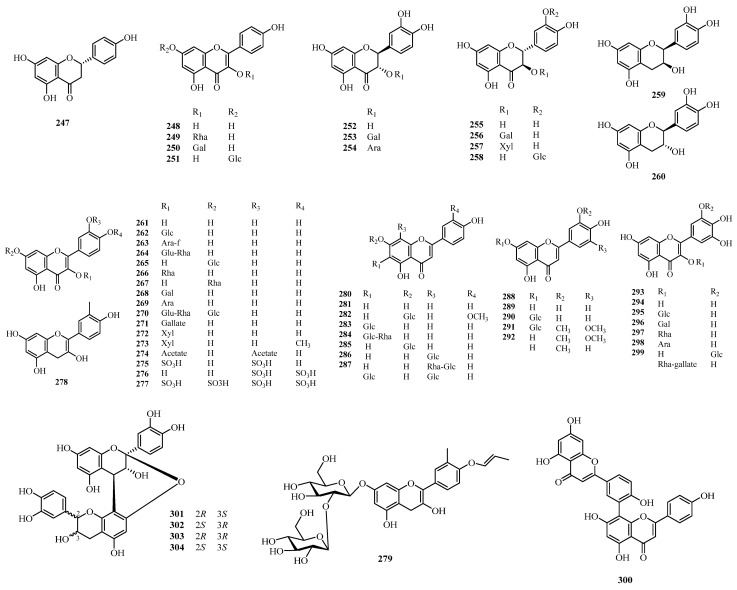
Flavonoids (**247–304**) from *Alchornea* species.

**Figure 9:**
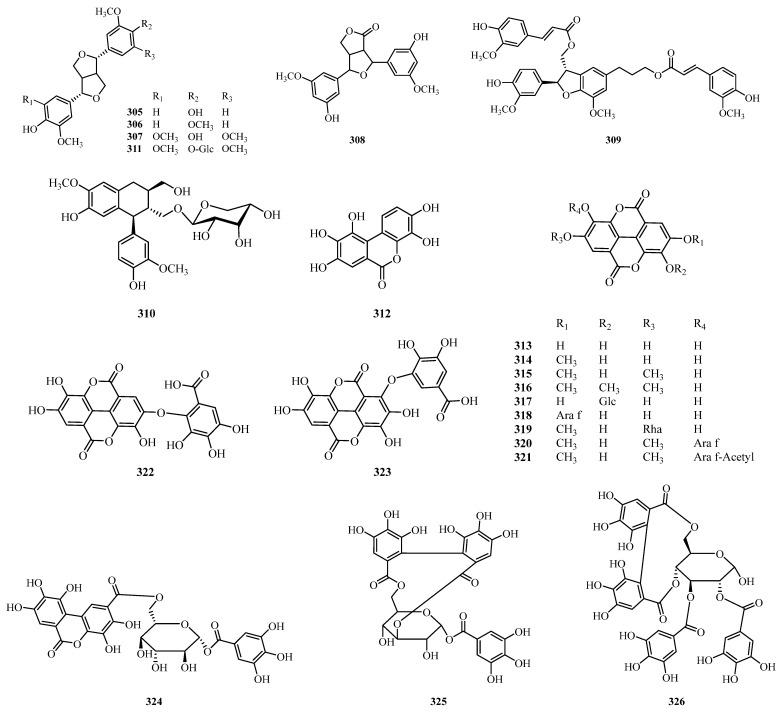

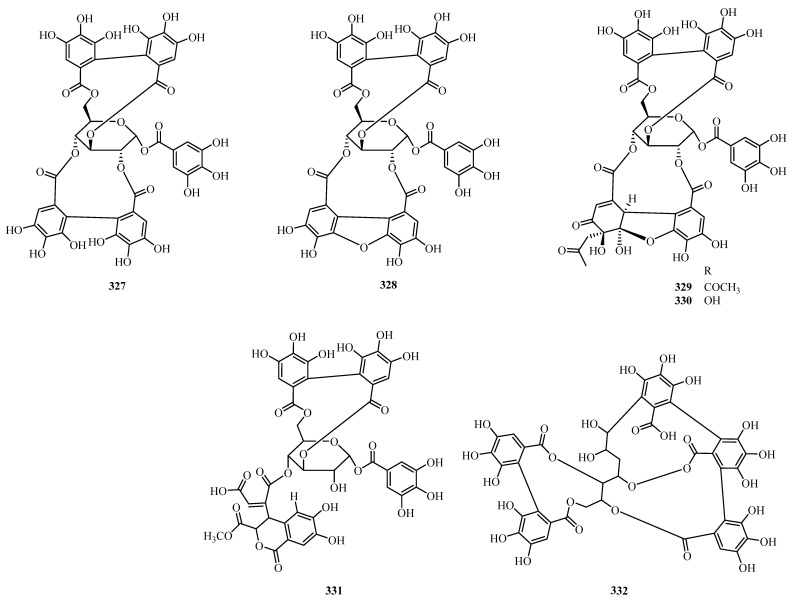
Lignans and tannins (**305–332**) from *Alchornea* species.

**Figure 10:**
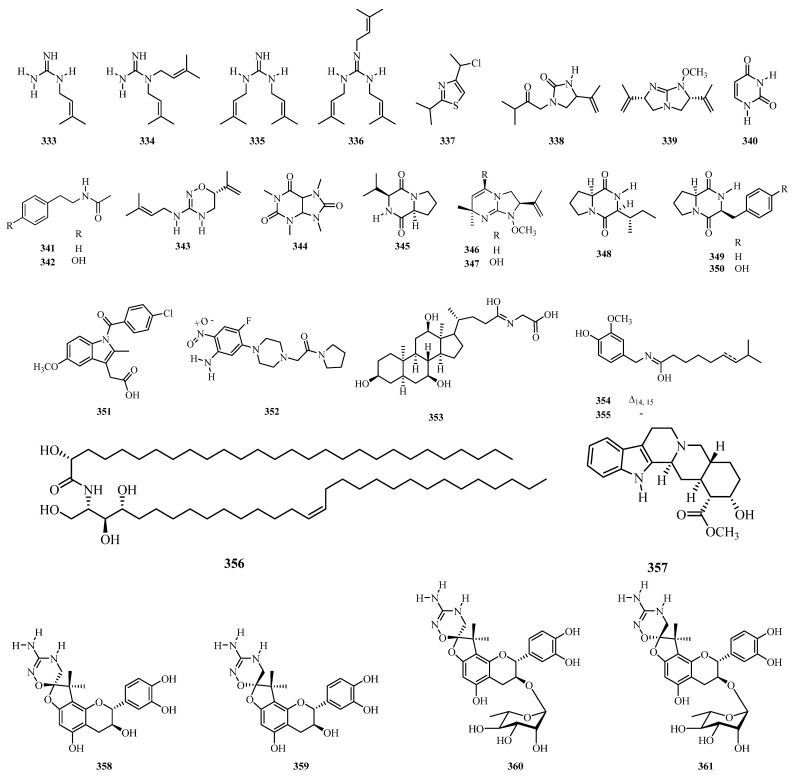

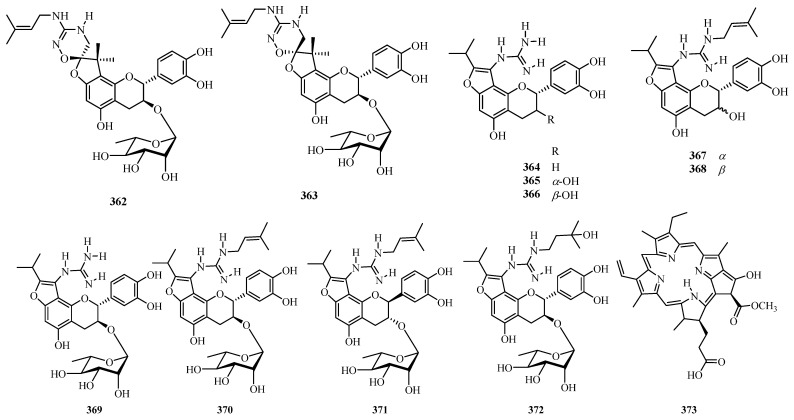
Alkaloids (**333–373**) from *Alchornea* species.

**Figure 11:**
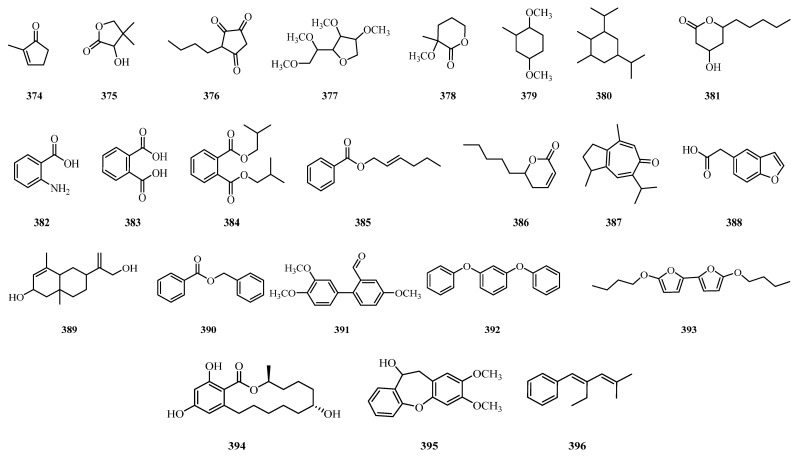
Other compounds (**374–396**) from *Alchornea* species.

**Figure 12:**
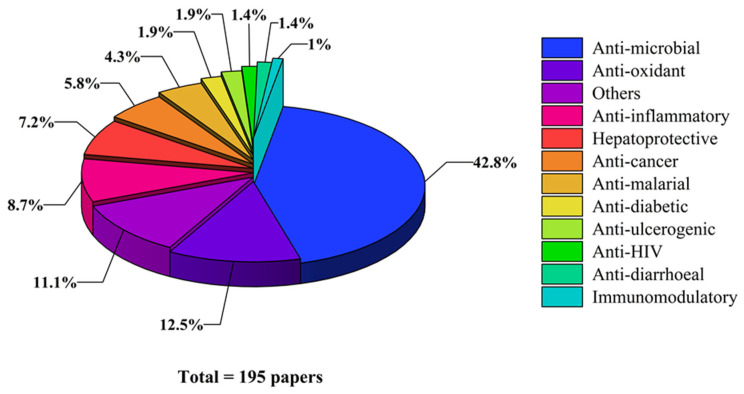
The percentage of each biological activity among the surveyed literature.

Extensive phytochemical studies have revealed that species within the genus *Alchornea* contain a rich and diverse range of primary and secondary metabolites. These include terpenoids, steroids, flavonoids, alkaloids, phenolic compounds, fatty acids, lignans, and tannins, all of which have significant medicinal uses [121]. Phytochemical research on this genus dates back to 1962, when Alves and Prista et al. first reported the isolation and characterization of gentisic acid (**228**) from *A. cordifolia*, thereby initiating scientific interest in the phytoconstituents of *Alchornea* species [122]. Phytochemical analysis of *Alchornea* species reveals a diverse profile of primary and secondary metabolites. Quantitative analyses of *A. cordifolia* leaf extracts show high levels of total flavonoids (26.12 mg QE/g), tannins (58.69 mg/g), and phenolics (62.24 mg GAE/g), consistent with significant antioxidant capacity and radical-scavenging activity. Essential oils from leaves and fruits contain methyl salicylate (25.3%), citronellol (21.4%), and *β*-caryophyllene (15.5%), along with minor constituents such as 1,8-cineole (5.5%), indicating mono- and sesquiterpenoid biosynthetic pathways linked to anti-inflammatory and antimicrobial effects. Seed oils comprise palmitic acid (8–15%), linoleic acid (8–12%), and a substantial fraction of epoxidized fatty acids (48–63%), highlighting the role of lipid metabolism. Alkaloids, including alchorneinone, alchorneine, isoalchorneine, and yohimbine, are present in small amounts but serve as chemotaxonomic and bioactive markers [123,124,125,126,127]. Metabolite characterization relies on GC-MS for volatiles and fatty acids, LC-MS and HPLC-DAD for polar compounds, and NMR spectroscopy coupled with high-resolution mass spectrometry for structural elucidation. To date, a total of **396** compounds have been identified from the different species of genus *Alchornea*, including terpenes and terpenoids (**1–171**), fatty acids (**172–183**), Alcohols, aldehydes, ketones, and esters (**184–213**), phenolic compounds (**214–246**), flavonoids (**247–304**), lignans and tannins (**305–332**), alkaloids (**333–373**), and other compounds (**374–396**). The names of compounds, species, parts of plants, and chemical structures are shown in Table 3 and Figure 3, Figure 4, Figure 5, Figure 6, Figure 7, Figure 8, Figure 9, Figure 10, Figure 11 and Figure 12. Terpenes and terpenoids are the most diverse class of compounds, present in seven species and exceptionally rich in *A. tiliifolia*, *A. cordifolia*, and *A. latifolia*. Volatile terpenoids are prevalent in the leaves and fruits of *A. tiliifolia*, *A. floribunda*, and *A. triplinervia*. Aliphatic hydrocarbons and fatty acids are relatively rare and mainly seed-specific, with *A. cordifolia* notable for its high proportion of epoxidized fatty acids; other species contain comparatively higher levels of linoleic and oleic acids. Phenolic compounds show strong lineage specificity, with *A. trewioides* and *A. davidii* emerging as phenolic-rich species, including lignins and tannins, mostly absent from most other species, while flavonoids are more generally distributed across many species, including *A. castaneifolia* and *A. floribunda*, particularly in leaves. Alkaloids display the most restricted distribution, accumulating mainly in stems and roots, with the highest diversity in *A. rugosa* and *A. glandulosa*. However, the flowers and fruits of *Alchornea* species have not been studied extensively to identify their phytochemical constituents, likely because they are not widely used in traditional medicinal practices. In contrast, leaves, stems, and bark are the most widely exploited plant parts in ethnomedicine for the isolation and identification of bioactive compounds.

The genus *Alchornea* is characterized by specialized nitrogen-based compounds that have attracted significant interest due to their limited natural occurrence and chemotaxonomic significance. Among these, uniquely structured alkaloids such as alchorneinone (**338**), isoalchorneine (**339**), alchornedine (**343**), alchorneine (**346**), alchorneinol (**347**), and yohimbine (**357**) serve as important chemotaxonomic markers for the genus *Alchornea*. These compounds are mainly found in the stems and roots of specific species, such as *A. floribunda* and *A. hirtella*, indicating a restricted distribution. This suggests the presence of specialized biosynthetic pathways possibly shared within the tribe Alchorneae, supporting the use of these compounds to differentiate members of this group from related taxa. Structure–activity relationship (SAR) study of selected chemical constituents of the genus *Alchornea* shows that subtle structural diversity strongly influences biological potency. Among flavonoid compounds (**261**, **262**, **264**, **273**) sharing a conserved benzopyranone scaffold, comparative assays indicate that C-3 glycosylation and 3-*O*-methylation consistently reduce antioxidant activity, as reflected by higher IC_50_ values, likely due to steric hindrance, decreased accessibility, and reduced planarity of phenolic hydroxyl groups essential for radical scavenging. In contrast, pentacyclic triterpenoid compounds (**135**, **136**, **151**, **152**) show that variations in skeleton (oleanane, lupane, and ursane) and functionalization at C-3 and C-28 modulate anti-inflammatory effects, with acetoxy or hydroxyl substituents enhancing effects through target interactions and improved hydrogen bonding. Ursane-type terpenoid compounds bearing a C-19 methylene group show superior potency, indicating target binding and optimized hydrophobic interactions. These findings reveal how minor substituent changes can translate into marked differences in bioactivity, providing a rational basis for selecting and optimizing leads from *Alchornea* species.

**Table 3:** Compounds isolated and identified from *Alchornea* species.

No.	Compounds	Parts of the Plant	Resources	References
** Terpenes and terpenoids **				
1	Sabinene	Leaf oil	*A. tiliifolia*	[128]
2	*α*-Thujene	Leaf oil	*A. tiliifolia*	[128]
3	*β*-Pinene	Leaf oil	*A. tiliifolia*	[128]
4	Camphene	Leaf oil	*A. tiliifolia*	[128]
5	L-Fenchone	Leaves	*A. cordifolia*	[129]
6	*α*-Pinene	Leaf oil, fruits	*A. tiliifolia*, *A. cordifolia*	[124,128]
7	*α*-Fenchol	Leaf oil	*A. tiliifolia*	[128]
8	Borneol	Leaf oil, leaves	*A. tiliifolia*, *A. cordifolia*	[128,129]
9	Myrtenol	Leaf oil	*A. tiliifolia*	[128]
10	Eucalyptol	Leaves	*A. cordifolia*	[129]
11	D-Limonene	Leaves	*A. cordifolia*	[129]
12	*α*-Phellandrene	Fruits	*A. cordifolia*	[124]
13	1-Methyl-4-(1-methylethyl)-cyclohexanol	Leaves	*A. cordifolia*	[130]
14	(-)-4-Terpineol	Leaves	*A. cordifolia*	[129]
15	Terpinen-4-ol	Leaf oil	*A. tiliifolia*	[128]
16	*α*-Terpineol	Leaf oil	*A. tiliifolia*	[128]
17	(*R*)-Limonene	Leaf oil	*A. tiliifolia*	[128]
18	Terpinolene	Leaf oil, fruits	*A. tiliifolia*, *A. cordifolia*	[124,128]
19	1,8-Cineole	Leaf oil, fruits	*A. tiliifolia*, *A. cordifolia*	[124,128]
20	*γ*-Terpinene	Leaf oil	*A. tiliifolia*	[128]
21	*α*-Terpinene	Leaf oil	*A. tiliifolia*	[128]
22	*ρ*-Cymene	Leaf oil, fruits	*A. tiliifolia*, *A. cordifolia*	[124,128]
23	Cyclopropanecarboxylic acid, 2,2-dimethyl-3-(2-methyl-1-propenyl)-, (4-methylphenyl) methyl ester	Roots	*A. laxiflora*	[131]
24	*β*-Elemene	Fruits	*A. cordifolia*	[124]
25	Myrcene	Leaf oil	*A. tiliifolia*	[128]
26	(*Z*)-*β*-Ocimene	Leaf oil	*A. tiliifolia*	[128]
27	(*E*)-*β*-Ocimene	Leaf oil	*A. tiliifolia*	[128]
28	Linalool	Leaf oil	*A. tiliifolia*	[128]
29	Neryl formate	Leaf oil	*A. tiliifolia*	[128]
30	Nerolidol	Leaf oil, fruits	*A. tiliifolia*, *A. cordifolia*	[124,128]
31	Farnesol	Leaf oil	*A. tiliifolia*	[128]
32	(*Z*)-Rose oxide	Fruits	*A. cordifolia*	[124]
33	*α*-Bisabolol	Fruits	*A. cordifolia*	[124]
34	*γ*-Curcumene	Leaf oil	*A. tiliifolia*	[128]
35	(*Z*)-*γ*-Bisabolene	Leaf oil	*A. tiliifolia*	[128]
36	(-)-*Trans*-*α*-bergamotene	Leaves	*A. cordifolia*	[129]
37	*α*-(*E*)-Ionone	Fruits	*A. cordifolia*	[124]
38	(*E*)-*β*-Damascenone	Fruits	*A. cordifolia*	[124]
39	2-Coumaranone	Leaves	*A. laxiflora*	[132]
40	Isololiolide	Leaves	*A. laxiflora*	[132]
41	Geosmin	Fruits	*A. cordifolia*	[124]
42	Bicyclogermacren	Fruits	*A. cordifolia*	[124]
43	Cadinol	Leaves	*A. cordifolia*	[129]
44	*β*-Selinene	Fruits	*A. cordifolia*	[124]
45	Cubenol	Leaves, fruits	*A. cordifolia*	[124,129]
46	10-*epi*-*γ*-Eudesmol	Fruits	*A. cordifolia*	[124]
47	*δ*-Cadinene	Leaf oil, fruits	*A. tiliifolia*, *A. cordifolia*	[124,128]
48	*γ*-Eudesmol	Leaf oil	*A. tiliifolia*	[128]
49	Bicyclogermacrene	Leaf oil	*A. tiliifolia*	[128]
50	*α*-Muurolene	Leaf oil	*A. tiliifolia*	[128]
51	Germacrene D	Leaf oil, fruits	*A. tiliifolia*, *A. cordifolia*	[124,128]
52	*α*-Copaene	Leaf oil, fruits	*A. tiliifolia*, *A. cordifolia*	[124,128]
53	*α*-Calacorene	Leaf oil, fruits	*A. tiliifolia*, *A. cordifolia*	[124,128]
54	*β*-Cubebene	Leaf oil	*A. tiliifolia*	[128]
55	*α*-Gurjunene	Fruits	*A. cordifolia*	[124]
56	Allo-aromadendrene	Leaf oil, fruits	*A. tiliifolia*, *A. cordifolia*	[124,128]
57	*β*-Gurjunene	Leaf oil	*A. tiliifolia*	[128]
58	*α*-Cedrene	Leaf oil	*A. tiliifolia*	[128]
59	1-(2,3,4,7,8,8a-hexahydro-3,6,8,8-tetramethyl-1h-3a,7-methanoazulen-5-yl)-ethanone	Leaves	*A. cordifolia*	[133]
60	*α*-Humulene	Leaf oil, fruits	*A. tiliifolia*, *A. cordifolia*	[124,128]
61	*β*-Caryophyllene	Leaf oil	*A. tiliifolia*	[128]
62	Caryophyllene oxide	Leaf oil, fruits	*A. tiliifolia*, *A. cordifolia*	[124,128]
63	(-)-(*E*)-Caryophyllene	Leaves, fruits	*A. cordifolia*	[124,129]
64	Eudenol	Leaves	*A. cordifolia*	[129]
65	Bisabolol oxide B	Leaf oil	*A. tiliifolia*	[128]
66	Linalool	Leaves	*A. cordifolia*	[129]
67	Citronellol	Fruits	*A. cordifolia*	[124]
68	Nerol	Fruits	*A. cordifolia*	[124]
69	Geraniol	Fruits	*A. cordifolia*	[124]
70	Iso-geraniol	Fruits	*A. cordifolia*	[124]
71	Geranial	Fruits	*A. cordifolia*	[124]
72	Neral	Fruits	*A. cordifolia*	[124]
73	2,6,10-Trimethylundecan-(5*E*)- 2,5,9-trien-4-one	Leaves	*A. laxiflora*	[132]
74	Neophytadiene	Leaves	*A. cordifolia*	[133]
75	(*E*/*E*)-*α*-Farnesene	Leaf oil, fruits	*A. tiliifolia*, *A. cordifolia*	[124,128]
76	(*E*)-*β*-Farnesene	Leaf oil	*A. tiliifolia*	[128]
77	(*Z*/*E*)-*α*-Farnesene	Leaf oil	*A. tiliifolia*	[128]
78	Farnesol	Leaves	*A. cordifolia*	[129]
79	Hexahydrofarnesyl acetone	Leaves	*A. laxiflora*	[134]
80	6,10,14-Trimethylpentadecan-2-one	Leaves	*A. cordifolia*	[133]
81	Heptadecane	Leaves	*A. cordifolia*	[130]
82	Nonadecane	Leaves	*A. cordifolia*	[130]
83	Nonacosane	Leaves	*A. cordifolia*	[129]
84	Hexatriacontane	Leaves	*A. cordifolia*	[133]
85	Tetracontane	Leaves	*A. cordifolia*	[133]
86	Tetratetracontane	Leaves	*A. cordifolia*	[130]
87	2-Methyloctacosane	Leaves	*A. cordifolia*	[130]
88	*Cis*-3-decene	Leaves	*A. cordifolia*	[130]
89	1-Tetradecene	Roots	*A. laxiflora*	[131]
90	1-Hexadecene	Leaves, roots	*A. cordifolia*, *A. laxiflora*	[131,133]
91	1-Octadecene	Leaves, roots	*A. cordifolia*, *A. laxiflora*	[132,133,134]
92	1-Nonadecene	Leaves	*A. cordifolia*	[133]
93	1,19-Eicosadiene	Leaves	*A. cordifolia*	[135]
94	1-Octadecyne	Leaves	*A. cordifolia*	[130]
95	1-Hexadecanol	Leaves	*A. laxiflora*	[132]
96	Octen-3-ol	Leaves	*A. cordifolia*	[124,129]
97	(*E*)-2-Hexenal	Fruits	*A. cordifolia*	[124]
98	Nonanal	Leaf oil	*A. tiliifolia*	[128]
99	8-Pentadecanone	Leaves	*A. cordifolia*	[133]
100	Dodecanoic acid, methyl ester	Leaves	*A. cordifolia*	[133]
101	Ethyl laurate	Roots	*A. laxiflora*	[131]
102	Ethyl myristate	Roots	*A. laxiflora*	[131]
103	Methyl isostearate	Leaves	*A. laxiflora*	[134]
104	Methyl linoleate	Leaves	*A. laxiflora*, *A. cordifolia*	[132,133]
105	Ethyl linoleate	Leaves	*A. laxiflora*	[134]
106	Methyl elaidolinolenate	Leaves	*A. laxiflora*, *A. cordifolia*	[132,133]
107	Methyl oleate	Roots	*A. laxiflora*	[131]
108	Propyl linoleate	Roots	*A. laxiflora*	[131]
109	Ethyl oleate	Roots	*A. laxiflora*	[131]
110	11-Tetradecyn-1-ol, 1-acetate	Leaves	*A. cordifolia*	[135]
111	Dimethyl undecanedioate	Leaves	*A. laxiflora*	[132]
112	2-Bromotetradecane	Leaves	*A. cordifolia*	[130]
113	3,7,11,15-Tetramethyl-2-hexadecenol	Leaves	*A. cordifolia*	[133]
114	Phytol	Leaves	*A. cordifolia*, *A. laxiflora*	[132,133,134]
115	Phytol acetate	Leaves	*A. cordifolia*	[133]
116	Squalene	Leaves, roots	*A. cordifolia*, *A. laxiflora*	[131,133]
117	Phytyl myristate	Leaves	*A. cordifolia*	[133]
118	Lycoxanthin	Leaves	*A. laxiflora*	[134]
119	Rhodopin	Leaves	*A. laxiflora*	[134]
120	Anhydrorhodovibrin	Leaves	*A. laxiflora*	[134]
121	Tetrahydrospirilloxanthin	Leaves	*A. laxiflora*	[134]
122	4,8,12,16-Tetra-methylheptadecan-4-olide	Leaves	*A. cordifolia*	[133]
123	*α*-Tocospiro B	Leaves	*A. cordifolia*	[133]
124	2,2,4-Trimethyl-3-(3,8,12,16- tetramethyl-heptadeca3,7,11,15- tetraenyl)-cyclohexanol	Leaves	*A. laxiflora*	[134]
125	Astaxanthin	Leaves	*A. laxiflora*	[134]
126	(1a*R*,4*S*,4a*S*,7*R*,7a*S*,7b*R*)-1,1,4,7-Tetramethyl-decahydro-1H-cyclopropa(e)azulene-4,7-diol	Leaves	*A. cordifolia*	[133]
127	17-Hydroxyingenol	Leaves	*A. laxiflora*	[109]
128	Byzantionoside B	Leaves	*A. laxiflora*	[136]
129	Trewiosides A	Branches, leaves	*A. trewioides*	[40]
130	Trewiosides B	Branches, leaves	*A. trewioides*	[40]
131	Alnamicosides B	Branches, leaves	*A. annamica*	[16]
132	Blumenol C glucoside	Branches, leaves	*A. annamica*	[16]
133	Alnamicosides A	Branches, leaves	*A. annamica*	[16]
134	Leeaoside	Leaves	*A. laxiflora*	[136]
135	Lupeol	Branches, leaves, twigs	*A. annamica*, *A. triplinervia*, *A. tiliifolia*	[16,137,138]
136	Betulin	Leaves	*A. laxiflora*	[134]
137	Seco-3,4-friedelin	Leaf	*A. latifolia*	[139]
138	Seco3,4-taraxerone	Leaf	*A. latifolia*	[139]
139	Taraxerone	Leaf	*A. latifolia*	[139]
140	Taraxerol	Leaf	*A. latifolia*	[139]
141	3*α*-Hydroxytaraxer-14-en-28-oic acid	Rhizomes	*A. trewioides*	[140]
142	3-*O*-Acetyl-erythrodiol	Stem	*A. cordifolia*	[141]
143	3-*O*-Acetyl-aleuritolic acid	Stem, roots, bark	*A. cordifolia*	[141,142]
144	3-Acetoxy-oleanoic acid	Rhizomes	*A. trewioides*	[140]
145	3*β*-Acetoxy-12-oleanen-11-one	Rhizomes	*A. trewioides*	[140]
146	*Epi*-friedelinol	Leaf	*A. latifolia*, *A. rugosa*, *A. triplinervia*	[120,137,139]
147	Friedelin	Leaf, stem	*A. latifolia*, *A. cordifolia*, *A. rugosa*, *A. triplinervia*	[120,137,139,141]
148	Friedelan-7*α*-ol	Leaves	*A. sidifolia*	[143]
149	Friedelane-3-one-28-al	Stem	*A. cordifolia*	[141]
150	Adipedatol	Leaves	*A. laxiflora*	[109]
151	3-Acetyl-oleanolic acid	Stem bark	*A. laxiflora*	[144]
152	3-Acetoxy-ursolic acid	Stem bark	*A. laxiflora*	[144]
153	Sugeroside	Branches, leaves	*A. trewioides*	[40]
154	Tetrahydrosmilagenin	Leaves	*A. cordifolia*	[130]
155	Campesterol	Leaves	*A. triplinervia*, *A. cordifolia*	[130,137]
156	*β*-Sitosterol	Branches, leaves	*A. annamica*, *A. glandulosa*, *A. sidifolia*, *A. triplinervia*, *A. cordifolia*, *A. tiliifolia*	[16,127,137,138,142,143,145,146,147]
157	*γ*-Sitosterol	Leaves	*A. cordifolia*	[133]
158	3*β*-Hydroxy-5*α*-stigmastane-24-ene	Leaves	*A. floribunda*	[91]
159	Stigmasterol	Leaves, stem	*A. glandulosa*, *A. triplinervia*, *A. cordifolia*	[130,133,137,141,145]
160	Ergosterol	Leaves	*A. cordifolia*	[130]
161	4-Vinylcholestan-3-ol	Leaves	*A. laxiflora*	[134]
162	Cholest-4-en-3-one	Roots	*A. laxiflora*	[131]
163	5*α*-Stigmastane-3,6-dione	Leaves	*A. floribunda*	[91]
164	5*α*-Stigmastane-23-ene-3,6-dione	Leaves	*A. floribunda*	[91]
165	Ethyl iso-allocholate	Leaves	*A. cordifolia*, *A. laxiflora*	[129,134]
166	24-Methylenecycloartanol	Leaves	*A. cordifolia*	[133]
167	4-(Acetyloxy)-1-(1,5-dimethylhexyl)-3*α*,6,6,12*α*-tetramethyltetradecahydro-1H-cyclopenta[a]cyclopropa[e]phenanthren-7-yl acetate	Leaves	*A. cordifolia*	[133]
168	9*β*,11*β*-Epoxy-hydrocortisone	Leaves	*A. cordifolia*	[130]
169	3-Acetoxy-7,8-epoxylanostan-11-ol	Leaves	*A. cordifolia*, *A. laxiflora*	[129,134]
170	Daucosterol	Branches, leaves, and stem bark	*A. annamica*, *A. cordifolia*, *A. laxiflora*, *A. trewioides*, *A. tiliifolia*	[16,136,138,142,144,148]
171	20*α*-Hydroxypregn-4-en-3-one *β*-D-glucopyranoside	Branches, leaves	*A. trewioides*	[40]
** Fatty acids **				
172	Palmitic acid	Leaves	*A. laxiflora*	[132,149]
173	*n*-Hexadecanoic acid	Leaves	*A. cordifolia*	[133]
174	Stearic acid	Leaves, roots	*A. laxiflora*	[131,132]
175	*α*-Linoleic acid	Roots	*A. laxiflora*	[131]
176	Petroselinic acid	Leaves	*A. laxiflora*	[149]
177	7-Hexadecenoic acid	Leaves	*A. cordifolia*	[130]
178	Oleic acid	Leaves	*A. cordifolia*, *A. laxiflora*	[129,135,149]
179	2-Hydroxystearic acid	Leaves	*A. cordifolia*	[127]
180	Deepoxyalchornoic acid	Ripe fruits	*A. cordifolia*	[150]
181	Alchornoic acid	Ripe fruits	*A. cordifolia*	[150]
182	(+) *Cis*-14,15-epoxy-*cis*-11-eicosenoic Acid	Seed oil	*A. cordifolia*	[151]
183	9,10,12,13-Tetrabromooctadecanoic acid	Leaves	*A. cordifolia*	[133]
** Alcohols, aldehydes, ketones, and esters **				
184	1-Tetradecanol	Leaves	*A. laxiflora*	[132]
185	1-Eicosanol	Leaves	*A. cordifolia*	[133]
186	1-Heptacosanol	Leaves, roots	*A. laxiflora*	[131,134]
187	1-Heptatriacotanol	Leaves	*A. laxiflora*	[134]
188	1,30-Triacontanediol	Leaves	*A. cordifolia*	[135]
189	8,8-Dimethoxy-2-octanol	Leaves	*A. cordifolia*	[130]
190	*Z*, *E*-2,13-Octadecadien-1-ol	Leaves	*A. laxiflora*	[132]
191	(9*Z*)-1-(2-[(9*Z*)-9-Octadecenyloxy] ethoxy)-9-octadecene	Leaves	*A. laxiflora*	[134]
192	1,2-Octadecylene oxide	Leaves	*A. cordifolia*	[135]
193	2-((9Z,12Z)-Octadeca-9,12-dienyloxy) ethanol	Leaves	*A. laxiflora*	[134]
194	3-(Octadecycloxy) propyl oleate	Leaves	*A. cordifolia*	[129]
195	Hexadecanal	Leaves	*A. cordifolia*	[135]
196	Octadecanal	Leaves	*A. cordifolia*	[135]
197	Henicosyl formate	Leaves	*A. laxiflora*	[132]
198	2-Hydroxyethyl oleate	Leaves	*A. laxiflora*	[132]
199	(10*Z*)-Tetradec-10-enoic acid-(2*S*)- 2-carboxy-2-hydroxyethyl ester	Stem barks	*A. laxiflora*	[144]
200	2,3-Dihydroxypropyl octacosanoate	Ripe fruits	*A. cordifolia*	[150]
201	Bisalchornoicester	Ripe fruits	*A. cordifolia*	[150]
202	Methyl palmitate	Leaves, roots	*A. laxiflora*, *A. cordifolia*	[131,132,133,134]
203	Ethyl palmitate	Leaves, roots	*A. laxiflora*	[131,134]
204	Ethyl stearate	Roots	*A. laxiflora*	[131]
205	Ethyl tetracosanoate	Roots	*A. laxiflora*	[131]
206	(14*Z*)-14-Octadecen-1-yl acetate	Leaves	*A. cordifolia*	[135]
207	Icosyl oleate	Leaves	*A. laxiflora*	[134]
208	Oleyl palmitoleate	Leaves	*A. laxiflora*	[134]
209	Cyclopropanedodecanoic acid	Leaves	*A. laxiflora*	[134]
210	Methyl acetyl ricinoleate	Leaves	*A. laxiflora*	[132]
211	1,3-Diacetyloxypropan-2-yl icosanoate	Leaves	*A. laxiflora*	[132]
212	Ether 3-Ethyl-5-(2-ethylbutyl)- octadecane	Leaves	*A. laxiflora*	[134]
213	1-Bromooctadecane	Roots	*A. laxiflora*	[131]
** Phenolics **				
214	4-Vinylphenol	Leaves	*A. laxiflora*	[132]
215	2-Methoxy-4-vinylphenol	Leaves	*A. laxiflora*	[132]
216	2,6-Dimethoxyquinone	Rhizomes	*A. trewioides*	[140]
217	Butylated hydroxyanisole	Leaves	*A. laxiflora*	[132]
218	Phenol, 2,6-Bis(1,1-dimethylethyl)	Leaves	*A. cordifolia*	[130]
219	Phenol, 2,4-bis-(1,1-dimethylethyl), TMS	Leaves	*A. laxiflora*	[134]
220	Phenol, 2,6-bis(1,1-dimethylethyl) methyl	Leaves	*A. laxiflora*	[134]
221	Pyrogallol	Leaves	*A. laxiflora*	[132]
222	*ρ*-Hydroxybenzoic acid	Leaves	*A. glandulosa*, *A. trewioides*	[41,152]
223	Salicylic acid	Leaves	*A. trewioides*	[41]
224	Methyl salicylate	Fruits	*A. cordifolia*	[124]
225	Protocatechuic acid	Leaves	*A. cordifolia*	[153]
226	3,4-Dihydroxybenzoic acid	Leaves	*A. trewioides*, *A. cordifolia*	[44,127]
227	2,5-Dihydroxybenzoic acid	Leaves	*A. trewioides*	[44]
228	Gentisic acid	Bark, roots	*A. cordifolia*	[122]
229	Shikimic acid	Leaves, ripe fruits	*A. castaneifolia*, *A. cordifolia*	[51,127,150]
230	Gallic acid	Branches, leaves, twigs, roots, bark, ripe fruits	*A. annamica*, *A. castaneifolia*, *A. davidii*, *A. glandulosa*, *A. trewioides*, *A. triplinervia*, *A. cordifolia*, *A. rugosa; A. tiliifolia*	[16,44,51,52,87,127,137,138,146,150,153,154,155,156]
231	Methyl gallate	Branches, leaves, bark, and stem	*A. annamica*, *A. davidii*, *A. glandulosa*, *A. trewioides*, *A. triplinervia*, *A. castaneifolia*, *A. cordifolia*, *A. rugosa*, *A. tiliifolia*	[16,43,52,87,137,138,141,146,156]
232	Ethyl gallate	Leaves, roots	*A. glandulosa*, *A. trewioides*	[148,154,155]
233	4-*O*-Methylgallic acid	Ripe fruits	*A. cordifolia*	[150]
234	Methyl syringate	Leaves	*A. rugosa*	[120]
235	Syringic acid	Roots	*A. trewioides*	[155]
236	1-*O*-Galloyl-*β*-D-glucose	Leaves	*A. trewioides*	[41]
237	Gluco-syringic acid	Roots	*A. trewioides*	[155]
238	Erigeside C	Roots	*A. trewioides*, *A. rugosa*	[155,156]
239	*cis*-*ρ*-Coumaric acid	Leaves	*A. trewioides*	[41]
240	*trans*-*ρ*-Coumaric acid	Leaves	*A. trewioides*	[41]
241	Caffeic acid	Leaves	*A. trewioides*	[41]
242	Caffeic acid methyl ester	Leaves	*A. trewioides*	[41]
243	Coniferyl alcohol	Leaves	*A. laxiflora*	[132]
244	4-*O*-Galloylquinic acid	Leaves	*A. trewioides*	[41]
245	5-*O*-Galloylquinic acid	Leaves	*A. trewioides*	[41]
246	1,6-Bis-*O*-galloyl-*β*-D-glucose	Leaves, roots	*A. trewioides*	[41,155]
** Flavonoids **				
247	Naringenin	Leaves	*A. cordifolia*	[127]
248	Kaempferol	Branches, leaves	*A. annamica*, *A. cordifolia*	[16,127,157]
249	Kaempferol-3-*O*-*α*-L-rhamnopyranoside	Leaves	*A. floribunda*, *A. glandulosa*	[90,154]
250	Kaempferol-3-*O*-galactoside	Ripe fruits	*A. cordifolia*, *A. tiliifolia*	[138,150]
251	Kaempferol-7-*O*-glucoside	Leaves	*A. cordifolia*	[127]
252	(-)-Taxifolin	Leaves	*A. cordifolia*	[127]
253	2*R*,3*R*-Dihydroquercetin-3-*O*-*β*-D-galactopyranoside	Leaves	*A. floribunda*	[158]
254	2*R*,3*R*-dihydroquercetin-3-*O*-*α*-L-arabinopyranoside	Leaves	*A. floribunda*	[158]
255	(+)-Taxifolin	Leaves	*A. floribunda*, *A. rugosa*	[31,156]
256	Taxifolin-3-*O*-*β*-D-galactopyranoside	Leaves	*A. laxiflora*, *A. rugosa*	[136,156]
257	Taxifolin-3-*O*-*β*-D-xylopyranoside	Leaves	*A. laxiflora*	[136]
258	Taxifolin-*O*-hexoside	Leaves	*A. cordifolia*	[127]
259	Epicatechin	Leaves	*A. castaneifolia*, *A. floribunda*	[31,51]
260	(-)-Catechin	Leaves	*A. floribunda*	[31]
261	Quercetin	Branches, leaves, bark	*A. annamica*, *A. glandulosa*, *A. trewioides*, *A. triplinervia*, *A. cordifolia*, *A. laxiflora*, *A. rugosa*	[16,42,52,104,127,137,146,153,156,157,159,160]
262	Quercetin-3-*O*-*β*-D-glucopyranoside	Leaves	*A. floribunda*, *A. triplinervia*, *A. trewioides*, *A. cordifolia*, *A. laxiflora*	[42,127,136,137,153,158,160,161]
263	Avicularin	Leaves	*A. cordifolia*	[127]
264	Rutin	Leaves	*A. trewioides*, *A. cordifolia*, *A. laxiflora*, *A. rugo*	[42,127,156,157,162]
265	Quercetin-*7*-*O*-*β*-D-glucopyranoside	Leaves	*A. triplinervia*	[137]
266	Quercetin-3-*O*-*α*-L-rhamnopyranoside	Leaves	*A. floribunda*, *A. cordifolia*, *A. laxiflora*, *A. trewioides*, *A. glandulosa*	[42,106,154,158,159,160,162,163]
267	Quercetin-7-rhamnoside	Leaves	*A. laxiflora*	[159]
268	Quercetin-3-*O*-*β*-D-galactopyranoside	Leaves	*A. glandulosa*, *A. triplinervia*, *A. castaneifolia*, *A. cordifolia*	[52,127,137,146]
269	Quercetin-3-*O*-*β*-D-arabinopyranoside	Leaves	*A. floribunda*, *A. castaneifolia*, *A. cordifolia*, *A. triplinervia*, *A. glandulosa*	[52,127,137,146,153,158]
270	Quercetin-*O*-hexosyl hexoside	Leaves	*A. cordifolia*	[127]
271	Quercetin-3-*O*-gallate	Leaves	*A. glandulosa*	[98]
272	Reynoutrin	Leaves	*A. laxiflora*	[136]
273	Isorhamnetin-3-*O*-*β*-D-xyloside	Leaves, twigs	*A. davidii*	[46]
274	Quercetin-3,4′-diacetate	Leaves	*A. laxiflora*	[162]
275	Quercetin-7,4′-disulphate	Leaves	*A. laxiflora*	[104,160]
276	Quercetin-3′,4′-disulphate	Leaves	*A. laxiflora*	[104,160]
277	Quercetin-3,7,3′,4′-tetrasulphate	Leaves	*A. laxiflora*	[160]
278	5′-methyl 4′,3,5,7- tetrahydroxy anthocyanidines	Leaves	*A. cordifolia*	[164]
279	5′-methyl 4′,4′-propenoxy anthocyanidine 7-*O*-*β*-D-diglucopyranoside	Leaves	*A. cordifolia*	[164]
280	Apigenin	Leaves	*A. cordifolia*	[127]
281	Chrysoeriol-*O*-hexoside	Leaves	*A. cordifolia*	[127]
282	Isovitexin	Leaves	*A. rugosa*, *A. cordifolia*, *A. trewioides*	[42,120,127,148]
283	Isovitexin-2′′-*O*-rhamnoside	Leaves	*A. cordifolia*	[127]
284	Cosmosiin	Leaves	*A. cordifolia*	[127]
285	Apigenin-8-C-*β*-D-glucopyranoside	Leaves	*A. trewioides*, *A. cordifolia*	[42,127]
286	Apigenin-8-C-(*α*-L-rhamnopyranosyl-(1→2)-*β*-D-glucopyranoside)	Leaves, twigs	*A. coelophylla A. cordifolia*	[88,127]
287	Vicenin-2	Leaves	*A. cordifolia*	[127]
288	Luteolin	Leaves	*A. cordifolia; A. rugosa*	[127,156]
289	Luteolin-7-*O*-glucoside	Leaves	*A. cordifolia*	[127]
290	Tricin-7-*O*-glucoside	Leaves	*A. cordifolia*	[127]
291	Tricin	Leaves	*A. cordifolia*	[127]
292	Chrysoeriol	Leaves	*A. cordifolia*	[127]
293	Myricetin	Leaves	*A. cordifolia*	[127]
294	Myricetin 3-*O*-glucoside	Leaves	*A. castaneifolia*, *A. cordifolia*	[51,161]
295	Myricetin-3-*O*-galactoside	Leaves	*A. castaneifolia*, *A. cordifolia*	[51,161]
296	Myricetin-3-*O*-*a*-L-rhamnoside	Leaves	*A. glandulosa*, *A. cordifolia*	[127,146,154,161]
297	Myricetin-3-arabinopyranoside	Leaves, bark	*A. castaneifolia*	[52]
298	Myricetin-3′-*O*-glucoside	Leaves	*A. cordifolia*	[127]
299	Myricetin-*O*-galloylrhamnoside	Leaves	*A. cordifolia*	[127]
300	Amentoflavone	Leaves, bark	*A. glandulosa*, *A. triplinervia*, *A. castaneifolia*	[52,137,146]
301	Proanthocyanidins A_1_	Leaves	*A. castaneifolia*	[51]
302	Procyanidin A_1_	Leaves	*A. cordifolia*	[127]
303	Proanthocyanidins A_2_	Leaves	*A. castaneifolia*	[51]
304	Procyanidin A_2_	Leaves	*A. cordifolia*	[127]
** Lignans and tannins **				
305	Pinoresinol	Leaves and twigs	*A. davidii*	[87]
306	(-)-Pinoresinol monomethyl ether	Leaves and twigs	*A. davidii*	[87]
307	(+)-syringaresinol	Leaves and twigs	*A. davidii*	[87]
308	Graminone A	Leaves and twigs	*A. davidii*	[87]
309	Boehmenan	Leaves and twigs	*A. davidii*	[87]
310	Schizandriside	Branches, leaves	*A. trewioides*	[40]
311	Syringaresinol-*β*-D-glucoside	Leaves	*A. laxiflora*	[136]
312	Decarboxy-ellagic acid	Leaves	*A. trewioides*	[43]
313	Ellagic acid	Leaves, stem bark	*A. trewioides*, *A. cordifolia*, *A. laxiflora*	[43,144,153,161,165]
314	3-*O*-Methylellagic acid	Leaves, stem bark	*A. trewioides*, *A. cordifolia*, *A. laxiflora*	[43,127,144]
315	3,3′-Di-*O*-methylellagic acid	Leaves	*A. cordifolia*	[127]
316	3,3′,4-Tri-*O*-methylellagic acid	Leaves, stem bark	*A. cordifolia*, *A. laxiflora*	[127,166]
317	Ellagic acid-4-*O*-glucoside	Leaves	*A. cordifolia*	[127]
318	Ellagic acid-*O*-pentoside	Leaves	*A. cordifolia*	[127]
319	3-*O*-Methylellagic acid-3-*O*-*α*-rhamnopyranoside	Stem bark	*A. laxiflora*	[144]
320	3,3′-Di-*O*-methylellagic acid 4-*O*-*α*-L-arabino- furanoside	Leaves and twigs	*A. davidii*	[87]
321	3,3′-Di-*O*-methylellagic acid 4-*O*-(5′-*O-*acetyl)-*α*-L-arabinofuranoside	Leaves and twigs	*A. davidii*	[87]
322	Valoneic acid dilactone	Leaves	*A. cordifolia*	[127]
323	Sanguisorbic acid dilactone	Leaves	*A. cordifolia*	[127]
324	1-*O*-Galloyl-6-*O*-luteoyl-*β*-D-glucopyranoside	Leaves	*A. cordifolia*	[161]
325	Corilagin	Leaves	*A. glandulosa*, *A. cordifolia*, *A. trewioides*	[43,98,127]
326	Tellimagrandin I	Leaves	*A. cordifolia*	[127]
327	Potentillin	Leaves	*A. cordifolia*	[127]
328	Mallotusinin	Leaves	*A. cordifolia*	[127]
329	Phyllanthusiin D	Leaves	*A. trewioides*	[43]
330	Geraniin	Leaves	*A. trewioides*	[43]
331	1-*O*-galloyl-3,6-(*R*)-HHDP-4-*O*-(1′-methoxy-4′-dehydrochebuloyl)-*β*-D-glucose	Leaves	*A. castaneifolia*	[51]
332	Tris-galloyl-HHDP-glucose	Leaves	*A. glandulosa*	[98]
** Alkaloids **				
333	Galegine	Leaves	*A. cordifolia*, *A. rugosa*	[39,127]
334	Pteroginidine	Leaves, roots, and bark	*A. glandulosa*, *A. hirtella*, *A. cordifolia*	[97,100,127,142,167]
335	Pterogynidine	Leaves, roots, and bark	*glandulosa*, *A. hirtella A. glandulosa*	[97,100,146,168]
336	*N*1, *N*2, *N*3-Triisopentenyl guanidine	Leaves, roots, and bark	*A. glandulosa*, *A. hirtella*, *A. cordifolia*	[100,127,142,167,169]
337	2-Amino-4-chloromethyl-thiazole	Leaves	*A. cordifolia*	[130]
338	Alchorneinone	Roots, roots, bark	*A. floribunda*, *A. hirtella*	[100,170]
339	Isoalchorneine	Roots, roots, bark	*A. floribunda*, *A. hirtella*	[100,170]
340	Uracil	Whole plant	*A. glandulosa*	[152]
341	*N*-(2-Phenylethyl) acetamide	Whole plant	*A. glandulosa*	[152]
342	*N*-(4-Hydroxy-phenyl ethyl) acetamide	Whole plant	*A. glandulosa*	[152]
343	Alchornedine	Leaves	*A. glandulosa*	[97]
344	1,3,7,9-tetramethyluric acid	Leaves	*A. laxiflora*	[132]
345	Cyclo(L-Pro-L-Val)	Whole plant	*A. glandulosa*	[152]
346	Alchorneine	Stem bark, root bark, leaves	*A. floribunda*, *A. hirtella*, *A. cordifolia*	[100,153,170]
347	Alchorneinol	Roots bark	*A. hirtella*	[100]
348	Cyclo(L-Pro-L-Ile)	Whole plant	*A. glandulosa*	[152]
349	Cyclo(L-Pro-L-Phe)	Whole plant	*A. glandulosa*	[152]
350	Cyclo(L-Pro-L-Tyr)	Whole plant	*A. glandulosa*	[152]
351	Indomethacin	Roots bark	*A. cordifolia*	[142]
352	4-Fluoro-2-nitroaniline, 5-[4-(pyrrolidin-1-yl) carbonylmethylpiperazin1-yl]	Leaves	*A. laxiflora*	[134]
353	Glycocholic acid	Leaves	*A. laxiflora*	[134]
354	Capsaicin	Roots	*A. laxiflora*	[131]
355	Dihydrocapsaicin	Roots	*A. laxiflora*	[131]
356	(2*R*)-2-Hydroxy-*N*- [(2*S*,3*S*,4*R*,15*Z*)-1,3,4-trihydroxy15-triaconten-2-yl] octacosamide	Stem bark	*A. laxiflora*	[144]
357	Yohimbine	Roots, roots, bark	*A. floribunda*, *A. hirtella*, *A. cordifolia*	[100,122,171]
358	Rugonidine E	Leaves	*A. rugosa*	[172]
359	Rugonidine F	Leaves	*A. rugosa*	[172]
360	Rugonidine C	Leaves	*A. rugosa*	[172]
361	Rugonidine D	Leaves	*A. rugosa*	[172]
362	Rugonidine A	Leaves	*A. rugosa*	[172]
363	Rugonidine B	Leaves	*A. rugosa*	[172]
364	Rugonines H	Leaves	*A. rugosa*	[39]
365	Rugonines E	Leaves	*A. rugosa*	[39]
366	Rugonines D	Leaves	*A. rugosa*	[39]
367	Alchornealaxine	Leaves	*A. laxiflora*	[136]
368	Rugonines F	Leaves	*A. rugosa*	[39]
369	Rugonines G	Leaves	*A. rugosa*	[39]
370	Rugonines A	Leaves	*A. rugosa*	[39]
371	Rugonines B	Leaves	*A. rugosa*	[39]
372	Rugonines C	Leaves	*A. rugosa*	[39]
373	Pheophorbide A	Leaves	*A. laxiflora*	[109]
** Others **				
374	2-Methyl-2-cyclopenten-1-one	Leaves	*A. laxiflora*	[132]
375	3-Hydroxy-4,4-dimethyldihydro-2(3H)-furanone	Leaves	*A. laxiflora*	[132]
376	3-Butyl-1,2,4-cyclopentanetrione	Roots	*A. laxiflora*	[131]
377	1,5-Anhydro-2,3,4,6-tetra-*O*-methyl-D-galactitol	Leaves	*A. laxiflora*	[132]
378	3-Methoxy-3-methyl-tetrahydropyran-2-one	Leaves	*A. cordifolia*	[130]
379	1,4-Dimethoxy-2-methyl cyclohexane	Leaves	*A. cordifolia*	[130]
380	1,5-Diisopropyl-2,3-dimethyl cyclohexane	Leaves	*A. cordifolia*	[130]
381	4-Hydroxy-6-pentyl-2H-pyran-2-one	Roots	*A. laxiflora*	[131]
382	Anthranilic acid	Bark, roots	*A. cordifolia*	[122]
383	Phthalic acid	Leaves	*A. laxiflora*	[132]
384	(*E*)-2-Hexenyl benzoate	Fruits	*A. cordifolia*	[124]
385	5,6-Dihydro-6-pentyl-2H-pyran-2-one	Roots	*A. laxiflora*	[131]
386	7-isopropyl-1,4-dimethyl-2,3-dihydro-1H-azulen-6-one	Rhizomes	*A. trewioides*	[140]
387	5-Benzofuranacetic acid	Leaves	*A. cordifolia*	[130]
388	Bicyclo [4.4.0]dec-2-ene-4-ol	Leaves	*A. cordifolia*	[130]
389	*α*-Benzyl benzoate	Fruits	*A. cordifolia*	[124]
390	1,1′-Biphenyl-3,4,4′-trimethoxy-6′-formyl	Roots	*A. laxiflora*	[131]
391	*m*-Diphenyloxy-benzene	Leaves	*A. cordifolia*	[164]
392	5,5′-Dibutoxy-2,2′-bifuran	Rhizomes	*A. trewioides*	[140]
393	Zeranol	Roots	*A. laxiflora*	[131]
394	1,2-Benzenedicarboxylic acid, bis(2-methylpropyl) ester	Leaves	*A. cordifolia*	[133]
395	2,3-Dimethoxy-10,11-dihydrodibenzo[b,f]oxepin-10-ol	Leaves	*A. laxiflora*	[132]
396	[(1*E*)-2-Ethyl-4-methyl-1,3-pentadienyl]benzene	Roots	*A. laxiflora*	[131]

### 4.1. Terpenes and Terpenoids

Terpenes and terpenoids are essential for plant physiology and defense and are well known for their medicinal effects. The occurrence of secondary metabolites such as friedelin (**147**), taraxerol (**140**), taraxerone (**139**), and campesterol (**155**) reflects distinct biosynthetic pathways. Their abundance and presence provide valuable chemotaxonomic markers and indicate taxon-specific metabolic specialism. Additionally, these compounds exhibit notable pharmacological effects, including antimicrobial and anti-inflammatory activities, underscoring their relevance in natural product–based drug discovery. To date, a total of **171** terpenoid compounds (**1–171**) have been isolated and identified from different *Alchornea* species, particularly *A. annamica*, *A. cordifolia*, *A. floribunda*, *A. glandulosa*, *A. latifolia*, *A. sidifolia*, *A. rugosa*, *A. triplinervia*, and *A. trewioides* [16,40,120,137,139,141,143,144], as shown in Table 3 and Figure 4.

### 4.2. Fatty Acids

Fatty acids are essential components of plant lipids. Fatty acids form the fundamental building blocks of lipids, such as triglycerides and phospholipids. The rare or structurally distinctive secondary metabolites, such as deepoxyalchornoic acid (**180**) and alchornoic acid (**181**), are of specific attention due to their unique chemical structures and limited taxonomic distribution, making them significant for chemotaxonomic diversity. Their occurrence can inform phylogenetic relationships and highlight potential leads for pharmacological study, showing their importance in taxon-based evaluation. Within the genus *Alchornea*, a total of **12** compounds (**172–183**) have been isolated and identified mainly from *A. cordifolia*, *A. laxiflora*, and *A. tiliifolia* [128,131,133], as illustrated in Table 3 and Figure 5.

### 4.3. Alcohols, Aldehydes, Ketones, and Esters

Plants produce a wide range of secondary metabolites that support physiological regulation, ecological adaptation, and interspecies interactions. Among these, alcohols, aldehydes, ketones, and esters are functionally significant and structurally diverse, often occurring as volatile or semi-volatile compounds involved in signaling and defense. Within the genus *Alchornea*, a total of **30** compounds (**184–213**) have been isolated and identified mainly from *A. laxiflora*, *A. cordifolia*, and *A. tiliifolia* [128,131,133]. The GC-MS analysis of the *n*-hexane fractions from the leaf extracts of *A. laxiflora*, *A. cordifolia*, and *A. floribunda* has shown the presence of Alcohols, aldehydes, ketones, and esters, which are recognized for their potential medicinal and nutritional properties [129,149], as illustrated in Table 3 and Figure 6.

### 4.4. Phenolic Compounds

Phenolic compounds are a class of naturally occurring organic compounds known for their anti-inflammatory, antimicrobial, antioxidant, and cytoprotective effects. Their presence supports the traditional medicinal uses of these species. Phenolic acids, such as shikimic (**229**), salicylic (**223**), gentisic (**228**), syringic (**235**), gallic (**230**), and caffeic acids (**241**), reproduce the shikimate and phenylpropanoid pathways, linking primary to secondary metabolite production. Shikimic acid serves as a key intermediate, while differences in hydroxylation and methylation provide chemotaxonomic insight. Additionally, from a taxonomic perspective, these compounds exhibit antioxidant, anti-inflammatory, and antimicrobial activities, showing their pharmacological effects. Phytochemical studies of *Alchornea* species have revealed a diverse range of phenolic compounds, with **33** chemical constituents (**214–246**) identified to date, as listed in Table 3 and Figure 7. These compounds have been isolated from various species, including *A. annamica*, *A. cordifolia*, *A. castaneifolia*, *A. davidii*, *A. glandulosa*, *A. trewioides*, *A. laxiflora*, and *A. triplinervia* [16,41,52,87,132,137,141,146]. Among these, gentisic acid (**228**) illustrates one of the earliest phenolic compounds isolated from *A. cordifolia*, first described by Alves and Prista in 1962.

### 4.5. Flavonoids

Flavonoids are a significant class of chemical constituents commonly studied for their wide-ranging medicinal uses, including anti-inflammatory, antioxidant, antiviral, antimicrobial, and anti-cancer effects. Flavonoids, including naringenin (**247**), kaempferol (**248**), quercetin (**261**), rutin (**264**), and luteolin (**288**), in both aglycones and glycosylated derivatives, are essential for phytochemical differentiation. The occurrence of these abovementioned compounds reveals glycosylation patterns that may reflect taxon-specific enzymatic processes, providing chemotaxonomic insight. Variations in substitution and glycosylation profiles can differentiate closely related species, while these compounds also exhibit anti-inflammatory and antioxidant effects, highlighting their pharmacological relevance. In the context of the genus *Alchornea*, a total of **58** compounds (**247–304**) have been isolated and identified from various species, particularly *A. annamica*, *A. cordifolia*, *A. glandulosa*, *A. laxiflora*, *A. trewioides*, and *A. triplinervia* [16,42,52,104,127,137,146,153,157,159,160], as presented in Table 3 and Figure 8.

### 4.6. Lignans and Tannins

Lignans and tannins are naturally occurring organic compounds primarily found in the leaves, bark, and seeds of plants. These compounds play essential roles in maintaining structural integrity and plant defense, as well as offering a diverse range of pharmacological effects, including anti-inflammatory, antimicrobial, and antioxidant properties. Their presence enhances the ethnopharmacological significance of *Alchornea* species and underscores their potential as a natural source for medicinal purposes. Phytochemical study of *Alchornea* species has shown the presence of lignans and tannins, with a total of **28** compounds (**305–332**) (Table 3 and Figure 9) isolated from species such as *A. castaneifolia*, *A. cordifolia*, *A. davidii*, *A. glandulosa*, *A. laxiflora*, *and A. trewioides* [43,51,87,98,127,136].

### 4.7. Alkaloids

Alkaloids are a prominent class of nitrogen-containing chemical constituents known for their broad spectrum of therapeutic uses, including antimicrobial, analgesic, anti-inflammatory, anti-cancer, and antimalarial effects. The structurally distinctive alkaloids, including alchorneinone (**338**), isoalchorneine (**339**), alchornedine (**343**), alchorneine (**346**), alchorneinol (**347**), and yohimbine (**357**), with complex structures and restricted taxonomic distributions, are valuable chemotaxonomic markers for the genus *Alchornea*. Their co-occurrence suggests specialized biosynthetic pathways from shared amino acid precursors. Many exhibit significant pharmacological effects, highlighting their potential as leads for biological and pharmacodynamic studies. Within the genus *Alchornea*, a total of **41** alkaloids (**333–373**) have been isolated, as shown in Table 3 and Figure 10. Their presence is relevant to the traditional therapeutic applications of these species and to their probable role as lead compounds in drug discovery. These alkaloids have been reported from different *Alchornea* species, notably *A. cordifolia*, *A. castaneifolia*, *A. floribunda*, *A. laxiflora*, *A. glandulosa*, *A. rugosa*, and *A. hirtella* [51,132,154,161,168,170,172].

### 4.8. Other Classes of Compounds

In addition to the major classes of chemical constituents previously outlined, **23** miscellaneous secondary metabolites (**374–396**) have been isolated and identified from species within the genus *Alchornea*, such as *A. cordifolia*, *A. laxiflora*, and *A. trewioides* [130,132,140], as shown in Table 3 and Figure 11.

## 5. Pharmacological Potential

The genus *Alchornea* includes over 50 species, and only 17 have been documented for traditional uses, phytochemistry, pharmacology, and toxicology studies. The unstudied species remain largely unknown: they lack both peer-reviewed documentation of their traditional use and modern phytochemical profiles. These species represent silent repositories of potential pharmacopeia, marginalized by the knowledge systems intended to explore them.

In the ongoing search for new drugs, natural products have consistently outperformed combinatorial chemistry in yielding structurally diverse bioactive lead compounds. Plant-derived extracts, fractions, and pure compounds continue to serve as a starting point for the discovery of new bioactive agents targeting a wide range of diseases affecting both humans and animals [12]. The documented traditional uses of *Alchornea* species offer a valuable foundation and scientific direction for modern pharmacological research. Extracts and pure compounds from various *Alchornea* species have exhibited a wide range of pharmacological activities, including antimicrobial, anti-inflammatory, hepatoprotective, anti-cancer, antimalarial, antioxidant, immunomodulatory, antidiabetic, anti-ulcerogenic, antidiarrheal, and anti-HIV effects.

The anti-inflammatory effects of *A. laxiflora* and *A. cordifolia* are well-documented in the literature. Mechanistically, these effects have been linked to the inhibition of nitric oxide (NO) production and 15-lipoxygenase (15-LOX) activity. Recent evidence further suggests that these activities may be mediated through the modulation of key intracellular signaling pathways, including the PI3K-Akt and MAPK cascades, which play central roles in regulating inflammatory gene expression. Despite this progress, the current evidence for anti-inflammatory activity remains preliminary, as most studies have used crude extracts and specific molecular-target data for isolated alkaloids are still lacking.

Among all biological activities reported for the genus *Alchornea*, antimicrobial activity is the most frequently documented, accounting for 42.8% of the surveyed literature. While many studies mainly report in vitro minimum inhibitory concentration (MIC) values, strong evidence comes from research involving pure compounds. For example, quercetin (**261**) and alchorneinone (**338**) have shown MIC values comparable to those of standard reference drugs. Additionally, beyond in vitro antibacterial tests, the ethyl acetate fractions of *A. laxiflora* have demonstrated potent in vivo antimalarial effects against *P. berghei*, supporting the traditional use of this species to treat fevers. Regarding anti-cancer activity, current evidence is moderate but limited to in vitro models. Notably, ellagic acid (**313**) and its derivatives have been shown to induce apoptosis in SNO cells, indicating potential cytotoxic effects that merit further exploration in more advanced preclinical models, including in vivo studies.

While many studies report the antimicrobial potential of the genus *Alchornea*, the observed activity varies significantly across chemotypes. Specifically, species with high tannin content, such as *A. cordifolia*, display broad-spectrum antibacterial effects, likely due to the non-specific membrane interactions typical of polyphenolic compounds. The traditional uses of *Alchornea* species are closely related to their unique chemical profiles, as evidenced by correlations between phytochemical composition and antibacterial activity patterns. Specifically, species rich in alkaloids (*A. glandulosa*) demonstrate selective activity against Gram-positive bacteria, whereas those containing primarily polyphenols and terpenoids (*A. cordifolia*) exhibit broader or Gram-negative-selective activity. This conclusion is drawn from comparative analyses of *Alchornea* species [141,145,173,174]. Pharmacological studies should consider not only the species but also the detailed chemical composition of the plant material, as these factors play crucial roles in determining the range and mechanisms of their biological effects.

The human clinical trial investigating the glycemic effects of *A. cordifolia* was conducted by Otoo et al. In this study, ten healthy human subjects consumed fufu made from cassava, plantain, and a cassava-plantain composite, both with and without the addition of *A. cordifolia* leaf powder. The results revealed that fufu containing *A. cordifolia* produced a significantly lower glycemic response than the non-composited forms, with glycemic index values dropping from 46–53% to 12–14% across all fufu varieties. The observed effect was attributed to flavonoids and tannins in the plant extract, which may inhibit carbohydrate absorption. Thus, this study provides clinical evidence supporting the traditional use of *A. cordifolia* as a modulator of postprandial glycemia in healthy individuals [175]. Similarly, a preclinical in vivo study by Mahama et al. was conducted in murine models. The acute toxicity and anti-plasmodial activity of chloroform fractions from *A. cordifolia* and *C. procera* were assessed using *P. berghei*-infected mice. The results revealed that the chloroform fraction of *A. cordifolia* significantly suppressed parasitemia, ranging from 62.7% to 75.6%, at various dose levels [176]. These findings provide scientific evidence for the traditional medicinal uses of *Alchornea* species, as illustrated in Figure 12 and Table 4, Table 5 and Table 6.

### 5.1. Anti-Microbial Effects

The demand for new anti-infective agents, particularly those effective against Gram-negative and multidrug-resistant (MDR) pathogens, remains a key worldwide health priority [177]. Infectious diseases continue to claim more than 13 million lives each year, a figure that has intensified over the recent few decades due to the growing frequency of resistant microbial strains [178,179]. In this context, natural products resulting from plants and microbes serve as irreplaceable reservoirs for anti-microbial drug discovery, providing structurally diverse bioactive phytoconstituents [180]. Within this framework, several studies have examined the anti-microbial potential of *Alchornea* species, using both crude extracts and pure compounds. These investigations provide promising evidence of the efficacy of *Alchornea* species against a wide range of pathogens, thereby supporting its traditional medicinal uses and inspiring further investigation into its antimicrobial phytochemicals (Table 4).

Compounds **142–143**, **147**, **149**, **151–152**, **159**, **180**, **225**, **229–230**, **231 233**, **261–262**, **264**, **273–276**, **279**, **294**, **295**, **296**, **313–314,** and **324**, isolated from *A. cordifolia*, *A. laxiflora*, and *A. davidii,* showed significant anti-microbial effects against a broad spectrum of strains including, *S. aureus*, *E. coli*, *B. subtilis*, *B. cereus*, *E. faecalis*, *K. pneumonia*, *M. catarrha*, *S. saprophyticus*, *P. mirabilis*, *C. braakii*, *S. typhi*, *P. aeruginosa*, *C. albican*, *A. niger*, *and A. flavus* [46,104,141,150,153,161,164,181]. These findings support the traditional medicinal uses of these species and demonstrate their potential as the basis for new antimicrobial drugs.

The aqueous and ethanolic leaf extracts of *A. cordifolia* have demonstrated antifungal activity against *T. verrusculum* and *T. mentagrophytes*, indicating a broad-spectrum antimicrobial potential [182]. Djimeli et al. reported that an aqueous extract of *A. cordifolia* leaves exhibited bacteriostatic activity against *Escherichia coli*, with a minimum inhibitory concentration (MIC) of 1500 μg/mL. Additionally, the extract was administered orally at doses of 232, 112, and 58 mg/kg and showed dose-dependent efficacy, resulting in the purge of infection within 13 days of treatment [183]. Abo and Ashidi (1999) also investigated the antifungal and antibacterial effects of *A. cordifolia*, finding antifungal effects similar to those of tioconazole and antibacterial effects comparable to those of ampicillin and gentamicin [184]. Moreover, the stem bark and leaf extracts of *A. cordifolia* exhibited significant inhibition of pathogenic bacteria, including *S. aureus* and *P. aeruginosa* [173,185]. In a quantitative study by Okeke et al., 36.5% of bacterial isolates were inhibited at 5.0 mg/mL of leaf extract, increasing to 95.9% at 20.0 mg/mL.

Similarly, extracts of *A. laxiflora* leaves (aqueous, ethyl acetate, and ethanol) were tested against clinical *Salmonella paratyphi* and *Salmonella typhi* strains isolated from human stool samples. The results revealed dose-dependent antibacterial effects at concentrations of 20, 40, and 60 mg/mL. However, the ethyl acetate extract exhibited the highest efficacy at 60.0 mg/mL, with zones of inhibition (ZOI) of 19 mm and 24 mm recorded against *S. paratyphi* and *S. typhi*, respectively [186]. Additionally, the ethanol extract of *A. trewioides* exhibited selective antimicrobial activity, with greater effects against Gram-positive bacteria than against Gram-negative strains [187]. The results revealed that the extracts were rich in sterols, polyterpenes, polyphenols, flavonoids, tannins, alkaloids, and saponins, and they demonstrated notable antibacterial activity. All strains were highly sensitive to *A. cordifolia* extracts at 50 mg/mL. Low MIC values, ranging from 0.78 to 12.5 mg/mL, were observed [125] (Table 4).

**Table 4:** Summary of anti-microbial effects of extracts/pure compounds from the genus *Alchornea*.

Name of Plant	Extracts/Pure Compounds	Study	Model	MIC/ZOI/Doses	Reference
*A. castaneifolia*	ELExt	In vitro	*Emericellopsis donezkii*, *Colletotrichum gloesporioides*	7.8–500.0 μg/mL	[188]
*A. castaneifolia*	EExt	In vitro	*Rhodotorula rubra*, *Candida guilliermondii*, *Candida tropicalis*, *Candida albicans*, *Aspergillus flavus*, *Aspergillus parasiticus*, *Microsporum canis*, *Microsporum gypseum*, *Trichophyton rubrum*, *Trichophyton mentagrophytes*, *E. coli*, *Pseudomonas aeruginosa*	30.0 μg/mL	[53]
*A. coelophylla*	MLExt, HexLExt	In vitro	*Bacillus subtilis*, *Staphylococcus aureus*, *P. aeruginosa*, *E. coli*	4.0 mg/mL	[189]
*A. cordifolia*	ELExt, MLExt, AqLExt	In vitro	*S. aureus*, *E. coli*, *Klebsiella* spp., *P. aeruginosa*, *Staphylococcus epidermidis*, *Acinetobacter baumannii*, *Proteus mirabilis*, *Enterobacter cloacae*, *K. pneumoniae*	62.5–500.0 mg/mL	[190]
*A. cordifolia*	ELExt	In vitro	*E. coli*, *S. aureus*, *E. faecalis*, *B. subtilis*	25.0 mg/mL	[191]
*A. cordifolia*	ELExt, EaLExt	In vitro	*Klebsiella* sp., *E. coli*	2.5–20.0 mg/mL	[192]
*A. cordifolia*	Aq/ELExt	In vitro	*E. coli*, *P. aeruginosa*, *S. aureus*, *C. albicans*	10.0–20.0 mg/mL	[193]
*A. cordifolia*	ELExt, AqLExt	In vitro	*T. mentagrophytes*, *T. verrucosum*	50.0 mg/mL	[182]
*A. cordifolia*	ELExt, AqLExt	In vitro	*P. aeruginosa*	6.2–25.0 mg/mL	[194]
*A. cordifolia*	ELExt, AqLExt	In vitro	*S. aureus*, *P. aeruginosa*, *E. coli*, *Salmonella* sp., *Shigella* sp.	3.1–12.5 mg/mL	[195]
*A. cordifolia*	AqLExt	In vitro	*E. coli*	3.1–50.0 mg/mL	[196]
*A. cordifolia*	MLExt	In vitro	*A. favus*	0.6–40.0 mg mL	[197]
*A. cordifolia*	MLExt, ELExt, EaLExt	In vitro	*S. aureus*, *P. aeruginosa*, *Proteus* sp., *Klebsiella* sp., *E. coli*	32.0–17.0 mm	[198]
*A. cordifolia*	ELExt	In vitro	*S. aureus*, *B. subtilis*, *P. aeruginosa*, *E. coli*, *C. albicans*, *Aspergillus niger*	1.3–10.0 μg/mL	[199]
*A. cordifolia*	ELExt	In vitro	*Bryophyllum pinnatum*, *Acalypha wilkesiana*, *Enterococcus faecalis*	0.2 mg/mL	[200]
*A. cordifolia*	AqLExt	In vitro	*E. coli*, *K. pneumoniae*, *P. aeruginosa*, *S. aureus*	0.8–50.0 mg/mL	[201]
*A. cordifolia*	ELExt, AqLExt	In vitro	*E. coli*, *P. aeruginosa*, *S. aureus*	9.0–20.0 mm	[202]
*A. cordifolia*	ELExt	In vitro	*E. coli*, *K. pneumoniae*, *Providencia rettgeri*, *Proteus mirabili*, *Citrobacter koseri*, *Acinetobacter baumanii*	3.1 mg/mL	[203]
*A. cordifolia*	MLExt	In vitro	*E. coli*, *S. aureus*	50.0 mg/mL	[204]
*A. cordifolia*	AqStBExt, EStBExt	In vitro	*S. aureus*, *B. subtilis*, *Micrococcus* sp., *E. coli*	100.0–200.0 mg/mL	[205]
*A. cordifolia*	Aq/MLExt	In vitro	Gram-positive and negative bacteria	0.08–4.38 mg/mL	[206]
*A. cordifolia*	MLExt, MStBExt	In vitro	*E. coli*, *S. aureus*	64.0 mg/mL	[207]
*A. cordifolia*	MLExt, ChLExt, MStBExt, ChStBExt, MRExt, ChRExt	In vitro	*S. aureus*, *P. aeruginosa*, *E. coli*, *B. subtilis*, and *C. albicans*.	0.5–20.0 mm	[208]
*A. cordifolia*	MSExt	In vitro	*S. typhi*, *Shigella dysenteriae*, *E. coli*, and *S. aureus*	6.2 µg/mL	[209]
*A. cordifolia*	ELExt, EStBExt, AqLExt, AqStBExt	In vitro	*S. aureus*, *S. pneumoniae*, *B. subtilis*, *E. coli*, *P. aeruginosa*, *S. typhi*	1.9–15.6 mg/mL	[210,211]
*A. cordifolia*	ELExt, BuLExt	In vitro	*C. albicans*, *P. aeruginosa*, *S. aureus*, *E. coli*, *T. violaceum*	13.0 mm	[212]
*A. cordifolia*	MFExt, MApExt	In vitro	*S. typhi*, *S. dysenteriae*, *E. coli*, *S. aureus*	3.1–12.5 mg/mL	[213]
*A. cordifolia*	AqLExt, ELExt	In vitro	*S. aureus*	25.0 µg/mL	[214]
*A. cordifolia*	PELExt, MLExt, ChLExt	In vitro	*S. aureus*, *E. faecalis*, *E. coli*, *P. aeruginosa*, *C. albicans*	1.6–25.0 mg/mL	[215]
*A. cordifolia*	**180**, **229**, **230**, **233**	In vitro	*E. coli*, *K. pneumoniae*, *C. braakii*	50.0 µg/mL	[150]
*A. cordifolia*	AqLExt, AqSExt, AqRExt	In vitro	*E. coli*, *C. diversifolia*, *S. dysenteriae*, *S. enteritidis*, *S. aureus*, *S. flexneri*	15.6 µg/mL	[216]
*A. cordifolia*	aqueous and ethanolic extracts of the leaves, stem, and male inflorescence	In vitro	*E. coli*, *S. aureus*, *A. niger*, *Trichoderma viride*	4.1–19.0 mm	[217]
*A. cordifolia*	MLExt, ChLExt	In vitro	*S. mutans*, *Lactobacillus* spp.	20.0 mg/mL	[218]
*A. cordifolia*	AqLExt	In vitro, In vivo	*E. coli*	1500.0 μg/mL (In vitro); 232.0 mg/kg (In vivo)	[183]
*A. cordifolia*	AqLExt, EaLExt	In vitro	*S. aureus*, *Klebsiella* spp., *Staphylococcus*, *Pseudomonas*, *Streptococcus* spp.	25.0–50.0 mg/mL	[8]
*A. cordifolia*	Fresh and concentrated stem juice	In vitro	*Kocuria varians*, *Streptococcus pyogenes*, *K. pneumoniae*, *Klebsiella aerogenes*, *Salmonella enterica*	6.7 ± 1.5–39.0 ± 2.5 mm	[219]
*A. cordifolia*	Essential oils	In vitro	*S. aureus*, *A. niger*, *Bacillus cereus*	39.0–156.0 μg/mL	[124]
*A. cordifolia*	MLExt	In vitro	*Laportea aestuans*, *P. purpureum*, *P. aeruginosa*	64.0 μg/mL	[220]
*A. cordifolia*	**142**, **143**, **147**, **149**, **159**, **231**	In vitro	*B. cereus*, *E. faecalis*, *S. aureus*, *Streptococcus saprophyticus*, *E. coli*, *K. pneumoniae*, *Moraxella catarrhalis*, *Proteus mirabilis*	4.0–16.0 µg/mL	[141]
*A. cordifolia*	ELExt, EStExt, MLExt, MStExt, EaLExt, EaStExt, ChLExt, ChStExt	In vitro	*B. cereus*, *E. faecalis*, *S. aureus*, *Streptococcus saprophyticus*, *E. coli*, *K. pneumoniae*, *Moraxella catarrhalis*, *Proteus mirabilis*	63.0 mg/mL	[141]
*A. cordifolia*	Aq/ESExt	In vitro	Gram-positive and Gram-negative bacteria	3.1–100.0 mg/mL	[221]
*A. cordifolia*	MStBExt, MLExt	In vitro	*S. aureus*	2.81–7.47 mg/mL	[207]
*A. cordifolia*	AqLExt, ELExt	In vitro	*S. aureus*, *S. saprophyticus*, *S. typhi*, *E. coli*, *K. pneumoniae*, *P. aeruginosa*, *P. mirabilis*	12.5 mg/mL	[222]
*A. cordifolia*	**262**, **294**, **295**, **296**, **313**, **324**	In vitro	*B. subtilis*, *S. aureus*, *S. typhi*, *E. coli*, *P. aeruginosa*, *C. albicans*, *A. niger*	105.0–180.0 μg/mL	[161]
*A. cordifolia*	MLExt	In vitro	*B. subtilis*, *S. aureus*, *S. typhi*, *E. coli*, *P. aeruginosa*, *C. albicans*, *A. niger*	-	[161]
*A. cordifolia*	AqLExt	In vitro	*S. aureus*	1.6–3.1 mg/mL	[223]
*A. cordifolia*	ChLExt, MLExt, ELExt, PELExt, AqLExt, ChStExt, MStExt, EStExt, PEStExt, AqStExt	In vitro	Gram-positive and negative bacteria	0.125–1.0 mg/mL	[224]
*A. cordifolia*	HexRExt, BuRExt, ERExt	In vitro	*S. aureus*, *B. subtilis*, *P. aeruginosa*, *C. albicans*	10.0–23.0 mm	[225]
*A. cordifolia*	AqExt, EExt	In vitro	*S. aureus*, *E. coli*, *B. subtilis*	5.0–50.0 mg/mL	[226]
*A. cordifolia*	EStLExt, MStLExt	In vitro	*C. albicans*, *S. aureus*, *B. subtilis*, *E. coli*	3.5–10.0 mg/mL	[227]
*A. cordifolia*	MLExt, ChLExt	In vitro	*E. coli*, *S. aureus*, *P. aeruginosa*, *S. typhi*	50.0 mg/mL	[228]
*A. cordifolia*	ELExt	In vitro	*E. coli*, *S. typhi*, *Shigella flexneri*, *S. aureus*, *K. pneumoniae*, *C. albicans*	3.1–18.0 mg/mL	[229]
*A. cordifolia*	ELExt	In vitro	*E. coli*, *S. aureus*	2.8–53.4 mm	[24]
*A. cordifolia*	HexLExt	In vitro	*P. aeruginosa*, *S. aureus*, *E. coli*	1.2 mg/mL	[230]
*A. cordifolia*	**279**	In vitro	*P. aeruginosa*, *E. coli*, *P. mirabilis*, *K. pneumoniae*, *S. aureus*	6.5–12.5 mg/mL	[164]
*A. cordifolia*	AqLExt, HexLExt	In vitro	*P. aeruginosa*, *E. coli*, *S. aureus*, *K. pneumoniae*	2.5–10.0 mg/mL	[231]
*A. cordifolia*	EaLExt	In vitro	*P. aeruginosa*, *S. aureus*, *E. coli*, *C. albicans*	0.6–10.0 mg/mL	[232]
*A. cordifolia*	ELExt, AqLExt	In vitro	Gram-positive and negative bacteria	0.6–1.5 mg/mL	[233]
*A. cordifolia*	HexBExt, AcBExt, MBExt, AqBExt	In vitro	*S. typhi*, *S. paratyphi A*, *S. paratyphi B*	1.3–2.5 mg/mL	[234]
*A. cordifolia*	AqLExt, ELExt	In vitro	*Helicobacter pylori*, *S. typhi*, *S. enteritidis*, S*. flexneri*, *E. coli*	15.6–250.0 mg/mL	[235]
*A. cordifolia*		In vitro	*S. aureus*	1.6–3.1 mg/mL	[73]
*A. cordifolia*	Aq/ELExt (50%)	In vivo	*S. aureus*	25.0–200.0 mg/kg	[185]
*A. cordifolia*	MLExt, ChLExt, MRExt, ChRExt, MStBExt, CStBExt	In vitro	*P. aeruginosa*, *B. subtilis*, *E. coli*	11.0–30.0 mm	[236]
*A. cordifolia*	PEStBExt, AcStBExt, EStBExt, MStBExt	In vitro	*S. aureus*, *B. subtilis*, *E. coli*, *K. pneumoniae*, *P. aeruginosa*	3.5–12.5 mg/mL	[237]
*A. cordifolia*	Aq/ELExt (50%)	In vitro	Microbial strains	2.5–20.0 mg/mL	[173]
*A. cordifolia*	**225**, **230**	In vitro	*S. aureus*, *B. subtilis*	5.8 mg/mL	[153]
*A. davidii*	**273**	In vitro	*B. subtilis*, *S. aureus*, *Pseudomonas fluorescens*, *A. niger*, *C. albicans*	50.0 µg/mL	[46]
*A. floribunda*	MLExt, MStBExt	In vitro	*S. aureus*, *P. aeruginosa*, *E. coli*, *E. aerogenes*, *Providencia stuartii*	128.0–1024.0 μg/mL	[238]
*A. floribunda*	ELExt, EStExt, ERExt, EaLExt, EaStExt, EaRExt, ChLExt, ChStExt, ChRExt	In vitro	*B. cereus*, *E. faecalis*, *S. aureus*, *S. saprophyticus*, *E. coli*, *K. pneumoniae*, *M. catarrhalis*, *P. mirabilis*	50.0 mg/mL	[30]
*A. floribunda*	HexLExt	In vitro	*E. coli*, *K. pneumoniae*, *S. aureus*, *P. aeruginosa*, *B. subtilis*, *S. kefiri*, A. niger, *C. albicans*	32.0–42.0 mm	[239]
*A. glandulosa*	AqLExt	In vitro	*S. aureus*, *E. coli*	50.0 mg/mL	[98]
*A. hirtella*	AqLExt, ELExt	In vitro	*S. aureus*, *E. coli*, *P. mirabilis*	0.8–2.0 mg/mL	[99]
*A. latifolia*	ChLExt	In vitro	*S. aureus*, *S. pneumoniae*	250.0 ug/mL	[139]
*A. laxiflora*	AqLExt, ELExt, MLExt	In vitro	*S. aureus*, *E. faecalis*, *S. enterica*, *S. Dublin*, *E. coli*, *C. coli*, *Campylobacter jejuni*	0.05–0.07 mg/mL	[240]
*A. laxiflora*	**151**, **152**, **313**, **314**	In vitro	*S. saprophyticus*	4.0 μg/mL	[241]
*A. laxiflora*	HexRExt, ChRExt, EaRExt, ERExt, MRExt, AqRext, HexStExt, ChStExt, EaStExt, EStExt, MStExt, AqStext, HexLExt, ChLExt, EaLExt, ELExt, MLExt, AqLext	In vitro	Gram-positive bacteria and Gram-negative bacteria	50.0–63.0 μg/mL	[241]
*A. laxiflora*	Essential oil	In vitro	*B. subtilis*, *B. cereus*, *E. coli*, *P. aeruginosa*, *S. aureus*	4.0–8.0 mm	[149]
*A. laxiflora*	AqLExt, ELExt	In vitro	*B. subtilis*, *S. aureus*, *E. coli*, *E. faecalis*, *K. pneumoniae*, *S. typhi*	2.5–5.0 mg/mL	[242]
*A. laxiflora*	MLExt, MBExt	In vitro	*P. aeruginosa*, *K. pneumoniae*, *E. aerogenes*, *E. coli*, *P. stuartii*	64.0–1024.0 μg/mL	[243]
*A. laxiflora*	AqLExt, MLExt	In vivo/In vitro	Wistar albino rats/*S. flexneri*	125.0–500.0 mg/kg (In vivo) 512.0–1024.0 μg/mL (In vitro)	[244]
*A. laxiflora*	ELExt	In vitro	*E. coli*, *S. typhi*, *C. albicans*, *K. pneumoniae*, *A. flavus*, *S. aureus*	7.5 mg/mL	[245]
*A. laxiflora*	ELExt, AqLExt, EaLExt	In vitro	*S. typhi*, *S. paratyphi*	20.0–60.0 mg/mL	[186]
*A. laxiflora*	MLExt	In vitro	Gram-positive and Gram-negative bacteria	8.8–35.0 mg/L	[174]
*A. laxiflora*	Aq/ELExt, Aq/EStExt, Aq/ERExt	In vitro	*S. aureus*, *B. subtilis*, *E. coli*, *P. vulgaris*, *P. aeruginosa*, *K. pneumoniae*, *C. albicans*, *A. flavus*	12.5–250.0 μg/mL	[246]
*A. laxiflora*	**261**, **264**, **274**, **275**, **276**	In vitro	*P. aeruginosa*, *S. aureus*, *B. cereus*, *C. albicans*, *A. flavus*, *E. coli*, *B. subtilis*	3.0–125.2 μg/mL	[104]
*A. sidifolia*	ChRExt, ChBExt	In vitro	*Cladosporium sphaerospermum*	Not reported	[247]
*A. sidifolia*	ChLExt	In vitro	*C. sphaerospermum*	Not reported	[143]
*A. trewioides*	EExt	In vitro	*E. coli*, *S. aureus*	0.3125 µg/mL	[187]
*A. trewioides*	EaExt	In vitro	CBRH7919 (Jneo3-5B)	14.6 µg/mL	[248]
*A. triplinervia*	MLExt, ChLExt	In vitro	*S. aureus*, *E. coli*	62.5 μg/mL	[137]
*A. triplinervia*	MLExt	In vivo	*H. pylori*	0.25 mg/mL	[249]
*A. triplinervia*	AqBExt, MBExt, EaBExt	In vitro	*E. coli*, *S. aureus*, *Saccharomyces cerevisiae*, *C. albicans*	0.25–3.0 mm	[250]

MFExt: Methanolic extract of fruits; MApExt: Methanolic extract of aerial parts; ELExt: Ethanol leaf extract; EStBExt: Ethanol stem bark extract; AqLExt: Aqueous leaf extract; AqStBExt: Aqueous stem bark extract; MSExt: Methanol stem extract; Aq/ELExt (50%): Aqueous and ethanol leaf extract; EStExt: Ethanol stem extract; ERExt: Ethanol root extract; EaLExt: Ethyl acetate leaf extract; EaStExt: Ethyl acetate stem extract; EaRExt: Ethyl acetate root extract; ChLExt: Chloroform leaf extract; ChStExt: Chloroform stem extract; ChRExt: Chloroform root extract; MLExt: Methanol leaf extract; MStExt: Methanol stem extract; EaLExt: Ethyl acetate leaf extract; EaStExt: Ethyl acetate stem extract; ChLExt: Chloroform leaf extract; ChStExt: Chloroform stem extract; HexBExt: Hexane bark extract; AcBExt: Acetone bark extract; MBExt: Methanol bark extract; AqBExt: Aqueous bark extract; Aq/ESExt: Aqueous/ethanol stem extract; EStLExt: Ethanol stem leaf extract; MStLExt: Methanol stem leaf extract; Aq/ELExt: Aqueous/ethanol leaf extract; BuRExt: *n*-Butanol root extract; Aq/EStExt: Aqueous/ethanol stem extract; Aq/ERExt: Aqueous/ethanol root extract; AqLExt: Aqueous leaf extract; HexRExt: Hexane root extract; ChRExt: Chloroform root extract; EaRExt: Ethyl acetate root extract; ERExt: Ethanol root extract; MRExt: Methanol root extract; AqRExt: Aqueous root extract; HexStExt: Hexane stem extract; ChStExt: Chloroform stem extract; EaStExt: Ethyl acetate stem extract; EStExt: Ethanol stem extract; MStExt: Methanol stem extract; AqSExt: Aqueous stem extract; HexLExt: Hexane leaf extract; ChLExt: Chloroform leaf extract; EaLExt: Ethyl acetate leaf extract; ELExt: Ethanol leaf extract; MLExt: Methanol leaf extract; AqLExt: Aqueous leaf extract; EaExt: Ethyl acetate extract.

3-*O*-Acetyl-erythrodiol (**142**); 3-*O*-Acetyl-aleuritolic acid (**143**); Friedelin (**147**); Friedelane-3-one-28-al (**149**); 3-*O*-Acetyloleanolic acid (**151**); 3-*O*-Acetyl-ursolic acid (**152**); Stigmasterol (**159**); Deepoxyalchornoic acid (**180**); Protocatechuic acid (**225**); Shikimic acid (**229**); Gallic acid (**230**); Methyl gallate (**231**); 4-*O*-Methylgallic acid (**233**); Quercetin (**261**); Quercetin-3-*O*-*β*-D-glucopyranoside (**262**); Rutin (**264**); Isorhamnetin-3-*O*-*β*-D-xyloside (**273**); Quercetin-3,4′-diacetate (**274**); Quercetin-7,4′-disulphate (**275**); Quercetin-3′,4′-disulphate (**276**); 5′-Methyl-4′-propenoxy anthocyanidine 7-*O*-*β*-D-diglucopyranoside (**279**); Myricetin-3-*O*-*β*-D-glucopyranoside (**294**); Myricetin-3-*O*-*β*-D-galactopyranoside (**295**); Myricetin-3-*O*-*α*-L-rhamnopyranoside (**296**), Ellagic acid (**313**); 3-*O*-Methyl-ellagic acid (**314**); 1-*O*-Galloyl-6-*O*-luteoyl-*β*-D-glucopyranoside (**324**).

### 5.2. Antioxidant Effects

Oxidative stress plays a crucial role in regulating various physiological and pathological functions. Reactive oxygen species (ROS), which are natural byproducts of normal cellular metabolism, play a pivotal role in modulating immune system development and function by activating various cell signaling pathways. However, when ROS production exceeds the cellular antioxidant defense capacity, oxidative stress occurs. This imbalance disrupts the cell’s normal redox state, leading to oxidative damage to cellular macromolecules, including lipids, proteins, and DNA [251]. Oxidative stress is implicated in the pathogenesis of many chronic and degenerative diseases, such as cardiovascular disorders, diabetes, cancer, neurodegenerative diseases, and immune system disorders [252].

Some compounds, especially flavonoids, lignins, and tannins from *Alchornea* species, have shown significant antioxidant effects. Notably, compounds **255**, **259–260**, **262**, **286**, **294–296** (flavonoids), **313**, and **324** (lignins and tannins), isolated from *A. coelophylla*, *A. cordifolia*, *and A. floribunda*, showed significant free radical scavenging and reducing effects as evidenced by DPPH, ABTS, and FRAP assays [31,88,161].

The in vivo antioxidant activity of the methanolic extract of *A. laxiflora* was investigated by evaluating its effects on key antioxidant enzymes, catalase (CAT), reduced glutathione (GSH), and superoxide dismutase (SOD) in experimental animals. Oral administration of the extract at doses of 0.5, 1.0, and 10.5 mg/kg body weight significantly (*p* < 0.05) improved serum CAT, GSH, and SOD levels compared with the control groups, indicating improved endogenous antioxidant defense [253]. Phytochemical screening of the methanolic extract of *A. cordifolia* showed a diverse profile of antioxidant chemical constituents. These extracts exhibited prominent radical-scavenging activity in the DPPH and NO models, consistent with results from other studies [130]. Effo et al. conducted the antioxidant potential of aqueous leaf extract of *A. cordifolia* using ferric reducing antioxidant power (FRAP) and DPPH assays. The aqueous extract alone did not influence serum transaminase levels; however, its co-administration with isoniazid and rifampicin increased alanine aminotransferase (ALT) and aspartate aminotransferase (AST) by more than 48%, an effect that was significantly reduced by the extract. The aqueous extract at 800 mg/kg reduced serum AST and ALT levels by about 45%. Doses of 200–400 mg/kg decreased ALT by more than 40%, indicating hepatoprotective antioxidant effects [254].

Akoto (2019) reported on the antioxidant effects of CHCl_3_, CH_3_OH, and C_6_H_14_ extracts of *A. cordifolia*. In DPPH assays, IC_50_ values were 94.77, 35.22, and 93.02 ppm for CHCl_3_, CH_3_OH, and C_6_H_14_ extracts, respectively. In H_2_O_2_ scavenging assays, IC_50_ values were 626.5, 272.0, and 898.04 ppm [215]. Banso et al. further confirmed that the DPPH inhibition increased with extract concentration. At the same time, the antioxidant effect was lower than that of the standard reference (ascorbic acid) [191]. These findings underscore the significance of *Alchornea* species as promising sources of antioxidants that can alleviate oxidative stress and its associated pathogenic effects. The aqueous leaf extract yielded 17.4% (64.4 g), highlighting water’s efficacy as a solvent for extracting bioactive compounds. Remarkably, the extract demonstrated significant activity [255] (Table 5).

### 5.3. Hepatoprotective Effects

The liver, as a central metabolic organ, plays a crucial role in biotransformation, detoxification, xenobiotic metabolism, and nutrient storage, thereby maintaining systemic homeostasis. Hepatoprotective drugs protect the liver from injury and support its recovery. Hepatic impairment can result from a variety of factors, including pathogens such as viruses, bacteria, and parasites; autoimmune diseases like primary biliary cholangitis and autoimmune hepatitis; exposure to hepatotoxic compounds, particularly carbon tetrachloride (CCl_4_); and excessive alcohol consumption [256]. The use of medicinal plants to treat liver disease has a long tradition across diverse cultures, and plant-derived compounds continue to be widely used in modern ethnomedicine for their hepatoprotective effects [257].

Different fractions derived from the methanol leaf extract of *A. cordifolia* have been investigated for hepatoprotective effects using a CCl_4_-induced hepatotoxicity model in rats. Among the tested fractions, ethyl acetate and acetone showed significant hepatoprotective effects, indicating that these fractions contain bioactive compounds that mitigate liver damage [258]. The hepatoprotective potential of *A. cordifolia* leaves was evaluated in a murine model of acetaminophen-induced hepatotoxicity. The results showed a significant reduction in hepatic damage with the sequential administration of the plant extract, highlighting its traditional medicinal use [259].

Similarly, Jacob et al. reported that ethanol leaf extracts of *A. cordifolia* efficiently protected against acetaminophen-induced liver injury in rats. Histopathological investigation confirmed a reduction in hepatocellular necrosis, tissue degeneration, and inflammatory infiltration, thereby supporting the plant’s traditional medicinal use in the treatment of liver disorders [260]. The methanol extract of *A. laxiflora* has been revealed to significantly modulate key hepatic biochemical markers, including ALT, AST, gamma-glutamyl transferase (GGT), and glutathione S-transferase (GST), indicating potential hepatoprotective effects [261]. The hepatoprotective potential of *A. laxiflora* roots was evaluated in a sodium arsenate-induced hepatotoxicity model. The hexane root extract led to a substantial reduction in serum liver enzymes (ALT, AST, and alkaline phosphatase [ALP]), as well as in hepatic metabolizing enzymes such as GST, cytochrome b5, and nicotinamide adenine dinucleotide (NAD^+^/NADH). Furthermore, improvements were observed in serum total protein, globulin, and albumin levels [262]. These findings provide experimental validation of the ethnomedicinal use of *Alchornea* species for the treatment of liver disease, supporting its therapeutic relevance in traditional medicine (Table 5).

### 5.4. Anti-Inflammatory and Analgesic Effects

Inflammation is a complex biological response that serves as a defense against various harmful stimuli, including pathogens, toxic substances, and environmental stressors. However, chronic or uncontrolled inflammation can lead to several diseases. Multiple species of the genus *Alchornea* have long been used in traditional medicine to treat inflammatory conditions, including pain, rheumatism, and trauma (Figure 2). Modern pharmacological research has validated these conventional claims through in vitro and in vivo studies examining their anti-inflammatory effects.

Specifically, Compounds **131–133**, **143**, **156**, **158**, **163–164**, **249**, and **335–336**, isolated from *A. annamica*, *A. floribunda*, *A. cordifolia*, and *A. glandulosa*, have revealed significant anti-inflammatory effects against lipopolysaccharide (LPS)-induced inflammation in RAW264.7 cells, and egg albumen-induced paw edema in rats [16,91,142,169]. However, the anti-inflammatory effects of *Alchornea* species have been evaluated using various in vitro and in vivo models.

Dzoyem and Eloff (2015) evaluated the anti-inflammatory effects of an acetone extract from *A. laxiflora* leaves through an in vitro assay. The extract demonstrated dose-dependent inhibition of nitric oxide (NO) production in the LPS-activated RAW 264.7 cell line at concentrations of 6.25, 12.5, 25, and 50 μg/mL, with the highest inhibition (96.53%) observed at 25 μg/mL. Simultaneously, an acetone extract significantly inhibited 15-lipoxygenase (15-LOX) effects (IC_50_ = 46.03 μg/mL), compared to quercetin, a standard reference drug (IC_50_ = 35.85 μg/mL) [36]. Furthermore, Ngoungoure et al. reported that a methylene chloride/methanol (1:1, *v*/*v*) leaf extract from *A. laxiflora* inhibited NO production by 68.10% with IC_50_ = 66.57 μg/mL and showed 54.58% inhibition of 15-LOX, with IC_50_ = 90.42 μg/mL [263]. Formagio-Neto et al. also demonstrated both oral and topical anti-inflammatory effects of *A. glandulosa* leaf extracts [169]. In an in vivo study by Mavar-Manga et al., aqueous decoction and methanolic leaf extract of *A. cordifolia* were used, and they significantly decreased Croton oil-induced ear edema in mice, supporting its traditional medicinal use [142].

Additionally, Lopes et al. reported that the ethyl acetate fraction of *A. triplinervia* leaves exhibited significant anti-inflammatory effects, providing scientific support for its ethnomedicinal use in Brazilian traditional medicine [264]. An in vivo evaluation by Okoye et al. on *A. floribunda* methanolic leaf extract and its fractions revealed moderate anti-inflammatory effects at a dose of 200 mg/kg; the extract reduced egg albumin-induced paw edema by 54.69%, compared to the inhibition by aspirin (53.13%) [12]. Moreover, Malara et al. investigated the anti-inflammatory effects of *A. glandulosa*, highlighting the pharmacological significance of medicinal plant biodiversity [265]. Collectively, these findings substantiate the traditional medicinal uses of *Alchornea* species and illustrate their potential as sources for the discovery of anti-inflammatory drugs (Table 5).

### 5.5. Anti-Cancer Effects

Cancer is a complex group of diseases characterized by the aberrant proliferation of malignant or transformed cells, driven by the accumulation of epigenetic and genetic alterations. The growth of malignancy is often linked to genetic mutations that disrupt critical regulatory mechanisms, including those governing cell-cycle development, DNA repair, and apoptosis. When injured or aged cells continue to divide, they do not undergo programmed cell death. This results in the formation of aberrant cells that can ultimately accumulate into a mass, referred to as a tumor. According to current global health statistics, cancer accounts for roughly one in every six fatalities caused by disease, emphasizing its life-threatening public health impact [266,267].

Compounds **137–138**, **151–152**, **199**, **313–314**, **319**, **335–336**, and **356** isolated from *A. cordifolia and A. laxiflora* showed anti-cancer effects against different cell lines [139,144,181,268].

Although different parts of *A. laxiflora* have been traditionally used in ethnomedicine to treat cancer, experimental and clinical research validating its anti-cancer effectiveness remains limited [36]. In the brine shrimp bioassay against *Artemia salina*, the aqueous, ethanolic, and methanolic leaf extracts of *A. laxiflora* showed toxicity, with LC_50_ values of 41.01, 8.91, and 142.40 μg/mL, respectively, signifying the presence of bioactive compounds with potential anti-cancer effects [242,269]. Furthermore, Kuete et al. reported that methanol extracts from the leaves, stems, and roots of *A. laxiflora* exhibited significant cytotoxicity against the drug-sensitive CCRF-CEM human leukemia cell line, with IC_50_ values of 43.67 ± 4.06, 49.21 ± 11.16, and >80 μg/mL, respectively [270].

In two independent studies, Okokon et al. investigated the cytotoxic effect of ethanol extracts and different fractions derived from the roots and leaves of *A. laxiflora* against HEK (human embryonic kidney) and HeLa (human cervical carcinoma) cell lines using the MTT assay. The ethanolic crude root extracts and related fractions showed no significant cytotoxicity toward HeLa cells, with IC_50_ values exceeding 100 μg/mL, showing low toxicity. In contrast, the crude extracts and fractions of the leaves exhibited significant cytotoxicity toward both HEK and HeLa cell lines, with the ethyl acetate fraction showing the highest potency. The ethyl acetate fraction revealed TC_50_ values of 1.41 and 8.83 μg/mL, respectively, indicating a significant cytotoxic effect [131,132]. Siwe Noundou et al. investigated the anti-cancer potential of an extract from the leaves and stems of *A. cordifolia* (100 mg/mL) against three human cancer cell lines: SNO (esophageal carcinoma), MCF-7 (estrogen receptor-positive breast cancer), and MDA-MB-231 (triple-negative breast cancer). The extracts demonstrated mild cytotoxicity against MCF-7 cells, with percent cell viabilities of 93% and 89% for the leaf and stem extracts, respectively. In MDA-MB-231 cells, viability remained modest, at 103% and 100% for the leaf and stem extracts, respectively. In contrast, a more pronounced inhibitory effect was observed in SNO, with percent cell viability ranging from 10% to 50%, indicating a selective cytotoxic response [30] (Table 5). These findings suggest that *Alchornea* species have potential as a source of anti-cancer drugs, warranting further mechanistic and in vivo research.

**Table 5:** Summary of the antioxidant, hepatoprotective, anti-inflammatory, analgesic, and anti-cancer effects of extracts and pure compounds from the genus *Alchornea*.

Name of Plant	Extracts/Compounds	Study	Model	Dosage/Conc.	IC_50_/EC_50_/Results	References
*** Antioxidant effects ***						
*A. coelophylla*	HexExt, DExt, MExt	In vitro	DPPH and ABTS^+^ assay	41.1–111.9 mg/L	IC_50_ = 20.181 ± 0.003%, 57.012 ± 0.002% and 56.103 ± 0.001%	[271,272]
*A. coelophylla*	**286**	In vitro	DPPH and ABTS^+^ assay	50.0–500.0 µg/mL	Compound **286** showed DPPH^•^ scavenging activity with IC_50_ = 7.528 μg/mL and ABTS^•+^ scavenging activity with IC_50_ = 379.7 μg/mL; DPPH radical scavenging activity of 53.210 ± 0.002%, 54.890 ± 0.003%, and 18.760 ± 0.004%, respectively.	[88]
*A. coelo-phylla*	MLExt, DLExt, HexLExt, MTExt, DTExt, HexTExt	In vitro	DPPH and ABTS^+^ assay	500.0–1000.0 µg/mL	IC_50_ = 56.103 ± 0.001%, 20.181 ± 0.003% and 57.012 ± 0.002%.	[88]
*A. cordifolia*	AqExt	In vitro	DPPH and ABTS assay	10.0–50.0 mg/mL	The fatty acids in *A. cordifolia* essential oil inhibited key digestive enzymes involved in carbohydrate digestion in vitro and exhibited strong antioxidant DPPH/ABTS^+^ activity.	[273]
*A. cordifolia*	AqLExt	In vitro	DPPH and FRAP assay	0.02–0.10 mg/mL	Aqueous leaf extract of *A. cordifolia* showed potent in vitro antioxidant activity.	[255]
*A. cordifolia*	ELExt	In vitro	DPPH assay	50.0–250.0 µg/mL	The antioxidant effect was lower than that of the standard reference ascorbic acid.	[191]
*A. cordifolia*	ELExt	In vitro	FRAP assay	0.5–5.0 mg/mL	FRAP antioxidant activity of 125.34 μmol/g Fe^2+^ equivalents.	[274]
*A. cordifolia*	MLExt	In vitro	DPPH assay	0.2–1.0 mg/mL	DPPH radical scavenging activity, with scavenging rates of 11.6 2 ± 1.01% at 0.2 mg/mL, 29.68 ± 1.49% at 0.4 mg/mL, 34.09 ± 3.30% at 0.6 mg/mL, 41.87 ± 4.61% at 0.8 mg/mL, and 55.70 ± 5.62% at 1 mg/mL.	[130]
*A. cordifolia*	MLExt, EaLExt	In vitro	DPPH and ABTS assay	93.0–105.4 µg/mL	DPPH: MLExt 500.38 ± 1.28 mg TE/g, EaLExt (188.94 ± 0.15 mg TE/g). ABTS: MLExt (900.64 ± 0.69 mmol TE/g), EaLExt (357.98 ± 0.76 mmol TE/g).	[127]
*A. cordifolia*	ChLExt, MLExt, PELExt	In vitro	DPPH and H_2_O_2_ assays	6.3–100.0 µg/mL	In DPPH assays, IC_50_ values were 94.77, 35.22, and 93.02 ppm. In H_2_O_2_ scavenging assays, IC_50_ values were 626.5, 272.0, and 898.04 ppm	[215]
*A. cordifolia*	AqLExt	In vitro	DPPH assay	0.00625–0.1 mg/mL	The aqueous extract of *A. cordifolia* leaves showed potent dose-dependent antioxidant activity.	[254]
*A. cordifolia*	**262**, **294**, **295**, **296**, **313**, **324**	In vitro	DPPH assay	1.6–3.1 μg/mL	IC_50_ values in the range of 1.56 to 3.11 μg/mL.	[161]
*A. cordifolia*	AcLExt, EaLExt	In vitro	DPPH assay	1.6–50.0 mg/mL	The extract fractions exhibited significant DPPH free-radical scavenging activity, comparable to that of ascorbic acid.	[258]
*A. cordifolia*	ELExt	In vitro	DPPH assay	62.5–500.0 μg/mL	DPPH radical scavenging activity of 68.98% at 0.5 mg/mL.	[275]
*A. cordifolia*	AqLExt, EaLExt	In vitro	H_2_O_2_ and HOCI	6.5 mg/L	The leaf extract of *A. cordifolia* exhibited dose-dependent antioxidant activity, likely due to its high phenolic and flavonoid content.	[276]
*A. cordifolia*	AqEStBExt	In vitro	DPPH, ABTS, and FRAP assays	15.6–2000.0 μg/mL	12.606 ± 0.415 μg/mL (DPPH), 1.330 ± 0.090 μg/mL (ABTS), 11.386 ± 0.155 μg/mL (FRAP).	[277]
*A. cordifolia*	AqEStBExt	In vitro	DPPH assays	0.05 mg/mL	IC_50_ = 0.47 ± 0.2725 mg/mL, EC_50_ = 1.175.	[278]
*A. cordifolia*	WpExt	In vitro	TBARS	0.67 mg/mL	*A. cordifolia* leaf extract exhibited strong, dose-dependent antioxidant activity.	[259]
*A. cordifolia*	ELExt	In vitro	DPPH assay	0.062 mg/mL	*A. cordifolia* leaf extract exhibited in vitro antioxidant activity and enhanced hepatic detoxification mechanisms in vivo.	[279]
*A. floribunda*	AqFExt, AqStBExt	In vitro	DPPH assay	5.0–100.0 µg/mL	IC_50_ = 18.8909 ± 0.1045-35.89 ± 0.21 μg/mL	[280]
*A. floribunda*	**255**, **259**, **260**	In vitro	DPPH and FRAP assay	10.0–100.0 µg/mL	DPPH EC_50_ range: 19–88 μg/mL; FRAP EC_50_ range: 46–88 μg/mL	[31]
*A. glandulosa*	AqLExt	In vitro	DPPH assays	0.5–100.0 μg/mL	IC_50_ =1.3 ± 0.4 μg/mL	[98]
*A. laxiflora*	Aq/ELExt	In vitro	DPPH assay	EC 106.74 ± 0.29	EC_50_ = 12.97 ± 0.30 and 24.34 ± 0.33 μg/mL	[106]
*A. laxiflora*	HexApExt, EaApExt, BuApExt, AqApExt	In vitro	Ferric thiocyanate FTC method	50.0–500.0 μg/mL	Extracts of Nigerian medicinal plants, including *A. laxiflora*, exhibited dose-dependent in vitro antioxidant activity, indicating strong free-radical scavenging potential.	[281]
*A. laxiflora*	MLExt, MRExt, HexLExt, HexRExt	In vitro	ABTS, TBARS, and Thiocyanate assay	1.0–2.5 mg/mL	*A. laxiflora* extracts showed strong in vitro antioxidant activity at 0.05% (*v*/*v*).	[105]
*A. laxiflora*	MLExt	In vivo	Wistar rats	0.1–50.0 mg/kg	Catalase (CAT), reduced glutathione (GSH), and superoxide dismutase (SOD) in experimental animals. Oral administration of the extract at doses of 0.5, 1.0, and 10.5 mg/kg body weight significantly (*p* < 0.05) improved serum CAT, GSH, and SOD levels, indicating an improvement in the endogenous antioxidant defense system.	[253]
*** Hepatoprotective effects ***						
*A. cordifolia*	MLExt	In vivo	Wistar rats	200.0–400.0 mg/kg	*A. cordifolia* leaves dose-dependently protected against sodium arsenite–induced liver damage in rats, reducing serum liver enzymes and improving hepatic histology.	[282]
*A. cordifolia*	AqLExt	In vivo	Wistar rats	100.0–200.0 mg/kg	AqLExt (100–200 mg/kg) dose-dependently reduced CCl_4_-induced elevated liver enzymes in rats (*p* < 0.05) and improved liver histology.	[283]
*A. cordifolia*	ELExt	In vivo	Wistar rats	342.0 mg/kg	ELExt significantly reduced diclofenac-induced elevated ALT, AST, ALP, and bilirubin, and increased albumin (*p* < 0.05) in rats, and alleviated liver histological lesions.	[284]
*A. cordifolia*	MLExt	In vivo	Wistar rats	500.0–2000.0 mg/kg	MLExt dose-dependently protected rats against paracetamol-induced liver injury (*p* ≤ 0.05), reducing elevated ALT, ALP, and AST levels with 68.38–89.71% protection, and restoring normal liver morphology.	[285]
*A. cordifolia*	MLExt	In vivo	Rats	200.0–800.0 mg/kg	The extract of *A. cordifolia* showed dose-dependent hepatoprotective activity.	[254]
*A. cordifolia*	ELExt	In vivo	Rats	200.0–500.0 mg/kg	*A. cordifolia* leaf extract showed dose-dependent hepatoprotective activity in rats.	[260]
*A. cordifolia*	EaLExt, ChLExt	In vivo	Rats	300.0 mg/kg	EaLExt reduced CCl_4_-induced liver enzyme elevation in rats, comparable to silymarin. ChLExt alleviated CCl_4_-induced liver injury by lowering serum ALT, AST, ALP, and bilirubin.	[258]
*A. cordifolia*	HexExt, ChExt, EaExt, AcExt, MExt	In vivo	Albino rats	300.0 mg/kg	*A. cordifolia* extract significantly protected against CCl_4_-induced liver injury in rats, likely via antioxidant activity.	[258]
*A. cordifolia*	AqLExt	In vivo	Wistar rat	0.7 mg/mL	*A. cordifolia* leaf extract showed strong, dose-dependent antioxidant activity, providing the greatest protection against lipid peroxidation among the plants tested.	[259]
*A. cordifolia*	ELExt	In vivo	Rats	200.0–500.0 mg/kg	ELExt significantly (*p* < 0.05) and dose-dependently reduced acetaminophen-induced elevated serum ALT, AST, ALP, cholesterol, and bilirubin in rats.	[286]
*A. laxiflora*	HexRE	In vivo	Wistar rats	0.1–100.0 mg/kg	*A. laxiflora* showed dose-dependent hepatoprotection in sodium arsenate–treated rats.	[262]
*A. laxiflora*	MLE	In vivo	Wistar rats	0.1–50.0 mg/kg	*A. laxiflora* dose-dependently protected against CCl_4_-induced liver and reproductive toxicity in rats, improving liver enzyme levels and sperm quality.	[261]
*A. laxiflora*	HexLE	In vivo	Albino rats	0.5–10.0 mg/kg	*A. laxiflora* dose-dependently protected rats against sodium arsenate–induced liver toxicity, reducing serum and liver enzyme levels.	[287]
*A. laxiflora*	EaLExt	In vivo	Wistar rats	100.0–200.0 mg/kg	EaLExt reduced CCl_4_-induced elevations in AST, ALT, ALP, and LDH (*p* < 0.05) in rats, protected the liver, and promoted hepatocyte regeneration.	[160]
*A. laxiflora*	MLExt	Ex vivo	Wistar rats	10.0–200.0 μg/mL	MLExt potently inhibited rat hepatic microsomal lipid peroxidation (non-enzymatic IC_50_ = 6.95 ± 4.31 μg/mL; enzymatic IC_50_ = 21.64 ± 1.15 μg/mL) and BSA carbonyl formation (IC_50_ = 6.43 ± 0.18 μg/mL), with dose-dependent efficacy.	[114]
*A. trewioides*	AqRExt	In vivo	Rats	0.9–3.6 g/kg	AqRExt dose-dependently alleviated alcoholic liver fibrosis in rats, reducing elevated ALT/AST levels, decreasing hepatic fibrosis markers, and improving liver function (*p* < 0.05 to *p* < 0.001).	[45]
*** Anti-inflammatory and analgesic effects ***						
*A. annamica*	**131**, **132**, **133**	In vitro	LPS-induced RAW264.7 cell	Not reported	IC_50_ values range from 77.40 to 82.16 μM.	[16]
*A. castaneifolia*	AqLExt	In vitro, In vivo	Bovine seminal vesicle microsomes and mice	Not reported	*A. castaneifolia* exhibits notable anti-inflammatory activity in both in vitro and in vivo models.	[288]
*A. cordifolia*	AqLExt	In vitro	Albumin denaturation, Anti-proteinase, Membrane stabilization	0.2–1.0 mg/mL	*A. cordifolia* extract exhibited significant in vitro anti-inflammatory activity, particularly in membrane-stabilization assays.	[255]
*A. cordifolia*	Aq/ELExt	In vivo	Rats (edema inhibition)	100.0–400.0 mg/kg	Aq/ELExt pretreatment significantly reduced ACF-induced paw edema in rats (100 mg/kg most effective) and increased hepatic GSH, CAT, and SOD, suggesting antioxidant-mediated anti-arthritic activity.	[289]
*A. cordifolia*	**143**, **156**, **335**, **336**	In vivo	Mouse ear edema	90.0 μg/cm^2^	Inhibited croton oil-induced mouse ear edema (90 μg/cm^2^) with 28–54% inhibition, equivalent to 71–138% of indomethacin’s activity.	[142]
*A. cordifolia*	HexLExt	In vivo	Egg-albumen-induced rat hind paw edema	50.0–60.0 mg/kg	HexLExt showed significant dose-dependent inhibition of edema (*p* < 0.01).	[290]
*A. cordifolia*	MLExt, EaLExt, HexLExt	In vivo	Mice (oedema inhibition)	90.0 μg/cm^2^	63% and 49%, respectively, for MLExt and HexLExt.	[167]
*A. cordifolia*	AqLExt, MLExt, EaLExt, ChLExt	In vivo	Rats (oedema inhibition)	50.0–100.0 mg/kg	Significant dose-dependent inhibition of egg-albumen-induced rat paw oedema (*p* < 0.01); EaLExt: Reduced CCl_4_-induced elevated AST, ALT, ALP, and LDH levels (*p* < 0.05) in rats, protected the liver, and promoted hepatocyte regeneration.	[291]
*A. floribunda*	**158**, **163**, **164**, **249**	In vivo	Egg albumen-induced paw edema in rats	20.0–50.0 mg/kg	32.2–50.9% paw edema inhibition (20 mg/kg) and 37.23–81.45% ear edema inhibition (50–100 μg/ear); 15.5–51.4% paw edema inhibition (25–50 mg/kg).	[90,91]
*A. floribunda*	HexLExt, EaLExt	In vivo	egg albumen-induced edema	200.0 mg/kg	*A. floribunda* leaf extracts and fractions exhibited significant in vivo anti-inflammatory activity, markedly inhibiting edema formation and leukocyte migration in rats.	[29]
*A. glandulosa*	MExt, HexExt	In vivo	male Swiss mice	25.0–100.0 μg/mL	MExt and HexExt exhibited in vivo anti-inflammatory effects by inhibiting NO production and reducing TNF-*α* production.	[265]
*A. glandulosa*	**336**	In vivo	Carrageenan-induced paw edema	5.0–30.0 mg/kg	**336**: 40.36–89.39% inhibition in paw edema, MPO activity, and leukocyte migration.	[169]
*A. glandulosa*	MLExt, EaLExt	In vivo	Carrageenan-induced paw edema	30.0–300.0 mg/kg	MLExt: 30.32–80.90% inhibition in paw edema, ear edema, leukocyte migration, and joint edema; EaLExt: 31–83.17% inhibition in paw edema, leukocyte migration, and joint edema;	[169]
*A. glandulosa*	EaLExt	In vitro	Murine peritoneal macrophages (NO, H_2_O_2_, TNF-α production inhibition)	8.8% –70.6%	EaLExt inhibited H_2_O_2_ production by 8.59–70.56%, NO production by 16.06–38.73%, and TNF-*α* production by 12.21–15.16% at 1.95–62.50 μg/mL (*p* < 0.01).	[292]
*A. laxiflora*	EStBExt	In vivo	Formalin-induced hind paw licking and thermally induced pain models	141.0–424.0 mg/kg	EStBExt exhibited significant, dose-dependent analgesic effects in mice across multiple pain models.	[293]
*A. laxiflora*	DLExt, MLExt	In vitro	LPS activated RAW 264.7 cells (NO production inhibition)	12.5–100.0 µg/mL	NO production by 68.10% with IC_50_ = 66.57 μg/mL.	[263]
*A. laxiflora*	AcLExt	In vitro	LPS activated RAW 264.7 cells (NO production inhibition)	100.0 μg/mL	The extract demonstrated dose-dependent inhibition of nitric oxide (NO) production in the LPS-activated RAW 264.7 cell line at concentrations of 6.25, 12.5, 25, and 50 μg/mL, with the highest inhibition (96.53%) observed at 25 μg/mL.	[36]
*A. triplinervia*	EaLExt	In vitro	Hydrogen peroxide (H_2_O_2_), nitric oxide (NO), and tumor necrosis factor-*α* (TNF-*α*) production	15.6–62.5 μg/mL	EaLExt inhibited H_2_O_2_ production by 69.64–72.25%, NO production by 47.8–76.77%, and TNF-*α* production by ~18% at 15.62–62.5 μg/mL (*p* < 0.01).	[264]
*** Anti-cancer effects ***						
*A. cordifolia*	EaExt, MExt	In vitro	Hep-G2 cells	100.0 μg/mL	The extracts of *A. cordifolia* showed significant in vitro anti-cancer activity against HepG2 cells.	[127]
*A. cordifolia*	MBExt, MLExt, MRExt	In vitro	CEM/ADR5000, MDA-MB-231-pcDNA, MDA-MB-231-BCRP Degree of resistance, HepG2 Degree of resistance	0.6–80.0 μg/mL	MLExt, MBExt, and MRExt exhibited significant cytotoxicity against the drug-sensitive CCRF-CEM human leukemia cell line, with IC_50_ values of 43.67 ± 4.06, 49.21 ± 11.16, and >80 μg/mL, respectively	[270]
*A. cordifolia*	HexLExt	In vitro	Cancer cell lines (Hs683, U373, MCF7, B16F10)	1.0 mL/200.0 g body weight	HexLExt of *A. cordifolia* exhibits significant cytotoxicity against multiple human cancer cell lines.	[294]
*A. cordifolia*	MLExt, MStExt	In vitro	Cancer cell lines (MDA-MB-231 and MCF-7); SNO oesophageal cancer cell line	100.0 μg/mL; 0.8–1.0 mg/mL	The extracts showed mild cytotoxicity in MCF-7 (93% and 89% viability for leaf and stem, respectively), little effect in MDA-MB-231 (103% and 100%), and strong inhibition in SNO (10–50% viability), indicating selective cytotoxicity.	[30]
*A. cordifolia*	**335**, **336**	In vitro	Cancer (Mel-5, HeLa and J774) and non-cancer (WI38) cells	3.1–50.0 μg/mL	IC_50_ = 0.7–14.3 μg/mL	[268]
*A. cordifolia*	HexExt, MExt, EaExt, ChExt	In vitro	Cancer (Mel-5, HeLa and J774) and non-cancer (WI38) cells	100.0 μg/mL	NA	[268]
*A. latifolia*	**137**, **138**	In vitro	Hep-G2 cells, A 431 cells	250.0 μg/mL	Exhibited in vitro cytotoxicity against Hep-G2 and A-431 cells with IC_50_ values ranging from 3.2 to 12.5 μg/mL.	[139]
*A. laxiflora*	**151**, **152**, **313**, **314**	In vitro	HeLa cells, resazurin reduction assay	20.0 µM	Isolated compounds showed low inhibition of HeLa cells (survival rate range: 99.57 ± 10.87% to 138.58 ± 2.44%), indicating they were non-cytotoxic.	[241]
*A. laxiflora*	HexRExt, ChRExt, EaRExt, MRExt, ERExt, AqRExt, MStBExt	In vitro	HeLa cells, resazurin reduction assay	25.0 μg/mL	NA	[241]
*A. laxiflora*	ERExt, PEExt, DRExt, EaExt, BuExt, AqExt	In vitro	HeLa cells, MTT assay	100.0 μg/mL	The extracts of *A. laxiflora* showed low toxicity.	[131]
*A. laxiflora*	ELExt, PEExt, ChLExt, EaExt, BuExt, AqExt	In vitro	HeLa cells, MTT assay; HEKS cells, MTT assay	100.0 μg/mL	EaExt revealed TC_50_ values of 1.41 and 8.83 μg/mL in HEKS and HeLa cell lines, respectively.	[132]
*A. laxiflora*	AqLExt, ELExt	In vitro	Brine shrimp lethality assay	1.0, 10.0, 100.0, 1000.0 μg/mL	AqLExt and ELExt showed toxicity, with LC_50_ values of 41.01 and 8.91 μg/mL, respectively, indicating the presence of bioactive compounds with potential anti-cancer effects.	[242]
*A. laxiflora*	MLExt	In vitro	Brine shrimp lethality assay	1.6–5000.0 μg/mL	MLExt showed toxicity, with an LC_50_ of 142.40 μg/mL, signifying the presence of bioactive compounds with potential anti-cancer effects.	[269]
*A. laxiflora*	MRExt, MStbExt, MLExt	In vitro	CCRF-CEM cells, resazurin reduction assay	80.0 μg/mL	Extracts showed low inhibition of HeLa cells, with a survival rate of 85–95%, indicating non-cytotoxicity.	[270]
*A. laxiflora*	**199**, **328**, **151**, **152**, **314**, **319**	In vitro	HL-60 cells, MTT assay	Not reported	Isolated compounds showed cytotoxicity against HL-60 cells, with IC_50_ values ranging from 6.6 to 58.7 μM.	[144]

HexExt: Hexane extract; DExt: DCM extract; MExt: Methanol extract; MLExt: Methanol leaf extract; DLExt: DCM leaf extract; HexLExt: Hexane leaf extract; MTExt: Methanol twig extract; DTExt: Dichloromethane twig extract; HexTExt: Hexane twig extract; AqLExt: Aqueous leaf extract; ELExt: Ethanol leaf extract; EaLExt: Ethyl acetate leaf extract; ChLExt: Chloroform leaf extract; PELExt: Petroleum ether leaf extracts; AcLExt: Acetone leaf extract; Aq/EStBExt: Aqueous and ethanol stem bark extract; WpExt: Whole plant extract; AqFExt: Aqueous fruit extract; AqStBExt: Aqueous stem bark extract; Aq/ELExt: Aqueous and ethanol leaf extract; HexApExt: Hexane aerial parts extract; EaApExt: Ethyl acetate aerial part extract; BuApExt: Butanol acetate aerial parts extract; AqApExt: Aqueous aerial parts extract; MRExt: Methanol root extract; HexRExt: Hexane root extract; ChExt: Chloroform extract; EaExt: Ethyl acetate extract; AcExt: Acetone extract; AqRExt: Aqueous root extract; LExt: Leaf extract; RExt: Root extract; BExt: bark extract; MBExt: Methanol bark extract; MStExt: Methanol stem extract; ChRExt: Chloroform root extract; EaRExt: Ethyl acetate root extract; ERExt: Ethanol root extract; MStBExt: Methanol stem bark extract; PEExt: Petrolium ether extract; DRExt: Dichloroform root extract; BuExt: Butanol extract; AqExt: Aqueous extract.

Alnamicosides B (**131**); Blumenol C (**132**); Alnamicosides A (**133**); Seco-3,4-Friedelin (**137**); Seco-3,4-Taraxerone (**138**); Acetyl-aleuritolic acid (**143**); 3-*O*-Acetyl-oleanolic acid (**151**); 3-*O*-Acetyl-ursolic acid (**152**); *β*-Sitosterol (**156**); 3*β*-Hydroxy-5*α*-stigmastane-24-ene (**158**); 5*α*-Stigmastane-3,6-dione (**163**); 5*α*-Stigmastane-23-ene-3,6-dione (**164**); (10*Z*)-Tetradec-10-enoic acid-(2*S*)-2-carboxy-2-hydroxyethyl ester (**199**); Kaempferol-3-*O*-*a*-L-rhamnopyranoside (**249**); Ellagic acid (**313**); 3-*O*-Methylellagic acid (**314**); 3-*O*-Methylellagic acid-3′-*O*-*α*-rhamnopyranoside (**319**); Pterogynidine (**335**); *N*1, *N*2, *N*3-Triisopentenyl guanidine (**336**); (2*R*)-2-Hydroxy-*N*-[(2*S*,3*S*,4*R*,15*Z*)-1,3,4-trihydroxy-15-triaconten-2-yl]octacosamide (**356**).

### 5.6. Anti-Malarial and Anti-Plasmodial Effects

Malaria remains a significant public health challenge, mainly in tropical and subtropical regions. It is triggered by protozoan parasites of the *Plasmodium* species, which are transmitted to humans via the bite of an infected female *Anopheles* mosquito. The principal species responsible for malaria include *Plasmodium falciparum*, *Plasmodium malariae*, *Plasmodium vivax*, *Plasmodium ovale*, *and Plasmodium knowlesi*. Among these, *P. falciparum* is the most virulent, responsible for the highest rates of morbidity and fatalities globally [295]. Although traditional antimalarial therapies have been effective, their use is often constrained by adverse effects, resistance development, and accessibility challenges. Consequently, the urgent need for more safe and efficient antiprotozoal agents has accelerated research into new therapeutic compounds, particularly those derived from natural sources [296].

Numerous studies investigating *Alchornea* species have focused on their anti-malarial and anti-plasmodial potential. Muganza et al. demonstrated that aqueous extracts from the leaves and roots of *A. floribunda* showed significant in vivo anti-malarial and anti-trypanosomal effects. The extracts efficiently decreased parasitemia levels of *Trypanosoma brucei*, *Trypanosoma cruzi*, and *Leishmania infantum*, with IC_50_ values of 19.65, 37.26, and >64 µg/mL, respectively. Additionally, the extracts exhibited a remarkable anti-malarial effect against the chloroquine-resistant K1 strain of *P. falciparum*, with an IC_50_ value of 20.80 µg/mL [33]. Similarly, Mesia et al. reported that the methanol extract of *A. cordifolia* inhibited the growth of *P. falciparum* with an IC_50_ of 2.8 mg/mL [89]. Additionally, the anti-malarial effects of *A. cordifolia* reported by Ayisi et al. showed that an aqueous fruit extract efficiently inhibited the growth of *P. falciparum* 3D7, with an EC_50_ value of 4.9 mg/mL [297].

The in vitro anti-malarial effects of *A. laxiflora* root extracts were evaluated by Okokon et al. Both the crude extract and fractions showed moderate inhibitory activity against chloroquine-resistant (Pf-INDO) and chloroquine-sensitive (*P. falciparum* 3D7) strains. Among the tested fractions, the ethyl acetate extract displayed the most potent activity, with IC_50_ values of 40.17 ± 0.78 μg/mL and 38.44 ± 0.89 μg/mL against the INDO and 3D7 strains [131]. Oluyemi and Blessing investigated the methanol and chloroform leaf extracts of *A. laxiflora,* which were administered orally at doses of 200 to 600 mg/kg to *P. berghei-*infected mice in a comparative anti-malarial potential study. After a five-day treatment period, the methanol extract showed significantly greater (*p* < 0.05) prophylactic, suppressive, and curative anti-malarial effects compared to the chloroform extract [298]. The stembark extract (141–424 mg/kg, p.o.), with an LD_50_ of 1414.21 mg/kg, showed significant activity against *P. berghei* infection in suppressive, prophylactic, and curative tests, with the methanol and dichloromethane fractions exhibiting the highest activity. These results suggest that the stembark extract and fractions of Alchornea laxiflora have antimalarial properties, supporting its traditional use in ethnomedicine for the treatment of malaria [293]. These findings collectively support the ethnopharmacological uses of *Alchornea* species for the treatment of malaria and highlight their potential as a source of bioactive anti-malarial agents (Table 6).

### 5.7. Anti-Diabetic Effects

Diabetes mellitus (DM) is a chronic metabolic disease characterized by persistent hyperglycemia resulting from insufficient insulin secretion, peripheral insulin resistance, or both. The disease is generally classified into two major types: Type 1, which is primarily driven by autoimmune-mediated destruction of the *β*-islets of Langerhans, leading to insulin deficiency; and Type 2, which is associated with peripheral insulin resistance and relative insulin deficiency. It is often associated with obesity and a sedentary lifestyle [299]. Several medicinal plants have been scientifically investigated for their anti-diabetic effects, with mechanisms of action that include improving insulin sensitivity, enhancing insulin secretion, inhibiting intestinal glucose absorption, inhibiting carbohydrate-digesting enzymes, and promoting pancreatic *β*-cell regeneration [300].

The traditional use of *A. laxiflora* leaf decoction for the treatment of diabetes is prevalent among the Badagry community in Nigeria, and this ethnomedicinal practice has been scientifically validated by in vitro and in vivo research. Ogbole et al. reported that the methanol leaf extract of *A. laxiflora* exhibited moderate inhibitory effects against α-amylase, an enzyme involved in carbohydrate digestion, with an IC_50_ of 295.60 μg/mL [269]. Similarly, *A. cordifolia* has long been used in ethnomedicine to treat diabetes. Mohammed et al. evaluated the anti-diabetic effects of the n-butanol extract of A. *cordifolia* leaves in streptozotocin-induced diabetic Wistar rats. The extract efficiently reduced blood glucose levels, showing comparable effectiveness to glibenclamide (10 mg/kg), a standard oral anti-diabetic agent [301]. Thomford et al. used a dexamethasone-induced diabetic rat model to study the anti-diabetic effects of *A. cordifolia*. The ethanol extract significantly lowered blood glucose level, supporting its traditional medicinal use as an anti-diabetic agent [157] (Table 6).

### 5.8. Anti-HIV Effects

Acquired immunodeficiency syndrome (AIDS) is triggered by human immunodeficiency virus (HIV) infection and is characterized by progressive immune system damage and increased exposure to opportunistic infections. Different studies indicate that non-AIDS-related problems are closely linked to the relative abundance of intestinal microbiota, to modifications in its composition, and to chronic immune activation driven by intestinal microbiota translocation [302]. The increased incidence and mortality of HIV-related diarrhea have been linked to disruption of the intestinal epithelial barrier, dysbiosis, depletion of CD4^+^ T lymphocytes resulting from HIV infection, and immune tissue damage [303]. Nature has long served as a reservoir of bioactive chemical constituents with therapeutic effects, including several medicinal plants known for their anti-HIV activity and low toxicity [304].

Compounds **142–143**, **147**, **149**, **151–152**, **159**, **231**, and **313–314**, isolated from *A. cordifolia and A. laxiflora*, showed significant effects against HIV-1 integrase strand transfer assays [181,241]. Additionally, the aqueous seed extract from A. *cordifolia* exhibited significant anti-HIV effects in vitro. It was found to inhibit HIV-1 reverse transcriptase activity, with effective EC_50_ values ranging from 0.01 to 0.03 mg/mL [305] (Table 6).

### 5.9. Anti-Ulcerogenic Effects

A peptic ulcer (PU) refers to a group of chronic gastrointestinal diseases characterized by the erosion of the mucosal integrity of the stomach and/or duodenum lining. It remains a significant cause of morbidity as well as mortality worldwide [306]. The incidence of gastric ulcers is further attributed to physiological disturbances, including lipid peroxidation, oxidative stress due to excessive ROS generation, and increased gastric acidity. Exogenic factors, including alcohol consumption, non-steroidal anti-inflammatory drug (NSAID) usage, bacterial infection, bile reflux, and psychological stress, also contribute significantly to ulcer formation. In this context, medicinal plants are characterized as promising candidates. For centuries, phytotherapeutics have formed the foundation of traditional medicinal systems across diverse cultures, offering a wide range of bioactive compounds with gastroprotective effects [307].

Due to the side effects and limitations associated with traditional therapies, there is an increasing demand for effective and safe therapeutic drugs for the treatment of peptic ulcers. In an in vivo mouse model, the hydroalcoholic leaf extract and the flavonoid-enriched fraction of *A. castaneifolia*, administered at 100–500 mg/kg, significantly promoted ulcer healing. This was achieved by stimulating protective factors, such as somatostatin and prostaglandins, and by inhibiting gastrin secretion [52]. Similarly, polyherbal formulations have shown significant gastroprotective effects. In this study, three different ulcer models were investigated, and the formulations significantly inhibited ulcer formation (*p* < 0.05), with EXR-HF4 showing the most excellent anti-ulcer effect among the tested combinations. These findings indicate that EXR-HF4 has potential as an adjunctive therapy or as a substitute for conventional anti-ulcer agents [308]. Consistent outcomes were attained in ethanol and HCl/ethanol-induced ulcer models using *A. triplinervia*. At doses of 500 and 1000 mg/kg of the methanol extract, the treatment conferred 85–86% and 89–90% gastroprotection, respectively. These results support the gastroprotective effects of *A. triplinervia*, mainly against ethanol-induced gastric mucosal injury [249]. Furthermore, a single oral dose of *A. glandulosa* at 250 mg/kg/day significantly increased gastric epithelial cell production, thereby accelerating the healing of acetic acid-induced gastric ulcers. Notably, subacute toxicity studies revealed no adverse effects on vital organs, body weight, or serum biochemical parameters, highlighting the safety profile of *A. glandulosa* [146] (Table 6).

### 5.10. Anti-Diarrheal Effects

Diarrhea is a common gastrointestinal condition that is characterized by increased stool frequency, fluidity, and volume. It is frequently accompanied by fecal urgency and incontinence. There are a wide variety of etiological causes that can lead to this condition, including microbial infections, certain medications, toxic substances, gastrointestinal illnesses, and the consumption of substances that are not readily absorbed by the body. It is estimated that diarrhea accounts for approximately 3.6% of the overall epidemic burden and is responsible for nearly 8% of all pediatric deaths [309,310]. It is a primary source of morbidity and mortality in children under the age of five, resulting in an estimated 2.5 billion incidents and 1.5 million fatalities yearly. This is especially true in countries with low and middle incomes, where it is responsible for more than 21% of deaths among children under the age of five. In response, the WHO Diarrheal Disease Control Program promotes the use of traditional medicines to control and treat diarrhea. Several studies have linked the decline of medicinal plant knowledge to oral transmission, inadequate resource management, a lack of understanding of herbal medicine, and a lack of interest among younger generations [311,312].

To validate the ethnomedicinal use of *A. laxiflora* leaves in treating gastrointestinal disorders in Cameroon, Wansi et al. conducted an in vivo investigation to evaluate the anti-diarrheal effects of aqueous and methanol leaf extracts at doses of 125, 250, and 500 mg/kg. The extracts were investigated using rat models of diarrhea induced by *S. flexneri* (infectious), magnesium sulfate (osmotic), and castor oil (secretory). Among these, the methanol extract showed significant anti-diarrheal effects across all models. Notably, in the osmotic diarrhea model, the extract significantly prolonged the latency period, with the 250 mg/kg dose producing the highest delay (71.6% increase), indicating potent inhibitory effects on fluid secretion and intestinal hypermotility [244].

In a similar context, *A. cordifolia* is traditionally administered as maceration or decoction to treat diarrhea and amoebic dysentery [95]. Pharmacologic studies have substantiated these ethnomedicinal practices. Agbor et al. assessed the anti-diarrheal potential of ethanolic leaf extract of *A. cordifolia* in a castor oil-induced diarrhea model in mice. The extract showed dose-dependent protection, with maximum efficacy at 800 mg/kg [313]. Furthermore, Emudainohwo (2015) evaluated the potential of aqueous and ethanol bark extracts of *A. cordifolia* in models of castor oil-induced diarrhea, entero-pooling, and gastrointestinal motility. Both extracts showed significant anti-diarrheal activity in a dose-dependent manner at 200 and 400 mg/kg, supporting their potential to reduce intestinal fluid accretion and motility [314] (Table 6).

### 5.11. Immunomodulatory Effects

The immune system acts as the body’s natural defense against foreign agents and a wide range of pathogens. A properly functioning immune response is crucial for controlling and preventing diseases, especially those that weaken immune responses, such as Ebola virus infections and *HIV/AIDS* [315]. Recent research has shown that both crude extracts and pure compounds from *Alchornea* species have notable immunomodulatory effects.

Flavonoids isolated from *A. floribunda* **249**, **253–254**, **262**, **266**, and **269** have shown immunomodulatory effects, as evaluated by flow cytometry [158]. Additionally, ethyl acetate and acetone extracts of *A. cordifolia* have exhibited significant in vitro immunostimulatory effects. These extracts promoted the proliferation of splenocytes and thymocytes, enhanced intracellular killing capacity (IKC), and notably reduced nitric oxide (NO) production [91]. Furthermore, Kouakou et al. reported that *A. cordifolia* leaf extract increased cytokine production by 50-fold in vitro, indicating substantial potential for immune system regulation [9] (Table 6).

### 5.12. Other Effects

Quercetin (**261**), Quercetin-3-*O*-*β*-D-glucopyranoside (**262**), Quercetin-3-*O*-*α*-L-rhamnopyranoside (**266**), Quercetin-3-*O*-*β*-D-galactopyranoside (**268**), Quercetin-3-*O*-*α*-D-arabinopyranoside (**269**), and alchorneine (**346**) from *A. cordifolia*, *A. laxiflora*, *and A. glandulosa* showed in vitro anti-sickling and anti-trypanosomal effects [97,163,316]. Protocatechuic acid (**225**), rutin (**264**), and pterogynidine (**335**) from *A. glandulosa* inhibited angiogenesis in human umbilical vein endothelial cells (HUVECs) [96,168].

In a murine study, the aqueous extract of *A. cordifolia*, administered orally at doses of 100–400 mg/kg, significantly (*p* < 0.05) reduced the duration of immobility in the forced swim test, suggesting anti-stress and anti-fatigue effects [317]. Regarding *A. laxiflora*, a decoction at 60 mg/kg protected all mice against *N*-methyl-D-aspartate (NMDA)-induced turning behavior (*p* < 0.001). At 120 mg/kg, it protected 75% of mice against seizures induced by strychnine (STR) (*p* < 0.01); however, it did not affect convulsions caused by pentylenetetrazol (PTZ). Moreover, in a diazepam-induced sleep test, *A. laxiflora* did not significantly alter sleep duration compared with the standard, indicating limited sedative effects [115]. Likewise, a hydroethanolic extract of *A. cordifolia* leaves showed dose-dependent (200 and 400 mg/kg) and statistically significant (*p* < 0.001) antidepressant-like effects in the same behavioral test. Additionally, sub-effective doses of the conventional antidepressant imipramine (5 mg/kg) and fluoxetine (5 mg/kg), when given orally alongside a low dose of the extract (25 mg/kg), produced a synergistic antidepressant-like effect, pointing to an interaction with monoaminergic pathways [318]. *A. laxiflora* demonstrated neuropharmacological potential beyond its hematological applications. A recent mechanistic study suggested that its antidepressant effects might be enhanced by modulating the PI3K-Akt and MAPK signaling pathways [319].

A recent clinical study assessed the effectiveness, safety, and tolerability of Mojeaga. This polyherbal formulation includes *A. cordifolia* as an adjunct to traditional oral iron therapy for the treatment of anemia in obstetric patients [320]. Additionally, the anxiolytic effects of *A. cordifolia* were examined by observing the impact of its aqueous leaf extract on spontaneous movements and exploratory behavior in laboratory mice. The findings indicate a central nervous system depressant effect, likely with anxiolytic properties [321]. In another study, polyphenol and tannin extracts from traditional antidiarrheal medicinal plants, including *A. cordifolia*, demonstrated significant anti-spasmodic effects, supporting its ethnomedicinal use [216].

Mpiana et al. investigated 30 aqueous and ethanol extracts from 13 Congolese plant species for anti-sickle cell activity [322]. A combination extract (Combo A), made by mixing the leaves of *A. laxiflora* and *C. ferruginea*, showed significant anti-sickling effects in vitro. Tested at concentrations of 4, 6, 8, and 10 mg/mL, the extract demonstrated inhibitory anti-sickling effects of 91.6%, 97.9%, 98.8%, and 93.7%, respectively. These results remained consistent across different combination ratios (1:1, 1:2, and 2:1), indicating a strong and synergistic effect [323]. Similarly, in an ethnobotanical survey, 23 plant species traditionally used to treat sickle cell disease in Kikwit, Democratic Republic of the Congo (DRC), were documented. Eighteen of these species exhibited significant anti-sickling effects, with a normalized rate of at least 70%, supporting their traditional medicinal use [324]. Further pharmacological studies have explored the endocrine and reproductive effects of *A. cordifolia*. Ebenyi et al. evaluated the impact of leaf extract on fertility-related hormones in albino rats, suggesting potential endocrine-modulating properties [325]. Similarly, in reproductive toxicology research, Atoe et al. examined the teratogenic effects of methanol extract from *A. cordifolia* leaves in pregnant Wistar rats. While the extracts were not highly toxic, *A. cordifolia* was associated with teratogenic effects, including alterations in anogenital distance, crown-rump length, and placental weight and volume [326]. According to Lopes et al., significant decreases in proliferation and invasion capacity, and a marked increase in apoptosis, as assessed by bromodeoxyuridine incorporation, the double-chamber assay, and terminal deoxynucleotidyl transferase nick end labeling, respectively, have been observed. It also led to a drastic reduction in the number of capillary-like structures formed when HUVECs were cultured on growth factor-reduced Matrigel-coated plates [168]. Similarly, these findings emphasize the antiangiogenic potential of AGF and support its therapeutic use for disorders characterized by excessive angiogenesis, such as chronic inflammation and tumor growth [96]. According to Barrosa et al., alchornedine was effective against both clinical forms of *T. cruzi* and could serve as a scaffold for future development studies targeting American trypanosomiasis [97]. The results indicated that *A. laxiflora* exhibited a significant (*p* ≤ 0.05) neuromuscular inhibitory activity, evidenced by increased time for animals to hold the straightened wire with their hind limbs in the traction test [327]. This study also showed that the polyphenol-rich fraction of *A. cordifolia* protected against sodium arsenite-induced infertility in male rats by inhibiting oxidative and apoptotic mechanisms [328]. Methanol extracts of *A. cordifolia* leaves and their sub-fractions showed >70% suppression of HbSS erythrocyte sickling. The purified compound demonstrated an 87.2 ± 2.39% significant anti-sickling activity and 93.1 ± 2.69% erythrocyte sickling-inhibition at 0.4 mg/mL [163]. The results indicated that oral BPA administration caused obesity, as shown by increased body weight and a higher Lee’s Index compared with the standard control (*p* < 0.05). Furthermore, BPA treatment altered serum biochemical markers and lipid-regulating enzymes relative to the standard control [329]. The study concluded that high-dose administration of *A. cordifolia* may damage the kidneys of adult Wistar rats. However, findings indicated no differences in renal cortical cytoarchitecture between the test groups and the controls [330]. This study clarifies potential mechanisms behind the neuroprotective effects of *A. cordifolia* and provides empirical evidence supporting the traditional use of these herbs in folk medicine. In addition, these roots could serve as sources of nutraceuticals and functional foods for the prevention and treatment of neurodegenerative diseases, particularly amnesia [331] (Table 6).

**Table 6:** Summary of the anti-malarial, anti-plasmodial, anti-diabetic, anti-ulcerogenic, anti-diarrheal, immunomodulatory, and other effects of extracts and pure compounds from the genus *Alchornea*.

Name of Plant	Extracts/Pure Compounds	Study	Model	MIC/Doses	IC_50_/EC_50_/Results	Reference
*** Anti-malarial and anti-plasmodial effects ***						
*A. cordifolia*	MLExt, EaLExt	In vitro	*P. falciparum*	0.005 mg/mL or 5.0 g/mL	12.2 ± 0.8 µg/mL, 12.9 ± 1.2 µg/mL	[147]
*A. cordifolia*	AqFExt	In vitro	Tetrazolium-based colorimetric selective assay	0–1000.0 mg/mL	4.9 mg/mL	[297]
*A. cordifolia*	MLExt	In vitro	Chloroquine-sensitive Ghanaian strain of *P. falciparum*	0–1000.0 mg/mL	2.8 mg/mL	[89]
*A. cordifolia*	AqLExt, ELExt, ChLExt, PEExt	In vitro	Fcm-29 chloroquine resistant	0.01–100.0 mg/mL	0.2–0.5 μM	[165]
*A. cordifolia*	AqLExt, ELExt, PEExt	In vitro	FcM1, and FcM-29 chloroquine resistant	0.01–1000.0 mg/mL	0.35 to 43.40 μg/mL	[332]
*A. floribunda*	AqRExt, AqLExt	In vitro	K1 strain of *P. falciparum*	5.0–10.0 μg/mL	19.65, 37.26, 20.80 µg/mL and >64 µg/mL	[33]
*A. laxiflora*	MLExt, ChLExt	In vivo	*P. berghei*-infected Swiss albino mice	200.0–600.0 mg/kg	The results showed significantly greater (*p* < 0.05) prophylactic, suppressive, and curative anti-malarial effects	[298]
*A. laxiflora*	AqLExt, ELExt, BuLExt, EaLExt, ChLExt, PELExt	In vitro	CQ-sensitive Pf-3D7, CQ-resistant Pf INDO	0–100.0 mg/mL	40.17 ± 0.78 μg/mL and 38.44 ± 0.89 μg/mL	[132]
*A. laxiflora*	ELExt	In vivo	*P. berghei*-infected Swiss albino mice	200.0–600.0 mg/kg	The leaf extract (200–600 mg.kg^−1^, p.o.), with an LD_50_ of 2236 mg.kg^−1^, was antimalarial in suppressive, prophylactic, and curative tests against *P. berghei* infection.	[132]
*A. laxiflora*	AqRExt, ERExt, BuRExt, EaRExt, ChRExt, PERExt	In vivo	*P. berghei*-infected Swiss albino mice	200.0–600.0 mg/kg	The extracts (75–225 mg/kg, p.o.), with an LD_50_ of 748.33 mg/kg, exhibited significant (*p* < 0.05–0.001) antimalarial activity in suppressive, prophylactic, and curative tests against *P. berghei* infection.	[131]
*** Anti-diabetic effects ***						
*A. cordifolia*	BuExt	In vivo	Wistar rats	200.0–800.0 mg/kg	The extract efficiently reduced blood glucose levels, showing comparable effectiveness to glibenclamide (10 mg/kg).	[301]
*A. cordifolia*	EExt	In vivo	Wistar rats	250.0–500.0 mg/kg/day	*A. cordifolia* leaf extract increased aortic elastic fibers in rats, suggesting improved vascular elasticity.	[333]
*A. laxiflora*	MLExt	In vivo	Rats	500.0 mg/kg	The results showed that 500 mg/kg lowered blood glucose in diabetic rats (~146→88 mg/dL) and improved metabolic markers.	[334]
*A. laxiflora*	MExt	In vitro	*α*-amylase inhibitory	31.3–1000.0 μg/mL	295.60 μg/mL	[269]
*** Anti-HIV effects ***						
*A. cordifolia*	**231**	In vitro	HIV-1 integrase strand transfer assay	20.0 μM	3.7 nM	[181]
*A. cordifolia*	AqExt	In vitro	Molt-4 and Molt-4/HIV, HIV reverse transcriptase (RT) assay	0.4 mg/mL	0.01–0.03 mg/mL	[305]
*A. laxiflora*	**151**, **152**, **231**, **313**, **314**	In vitro	HIV-1 integrase strand transfer assay	20.0 μM	3.7 nM-20 nM	[181]
*A. laxiflora*	HexRExt, ChRExt, EaRExt, MRExt, ERExt, AqRExt, MStBExt	In vitro	HIV-1 integrase strand transfer assay	25.0 μg/mL	8.5 ng/mL	[181]
*A. laxiflora*	**142**, **143**, **147**, **149**, **159**	In vitro	HIV-1 integrase strand transfer assay	20.0 μM	90.23 μM and more than 100 μM	[241]
*A. laxiflora*	MStBExt	In vitro	HIV-1 integrase strand transfer assay	50.0 μg/mL	0.21 ng/mL	[241]
*** Anti-ulcerogenic effects ***						
*A. castaneifolia*	Aq/ELExt, Aq/EBExt, EFF	In vivo	Wistar rats	100.0–1000.0 mg/kg	The hydroalcoholic leaf extract and flavonoid-enriched fraction significantly enhanced ulcer healing.	[52]
*A. cordifolia*	polyherbal formulations (EXR-HF1, EXR- HF2, EXRHF3 and EXR-HF4)	In vivo	Wistar rats	10.0–5000.0 mg/kg	The formulations significantly inhibited ulcer formation (*p* < 0.05), with EXR-HF4 showing the strongest anti-ulcer effect.	[308]
*A. glandulosa*	MLExt	In vivo	Rats	250.0–1000.0 mg/kg	A single oral dose of *A. glandulosa* (250 mg/kg/day) significantly enhanced gastric epithelial cell production, accelerating ulcer healing.	[146]
*A. triplinervia*	MExt	In vivo	Mice, Wistar rats	250.0–1000.0 mg/kg	*A. triplinervia* showed consistent gastroprotection (85–90%) in ethanol and HCl/ethanol ulcer models at 500–1000 mg/kg of methanol extract.	[249]
***Anti-diarrheal effects***						
*A. cordifolia*	AqStBExt, EStBExt	In vivo	Wistar rats	200.0–400.0 mg/kg	*A. cordifolia* extracts showed dose-dependent anti-diarrheal activity at 200–400 mg/kg.	[221]
*A. cordifolia*	EExt	In vivo	Mice	25.0–800.0 mg/kg	The extract showed dose-dependent protection, with maximum efficacy at 800 mg/kg.	[313]
*A. laxiflora*	AqLExt, MLExt	In vivo	Rats	125.0–500.0 mg/kg	The extract significantly prolonged latency, with 250 mg/kg producing the greatest delay (71.6%), indicating strong inhibitory effects.	[244]
*** Immunomodulatory effects ***						
*A. cordifolia*	AqLExtPS	In vitro	Increase in the cytokine production	10.0–250.0 μg/mL	*A. cordifolia* leaf extract boosted cytokine production 50-fold, showing strong immune-regulatory potential.	[9]
*A. cordifolia*	AcLExt, EaLExt	In vitro	Increase in the proliferation of splenocytes and thymocytes	10.0–250.0 μg/mL	*A. cordifolia* leaf fractions boosted lymphocyte proliferation and macrophage activity while modulating nitric oxide.	[335]
*** Other effects ***						
*A. cordifolia*	ELExt	In vivo	Wistar rats	50.0–1000.0 mg/kg	*A. cordifolia* extract (500–1000 mg/kg) reduced BPA-induced obesity in rats.	[329]
*A. cordifolia*	EaRExt	In vitro	*T. pachysiphon*	-	*A. cordifolia* methanol extracts inhibited anthracnose spore germination and growth.	[336]
*A. cordifolia*	AqLExt	In vivo	Wistar rats	100.0–1500.0 mg/kg	*A. cordifolia* extract mildly affected kidney weight and urea at >500 mg/kg without altering structure or creatinine.	[330]
*A. cordifolia*	MLExt	In vivo	Wistar rats	200.0 mg/kg	*A. cordifolia* extract caused fetal mortality and deformities at >50 mg/kg in pregnant rats.	[326]
*A. cordifolia*	**266**	In vitro	Emmel test	0.4 mg/mL	Quercitrin from *A. cordifolia* inhibited up to 93% of sickling and HbS polymerization.	[163]
*A. cordifolia*	AqLExt, MLExt, DLExt	In vitro	Emmel test	1 mg/mL	91.4%	[163]
*A. cordifolia*	MLExt	In vivo	Preeclamptic-Induced Wistar rats	50.0–200.0 mg/kg	*A. cordifolia* extract normalized sodium, calcium, and magnesium in preeclamptic rats.	[337]
*A. cordifolia*	HexLExt, ChLExt, EaLExt, MLExt	In vivo	Rats	100.0–150.0 µg/kg	*A. cordifolia* polyphenols reversed arsenite-induced infertility in male rats.	[328]
*A. cordifolia*	AqLExt	In vitro	Emmel test	11.0 μg/mL	*A. cordifolia* extracts show strong in vitro antisickling activity.	[324]
*A. cordifolia*	AqLExt	In vitro	Emmel and hemolysis tests	50.0 µg/mL	*A. cordifolia* normalized ≥70% of sickle red blood cells.	[338]
*A. cordifolia*	MLExt	In vitro	HEp-2	10.0 µg/mL	5.64 ± 1.16 µg/mL	[339]
*A. cordifolia*	AqLExt, EaLExt	In vivo	Albino rats	200.0–800.0 mg/kg	Leaf extract altered fertility hormone levels in albino rats, suggesting endocrine-modulating potential.	[325]
*A. cordifolia*	AqLExt	In vivo	Albino mouse	2500.0 mg/kg	The findings indicate a central nervous system depressant effect, likely with anxiolytic properties	[321]
*A. cordifolia*	AqLExt	In vivo	Picrotoxin-treated mice	100.0–400.0 mg/kg	*A. cordifolia* extract (100–400 mg/kg) reduced forced swim immobility, showing anti-stress effects.	[317]
*A. cordifolia*	Aq/ELExt	In vivo	Wistar rats	250.0–500.0 mg/kg	*A. cordifolia* leaf extract increased aortic elastic fibers in rats, suggesting improved vascular elasticity.	[333]
*A. floribunda*	MLExt	In vitro	HEp-2	10.0 µg/mL	75.62 ± 3.38 µg/mL	[339]
*A. glandulosa*	**346**	In vitro	MTT assay	300.0 μg/mL	27–93 µg/mL	[97]
*A. glandulosa*	**225**, **264**	In vitro	HUVEC (human umbilical vein endothelial cells), MTT assay	-	*A. glandulosa* fraction shows in vitro antiangiogenic activity.	[96]
*A. glandulosa*	EaLExt	In vitro	MTT assay	50.0–400.0 μg/mL	Cell viability (96.58–3.88%) was observed with EaLExt at 50 μg/mL	[96]
*A. glandulosa*	**335**	In vitro	HUVEC (human umbilical vein endothelial cells), MTT assay	8.0–128.0 μM	Pterogynidine from *A. glandulosa* shows in vitro anti-angiogenic activity.	[168]
*A. glandulosa*	AqLExt, ELExt, PELExt	In vitro	Neonate larvae of *Spodoptera frugiperda*	23.0–95.0 μg/mL	*A. glandulosa* showed IC_50_ ~38–54 µg/mL.	[154]
*A. laxiflora*	ELExt	In vivo	Cockerel chickens	100.0–200.0 mg/kg	*A. laxiflora* leaf extract (100–200 mg/kg) protects against lead-induced neurodegeneration in cockerels.	[340]
*A. laxiflora*	AqLExt	In vitro	Mushroom tyrosine enzyme	50.0 µg/mL	66.3 μg/mL	[341]
*A. laxiflora*	MLExt	In vitro	Emmel test	2.0–8.0 mg/mL	Results were consistent across all ratios, indicating a synergistic effect.	[323]
*A. laxiflora*	AqLExt, MLExt	In vivo	Adult albino mice	100.0–1600.0 mg/kg	*A. laxiflora* leaves showed dose-dependent anxiolytic activity in mice	[342]
*A. laxiflora*	AqLExt	In vivo	Adult male mice	60.0–120.0 mg/kg	*A. laxiflora* protected against NMDA and strychnine seizures but had no effect on PTZ convulsions or sedation.	[115]

AqFExt: Aqueous fruit extract; MLExt: Methanol leaf extract; AqLExt: Aqueous leaf extract; ELExt: Ethanol leaf extract; ChLExt: Chloroform leaf extract; PEExt: Petroleum ether extract; AqRExt: Aqueous root extract; BuLExt: *n*-Butanol leaf extract; EaLExt: Ethyl acetate leaf extract; PELExt: Petroleum ether leaf extract; ERExt: Ethanol root extract; BuRExt: *n*-butanol root extract; EaRExt: Ethyl acetate root extract; ChRExt: Chloroform root extract; PERExt: Petroleum ether root extract; BuExt: Butanol extract; EExt: Ethanol extract; MExt: Methanol extract; AqExt: Aqueous extract; HexRExt: Hexane root extract; MRExt: Methanol root extract; MStBExt: Methanol stem bark extract; Aq/ELExt: Aqueous and ethanol leaf extracts; Aq/EBExt: Aqueous and ethanol bark extract; EFF: Enriched flavonoid fraction; AqStBExt: Aqueous stem bark extract; EStBExt: Ethanol stem bark extract; AqLExtPS: Polysaccharide from ethanol fraction of aqueous leaf extract; AcLExt: Acetone leaf extract; DLExt: DCM leaf extract; HexLExt: Hexane leaf extract.

3-*O*-Acetyl-erythrodiol (**142**); 3-*O*-Acetyl-aleuritolic acid (**143**); Friedelin (**147**); Friedelane-3-one-28-al (**149**); 3-*O*-Acetyl-oleanolic acid (**151**); 3-*O*-Acetyl-ursolic acid (**152**); Stigmasterol (**159**); Protocatechuic acid (**225**); Methyl gallate (**231**); Rutin (**264**); Quercetin-3-*O*-*a*-L-rhamnopyranoside (**266**); Ellagic acid (**313**); 3-*O*-Methylellagic acid (**314**); Pterogynidine (**335**); Alchornedine (**346**).

## 6. Toxicology

Toxicological data are currently available for only a limited number of species within the genus *Alchornea*, and most existing studies have primarily evaluated the acute, sub-acute, and sub-chronic toxic effects of crude plant extracts. While these studies provide a basic understanding of the immediate and short-term safety profiles of these extracts, they are insufficient to fully assess the long-term risks associated with the medicinal use of *Alchornea* species. Therefore, to develop a thorough and reliable safety profile and to encourage responsible, safe therapeutic use of these plants, more detailed toxicological evaluations are urgently needed. These should include, but are not limited to, chronic toxicity studies to examine the effects of prolonged exposure, as well as mutagenicity and teratogenicity studies to assess potential genetic and developmental risks.

Moreover, it is crucial to comprehensively organize and assess toxicological data for purified active compounds isolated from *Alchornea* species, as the pharmacological and toxicological profiles of these individual compounds can vary significantly from those seen with crude plant extracts. Such differences may be due to variations in bioavailability, synergistic or antagonistic interactions among multiple constituents, or the existence of previously unknown bioactive metabolites. Although current evidence indicates that *Alchornea* extracts have relatively low toxicity at standard therapeutic doses, caution should still be exercised with long-term use or high-dose administration, especially in the absence of robust clinical data from human studies. Until such data are available, conclusions from preclinical studies should be drawn carefully, and additional research involving human participants is highly recommended to ensure safe and effective clinical use.

The acute toxicity of *A. laxiflora* hexane and methanol leaf extract was assessed by Farombi et al. in Wistar rats. No mortality was observed following oral administration at 100–5000 mg/kg, with an LD_50_ above 5000 mg/kg, indicating low acute toxicity [105]. Traore et al. reported similar results in a mouse model. No mortality was observed following a single oral administration, with LD_50_ values above 5000 mg/kg and 1000 mg/kg [343]. Additionally, the Aqueous and ethyl acetate fractions of *A. triplinervia* were investigated for toxicity using the *Artemia salina* brine shrimp lethality assay, yielding LC_50_ values of 110 μg/mL and 92 μg/mL, respectively [250]. Adedapo et al. reported that the oral administration of *A. cordifolia* aqueous leaf extract in rats significantly elevated ALT and AST levels, suggesting hepatocellular damage, while reductions in WBC and RBC counts indicated a risk of anemia and hematotoxicity effects [344]. Furthermore, a Sub-Acute toxicity investigation of the *A. cordifolia* ethanolic leaf extract showed no significant effects on hematological parameters, organ weights, or renal function at doses up to 2000 mg/kg. However, histopathological liver damage, including eosinophilia, pyknosis, and lymphocytic infiltration, was observed at higher doses, demonstrating dose-dependent hepatotoxicity [345,346]. Similarly, the essential oil from *A. cordifolia* leaves exhibited significant toxicity in the brine shrimp lethality assay, with an LC_50_ of 34.74 μg/mL [281].

Acute oral administration of *A. cordifolia* aqueous leaf extract was non-lethal in mice at doses up to 32 g/kg; however, doses above 4 g/kg induced weight loss, CNS sedation, and reduced pain sensitivity [231]. Additionally, no mortality was observed, though elevated hepatic toxicity at high doses indicated caution with oral administration [347]. A 30-day oral administration of *A. cordifolia* aqueous leaf extract at 250 mg/kg in Wistar rats resulted in diffuse glomerulonephritis, renal cortical disruption, and enlarged Bowman’s spaces, indicating nephrotoxic effects [348]. Esosa et al. demonstrated that initial treatment with an *A. laxiflora* hexane extract (10 mg/kg) significantly reduced sodium arsenate-induced hepatic toxicity in Wistar rats, resulting in higher liver enzyme levels. However, post-treatment was less effective, signifying that the extract might have prophylactic potential [287]. The hydroethanolic and methanol leaf extracts of *A. cordifolia* exhibited dose-dependent toxicity in mice. In comparison, no mortality was observed up to 4000 mg/kg; higher doses induced renal biomarkers, behavioral changes, and elevated hepatic enzymes, indicating hepatotoxic and nephrotoxic effects [318,349]. The methanolic leaf extract of *A. laxiflora* revealed significant toxicity in the brine shrimp lethality assay with an LC_50_ of 142.40 μg/mL [269]. Similarly, Osabiya et al. reported potent toxicity effects for both aqueous and ethanol leaf extracts, with LC_50_ values of 41.01 μg/mL and 8.91 μg/mL, respectively [242].

Sub-acute toxicity studies exhibited no significant changes in renal parameters or organ weights up to 0.5 g/kg; however, hepatic degeneration was observed at 0.75 g/kg of ethanol leaf extract [350]. Oral administration of aqueous *A. cordifolia* leaf extract reduced locomotion, changed excreta consistency, and sensory responses in mice in a dose-dependent manner, with LD_50_ values of 8.6 g/kg (males) and 3.8 g/kg (females) [183]. Okokon et al. evaluated the toxicity of ethanolic extracts from *A. laxiflora* leaves and roots *via* intraperitoneal administration in mice. The LD_50_ values were 2236 mg/kg for leaves and 748.33 mg/kg for roots, demonstrating toxicity effects at higher doses [131,132].

The acute toxicity of the methanol leaf extract of *A. floribunda* was assessed using Lorke’s method, with graded doses administered in vivo. Observations over 24 h and 14 days showed a safety margin below 567.70 mg/kg intraperitoneally and 2000 mg/kg orally. Thus, lower doses are recommended for in vivo studies [29,31]. The ethanol extract of *A. cordifolia* exhibited a high safety margin, with an LD_50_ of 14.8 g/kg, which is well above the WHO toxicity threshold of 2.0 g/kg, indicating potential safety for therapeutic use [351]. The acute toxicity of *A. laxiflora* aqueous and methanol leaf extracts was assessed in mice. The aqueous extract was found to be safe up to 1600 mg/kg when administered orally or intraperitoneally. In contrast, the methanol extract was tolerated at doses of up to 1600 mg/kg orally and 400 mg/kg intraperitoneally [342,352,353]. Essential oils from *A. laxiflora* leaves administered orally at doses of 100, 200, and 400 mg/kg in rats induced liver hypertrophy, reduced bilirubin levels, and serum ALT, demonstrating hepatotoxicity effects at higher doses [149].

Sub-chronic oral administration of an aqueous extract of *A. cordifolia* leaves for up to 60 days showed no significant toxicity in rats, with no adverse effects on body weight or vital organ function at doses of 100–400 mg/kg [126]. Similarly, acute and subacute studies reported no mortality at 2000 mg/kg, with an LD_50_ above the threshold [176]. Additionally, an acute toxicity study of the ethanolic leaf extract of *A. cordifolia* in Wistar rats, conducted via the limit dose method, reported no signs of toxicity at an LD_50_ of 5000 mg/kg [354]. Furthermore, this study investigated the acute toxicity of the ethanolic leaf extract of *A. cordifolia* to determine the LD_50_. Findings revealed that a dose of 1500 mg/kg did not cause mortality; however, a dose of 2000 mg/kg caused 100% mortality [355].

The genus *Alchornea* exhibits a favorable safety margin in acute toxicity studies; however, a critical assessment of its phytochemical constituents raises concerns about certain alkaloids. In particular, the presence of yohimbine in species such as *A. floribunda* and *A. hirtella* warrants caution. Yohimbine is a competitive antagonist of *α*_2_-adrenergic receptors and can cause dose-dependent adverse effects in humans, including hypertension, tachycardia, tremors, and anxiety. Various *Alchornea* species are traditionally used topically for wound healing and dermatological conditions. The presence of alkaloids, condensed tannins, and terpenoids raises the possibility of contact dermatitis and irritation, mainly upon prolonged exposure. For topical applications, which are often used in ethnomedicine, dermal safety must also be carefully addressed. Although patch-testing data for most *Alchornea* species remain limited, the presence of bioactive diterpenes and polyphenols suggests a potential risk of both irritant contact dermatitis and Type-IV hypersensitivity in sensitive individuals. Thus, future formulation development and pharmacovigilance studies should prioritize dermal sensitization assays to ensure the safety of topical *Alchornea*-based preparations.

## 7. Conclusions and Future Perspectives

**Figure 13:**
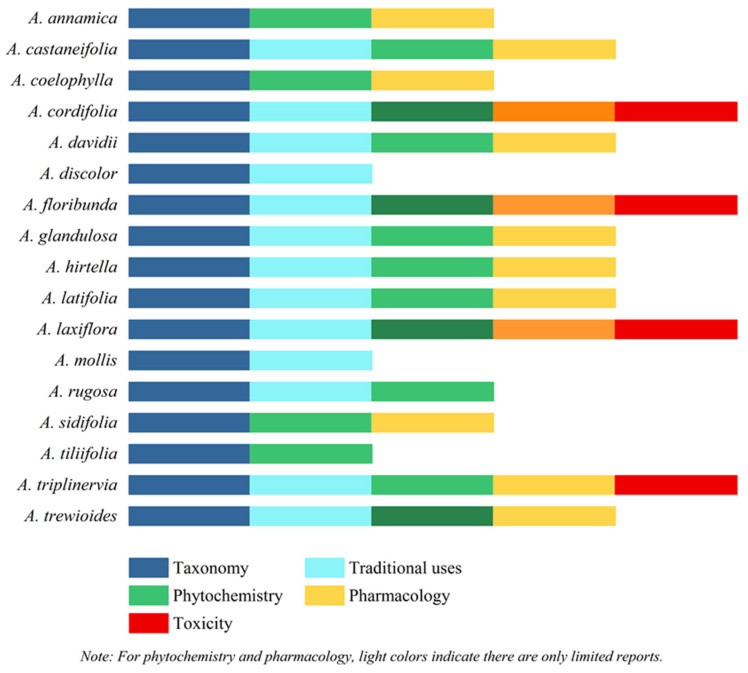
Critical overview of research on the *Alchornea* species.

In conclusion, although research on *Alchornea* species has increased significantly in recent years, to our knowledge, there has been no comprehensive review of recent advances in the genus *Alchornea*. This review summarizes experimental evidence on its taxonomy, traditional uses, phytochemistry, pharmacological potential, and toxicology, and highlights the limitations of existing data while suggesting future research directions (Figure 13). The genus *Alchornea*, which includes a diverse group of species within the Euphorbiaceae family, is known for its rich ethnomedicinal importance and bioactive chemical components. In fact, *Alchornea* species are commonly used in traditional medicine to treat various conditions, including inflammation, colds, pain, ulcers, rheumatism, arthritis, respiratory issues, dysentery, diarrhea, eczema, hepatitis, sexually transmitted diseases, malaria, and anemia. However, the pharmacological potential and mechanisms underlying many of these traditional uses are still not fully supported by modern preclinical and clinical research. Extracts and pure compounds derived from different *Alchornea* species, especially from *A. cordifolia*, *A. laxiflora*, and *A. floribunda*, have demonstrated a wide range of pharmacological effects. Mechanistic statistics are especially lacking for the anti-inflammatory, hepatoprotective, anti-cancer, and immunomodulatory effects generally attributed to these species. Future investigations should focus on key signaling pathways, such as NF-κB, Nrf2/KEAP1, MAPK, PI3K/Akt, JAK/STAT, and apoptotic regulators, as well as metabolic targets, including PPARs, AMPK, and glucose transporters, to strengthen the pharmacological evidence. For antimicrobial and antimalarial activities, studies should explore DNA/RNA interference pathways, parasite-specific enzymes, redox-modulating targets, and membrane integrity assays. To date, phytochemical studies have identified **396** compounds across various *Alchornea* species. Phytochemical and pharmacological research has been conducted on approximately 17 *Alchornea* species. Nonetheless, these findings indicate that further research is needed to assess the efficacy and safety of the largely understudied *Alchornea* genus. Toxicological data are limited, with only a few species, including *A. cordifolia*, *A. laxiflora*, *A. floribunda*, and *A. triplinervia*, having undergone initial toxicity evaluations. However, existing studies are insufficient to establish comprehensive safety profiles.

Future research should prioritize the valorization of the genus *Alchornea* by developing standardized topical and oral formulations that harness its approved anti-inflammatory and antimicrobial effects. To translate the pharmacological effects of *Alchornea* species into clinical practice, a strategic focus on modern plant valorization is required. Based on current pharmacological evidence, particularly robust anti-inflammatory, antimicrobial, and wound healing properties, there is a strong rationale for developing standardized phytomedicines and prioritizing topical formulations to treat dermatological conditions, burns, and microbial skin infections, leveraging the high flavonoid and tannin content of leaf extracts. Simultaneously, developing oral delivery systems is warranted to manage gastrointestinal disorders and inflammation while mitigating potential gastric irritation.

To improve translational potential, future research should prioritize: comprehensive quantitative ethnobotanical certification (1), integrated taxonomy with voucher deposition and molecular barcoding (2), extensive metabolomic and structural elucidation techniques (LC-MS/MS, 1D/2D NMR) combined with bioassay-guided fractionation and SAR analyses (3), and standardized toxicology testing in line with international protocols (4). Integrating traditional medicinal knowledge with modern research can support the validation and safe application of therapeutic agents derived from the genus *Alchornea*.

## Figures and Tables

**Table 1:** List of all accepted botanical names and synonyms of reported *Alchornea* species.

Sr. No.	Accepted Botanical Name	Synonyms Used in the Referenced Articles
1	*Alchornea annamica* Gagnep.	
2	*Alchornea castaneifolia* (Humb. and Bonpl. ex Willd) A. Juss.	
3	*Alchornea coelophylla* Pax and K. Hoffm.	
4	*Alchornea cordifolia* (Schumach.) Mull. Arg.	
5	*Alchornea davidii* Franch.	
6	*Alchornea discolor* Poepp.	
7	*Alchornea floribunda* Mull. Arg.	
8	*Alchornea glandulosa* Poepp.	
9	*Alchornea hirtella* Benth.	
10	*Alchornea latifolia* Sw.	
11	*Alchornea laxiflora* Pax and K. Hoffm.	
12	*Alchornea mollis* Mull. Arg.	
13	*Alchornea rugosa* Mull. Arg.	*Alchornea javanensis* (Blume) Mull. Arg.
14	*Alchornea sidifolia* Mull. Arg.	
15	*Alchornea tiliifolia* (Benth) Mull. Arg.	
16	*Alchornea trewioides* Mull. Arg.	
17	*Alchornea triplinervia* (Spreng) Mull. Arg.	

## Data Availability

No new data were created or analyzed in this study. Data sharing is not applicable to this article.

## Abbreviations

The following abbreviations are used in this manuscript:

ABTS	2,2′-Azino-bis(3-ethylbenzothiazoline-6-sulfonic acid)
AIDS	Acquired immunodeficiency syndrome
ALP	Alkaline phosphatase
ALT	Alanine aminotransferase
AST	Aspartate aminotransferase
CAT	Catalase
CCl_4_	Carbon tetrachloride
CNS	Central nervous system
DNA	Deoxyribonucleic acid
DPPH	2,2-diphenyl-1-picrylhydrazyl
DRC	Democratic Republic of the Congo
FRAP	Ferric reducing antioxidant power
FTC	Ferric thiocyanate
GC-MS	Gas chromatography-mass spectrometry
GGT	Gamma-glutamyl transferase
GSH	Glutathione
GST	Glutathione *S*-transferase
H_2_O_2_	Hydrogen peroxide
HAT	Human African trypanosomiasis
HEK	Human embryonic kidney
HeLa	Human cervical carcinoma
HIV	Human immunodeficiency virus
HL-60	Human promyelocytic leukemia cell line
HR-MS	High-resolution mass spectrometry
HUVECs	Human umbilical vein endothelial cells
IC_50_	Half-maximal inhibitory concentration
IKC	Intracellular killing capacity
LC_50_	Lethal concentration 50%
LC-MS/MS	Liquid chromatography-tandem mass spectrometry
LD_50_	Lethal dose 50%
LOX	Lipoxygenase
LPS	Lipopolysaccharide
MAPK	Mitogen-activated protein kinase
MCF-7	Estrogen receptor-positive breast cancer
MDA-MB-231	Triple-negative breast cancer
MDR	Multidrug-resistant
MIC	Minimum inhibitory concentration
MTT	(3-(4,5-dimethylthiazol-2-yl)-2,5-diphenyltetrazolium bromide)
NMDA	*N*-methyl-D-aspartate
NMR	Nuclear magnetic resonance
NO	Nitric oxide
NSAID	Nonsteroidal anti-inflammatory drug
PI3K	Phosphoinositide 3-kinase
PKB	Protein kinase B
PTZ	Pentylenetetrazol
PU	Peptic ulcer
RBC	Red blood cells
ROS	Reactive oxygen species
SAR	Structure-activity relationship
SNO	Esophageal carcinoma
SOD	Superoxide dismutase
TC_50_	Toxic concentration 50%
TNF-*α*	Tumor necrosis factor-*α*
WBC	White blood cells
ZOI	Zones of inhibition
